# Microwave-assisted organic synthesis of nucleoside ProTide analogues†

**DOI:** 10.1039/c9ra01754b

**Published:** 2019-06-27

**Authors:** Cinzia Bordoni, Cecilia Maria Cima, Elisa Azzali, Gabriele Costantino, Andrea Brancale

**Affiliations:** School of Pharmacy and Pharmaceutical Sciences Redwood Building, King Edward VII Avenue CF10 3NB Cardiff UK brancalea@cardiff.ac.uk; P4T Group, Dipartimento di Farmacia, University of Parma Parco Area delle Scienze 27/A Parma 43124 Italy

## Abstract

A microwave enhanced synthesis of prodrug nucleotide (ProTide) analogues is presented. Comparison of conventional thermal heating reaction with microwave irradiation exemplifies the potential of the novel methodology herein presented for the selective 5′-phosphoramidate synthesis, without protection of the 3′ position in the ribonucleoside.

## Abstract 2

A microwave enhanced synthesis of prodrug nucleotide (ProTide) analogues is presented.

## Graphical abstract

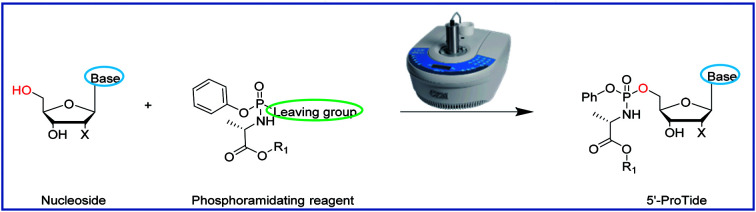

## Introduction

**Scheme 1:**
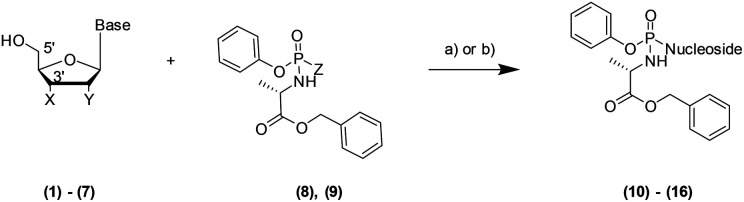
5′-ProTide traditional synthetic approaches^*a*^. ^*a*^Base: adenine, guanine, cytosine, thymine, uracil. X = –OH, –H, Y = –OH, –H. Reagent and conditions: (a) nucleoside, (8) (Z = *p*-NO_2_-Ph), *t*-BuMgCl, solvent, 0–25 °C, 12–48 hours, 10–42% or (b) nucleoside, NMI, (9) (Z = –Cl), solvent, 25 °C, 4–24 hours, 14–39%.

Microwave-Assisted Organic Synthesis (MAOS) has been widely used in the last 40 years in the organic chemistry field to solve problems such as low yield, long reaction time, side reactions and to synthesise libraries of compounds more efficiently. A number of articles report the optimisation of a variety of transformations exploiting the thermal/kinetic effect of the microwave irradiation (MWI) to enhance the reaction rate.^1,2^ A few examples include Mitsunobu reaction,^3^ Suzuki coupling,^4^ Buchwald–Hartwig amination.^5^ Prodrug nucleotide (ProTide) technology was originally designed by Professor Chris McGuigan and co-workers at Cardiff University in the early 1990s.^6^ A ProTide consists of a nucleoside where the negative charge on the monophosphate moiety has been masked with an amino acid ester and an aryl group (5′-aryloxyphosphoramidate) to deliver nucleotide analogues into the cell and to overcome nucleoside drug resistance.^7^ The ProTide approach has been successfully applied clinically: Sofosbuvir, Tenofovir alafenamide and Acelarin are clear examples of ProTide based drugs used in the treatment of different cancer and viral diseases.^8^ Interestingly, only one review drives the attention on the challenges in their synthetic preparation.^9^ Herein, we focus particularly on the final step of the ProTide synthesis: the nucleoside phosphoramidation, such as the coupling of the 5′-hydroxy moiety in the ribonucleoside with the phosphoramidating reagent. The two synthetic approaches commonly applied to perform this coupling are reported in Scheme 1.^10^ The first approach (Scheme 1, condition (a)) relies on the use of a strong base to activate the nucleophile according to the Uchiyama method.^11^ In the second method (Scheme 1, condition (b)), activation of the phosphoramidochloridate is performed by *N*-1-methylimidazole (NMI).^9,12^

Although both methodologies have been extensively used to synthesise several nucleoside prodrugs, a few limitations occur: poor solubility of the parent nucleoside; low yield (10–42%);^13^ longer reaction time (4–48 hours);^8,14,15^ lack of 5′-selectivity which leads to the formation of unwanted 3′,5′-*O*,*O*-phosphoramidates (bis) by-products, when X is an hydroxyl group and Y is either an hydroxy or hydrogen atom (Scheme 1).^9^

Also, appropriate protection of the sugar hydroxyl groups and/or of the nucleobase offers a solution to those limitations, although it is not amenable to high throughput chemistry. Additionally, protection/deprotection steps will require various conditions and protecting groups depending on the different ribonucleoside and phosphoramidating reagent. Besides, deprotection must be optimised to be compatible with the ProTide moiety. While common synthetic procedures for the synthesis of ProTide do not take into account the chirality at the phosphorous atom, a paper by Pertusati focuses on the development of a diastereoselective ProTide synthesis.^14^

Although, a suitable catalytic system to predominantly deliver the Sp diastereoisomer *via* copper catalysis was identified, the new synthetic methodology still suffers from long reaction time (8–12 h) and modest yields (12–66%).^14^

Sommadossi and co-workers reported the use of the Grignard method to synthesise a series of ProTide at 0 °C over long reaction time (15–18 h) and modest yields (16–55%).^15^ Recently, Simmons and co-workers reported an optimised procedure to regioselectively obtained the 5′-phosphoramidate prodrugs of the ribonucleoside: although their methodology was extensively validated on different pharmaceutically relevant ProTide, it still suffers from long reaction times (20–48 hours).^16^ In this work, we aimed to develop a MAOS methodology to obtain only the 5′-ProTide in good yield over shorter reaction time, without 3′,5′-*O*,*O*-phosphoramidates (bis-ProTide) by-products formation, and without additional protection/deprotection steps exploiting the cooling-while-heating (Power Max option)^17,18^ on a CEM Discover LabMate microwave synthesizer for both the two aforementioned synthetic procedures reported in Scheme 1. The cooling-while-heating MW technology further irradiates the reaction mixture with microwave power without overheating by simultaneously cooling down the reaction vessel with compressed air. The target temperature is constant during the microwave irradiation experiment. As aforementioned, the phosphoramidation reaction proceeds very slowly in conventional heating and with modest yield at 0 and 25 °C. Then, we monitored the profile of the conventionally heated reaction at higher temperature, such as 55 °C. Preliminary experiments demonstrated that in conventional heating, a further increase in temperature would have not been compatible with long reaction times required to observe the formation of the desired product. When the reaction mixture was irradiated under standard MWI method at 55 °C (fixed power, 100 W), there was not a beneficial effect on the formation of the desired product (microwave power absorbed by reaction mixture was 6 W). We then repeated the same experiment using the temperature fixed mode: we fixed the temperature at 55 °C and the MW irradiated the reaction mixture with as much power as the reaction mixture was able to absorb. Having observed the formation of the desired product, we then tried to optimise the MWI conditions. We initially exploited the effect of a 10 °C increase under MWI (fixed temperature mode, dynamic method) and monitor how the percentage of conversion to the desired product was influenced (microwave power absorbed in cooling-while-heating experiment was 100 W). Then, we investigated the effect of different solvents on both solubility and microwave power absorption (using both low and high microwave absorbing solvents), different reaction times and temperatures under conventional and MW heating. Reactions in conventional heating or under microwave irradiation were performed using the same number of equivalents of reagents, the same molarity for the solution of the reaction mixture, in closed vessel and under anhydrous conditions to avoid any effects of air/moisture stability of reagents, and evaporation of solvents. Finally, we compared the yields and the reaction times for each experiment in the same experimental conditions under both conventional heating and microwave irradiation.

## Results and discussion

To exemplify the potential of the application of the MWI to the nucleoside phosphoramidation, we started our studies using the protected adenosine (17) for the initial model reaction.^19^

The, we confirmed the compatibility of our microwave based approach using several phosphoramidating agents, exploring different leaving groups (*para*-nitrophenolate, chlorine atom, phenol) and ester moiety (benzyl, iso-propyl), as reported in Table 1.

**Table 1:** Protected adenosine phosphoramidate (22)–(23)^a^

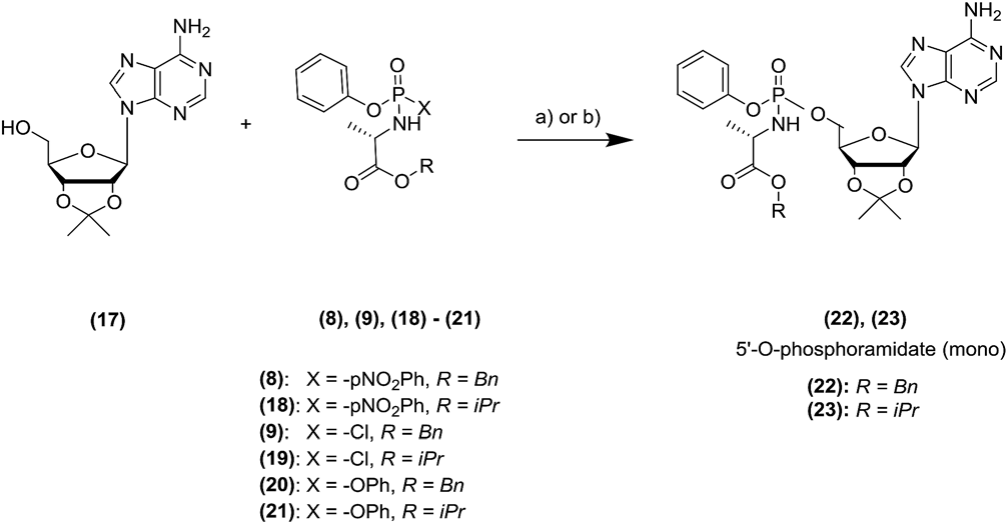						
Entry	Reagents	Solvent	Conventional heating (55 °C)		Microwave irradiation* (65 °C)	
			Time (min)	Yield** (%) (22) or (23)	Hold time (min)***	Yield** (%) (22) or (23)
1^a^	*t*-BuMgCl + (8)	THF/NMP	150	34 (22)	2.5	41 (22)
2^a^					30	81 (22)
3^a^	NMI + (9)	THF/NMP	120	55 (22)	15	28 (22)
4^b^	*t*-BuMgCl + (18)	THF/NMP	30	77 (23)	2	67 (23)
5^a^	NMI + (19)	THF/NMP	120	55 (22)	15	28 (22)
6^a^	*t*-BuMgCl + (19)	THF/NMP	15	52 (23)	2	36 (23)
7^a^	*t*-BuMgCl + (20)	THF/NMP	1440	29 (22)	60	42 (22)
8^a^	*t*-BuMgCl + (21)	THF/NMP	480	15 (23)	60	28 (23)

a Reaction conditions: (a) compound 17 (1 equivalent), *t*-BuMgCl (2 equivalent), compound 8 or 18 or 20 or 21 (2 equivalent), solvent (2 mL mmol^−1^); (b) compound 17 (1 equivalent), NMI (6 equivalent), compound 9 or 19 (2 equivalent), solvent (2 mL mmol^−1^). For both conditions, ratio THF/NMP is 4.8 : 1. *Microwave PowerMax set at 300 W. Maximum microwave power absorbed by reaction mixture 100 W. **Yields were calculated on the isolated product after automated flash column chromatography purification. ***Hold time reported here does not include 25 s of ramping time to go from 25 °C to target temperature and 50 s to cool down the reaction vessel at 25 °C after MWI.

Initially, we used a near-stoichiometric amount of Grignard reagent (2 equivalents) and a mixture of tetrahydrofuran (THF) and *N*-methyl-2-pyrrolidone (NMP).^11^ We compared the yield achieved in the conventional and MWI heating modes (Table 1).

Under MWI, we observed a remarkable reduction in the reaction times for all the different phosphoramidating reagents (8, 9, 18–21) and mostly also an improvement in the isolated yield. Encouraged by these results, we explored the cooling-while-heating MWI using different nucleosides in comparison to conventional heating reactions. Briefly, we initially used the same conditions as the ones applied to the protected adenosine 17 for both conventional heating and MWI experiments in terms of reagent stoichiometry and solvent system. When we observed a lack of reactivity or longer reaction times under MWI, we screened a different panel of conditions. The different parameters we investigated on the unprotected nucleosides (1–7) include: the stoichiometry of the Grignard reagent to ensure the O-selective condensation; the solvent to improve solubility of the parent nucleoside and maximise the absorbance of the microwave power using solvents with high tan *δ*; temperature in the MWI reaction to enhance the yield, while simultaneously shortening the reaction time. Detailed conditions and results of the optimisation study are listed in Section 3 of the ESI.† Here in Table 2, we report the best conditions for both synthetic methodologies as a comparison of the MWI against conventional heating mode for seven nucleosides (1–7). Initially, we applied the conditions used in the protected adenosine (17), to the unprotected nucleoside (1). Although yields were comparable, a 30-fold shorter reaction time was observed under MWI.

Nucleoside phosphoramidates (10–16) and by-products (24)^a^

**Table:** 

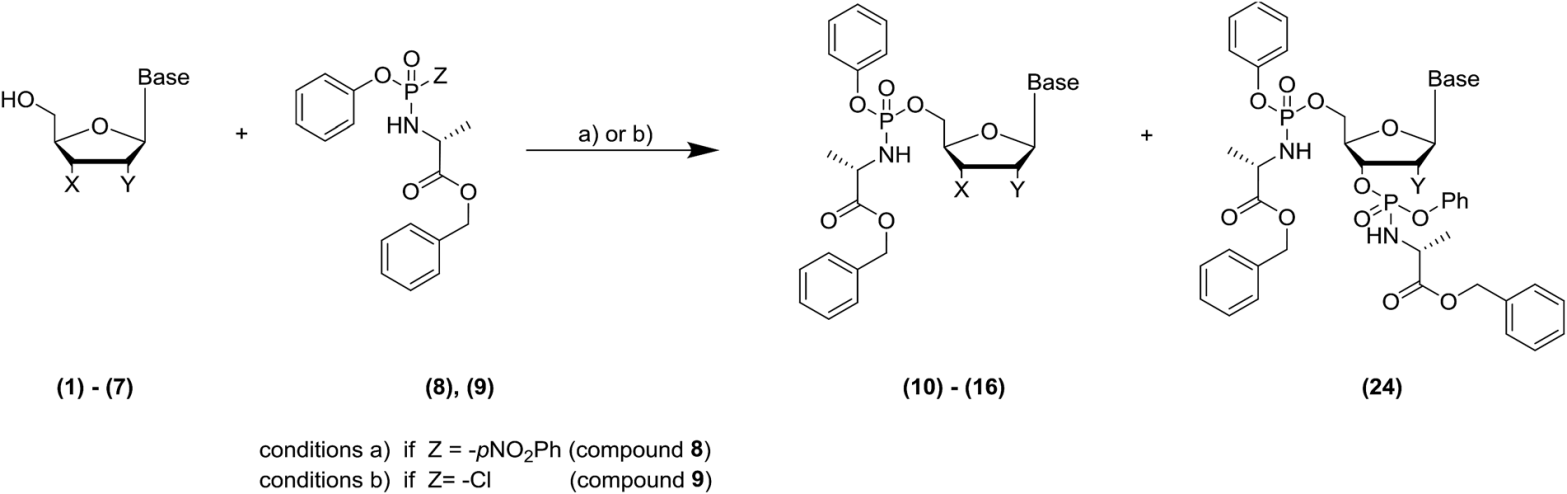					
Base	Nucleoside	X	Y	5′-ProTide	3′,5′-ProTide
Adenine	(1)	–OH	–OH	(10)	—
Cytosine	(2)	–OH	–OH	(11)	—
Cytosine	(3)	–H	–H	(12)	—
Guanine	(4)	–OH	–OH	(13)	—
Uracil	(5)	–OH	–OH	(14)	—
Thymine	(6)	–OH	–OH	(15)	(24)
Thymine	(7)	–H	–H	(16)	—

a Reagent and conditions: (a) *t*-BuMgCl (3 equivalents), (8), solvent (0.1 M. For THF/pyridine or THF/NMP: ratio was 4.8 : 1); (b) NMI (6.3 equivalents), (9), solvent (0.1 M. For THF/pyridine or THF/NMP: ratio was 4.8 : 1). *In this case, 0.5 equivalent of *t*-BuMgCl were added for 4 times (total of 2 equivalents). **Yield determined as percentage of conversion of the parent nucleoside in the desired mono substituted product and/or undesired bis substituted product by HPLC/UPLC analysis. ***Hold time reported here does not include 25 s of ramping time to go from 25 °C to target temperature and 50 s to cool down the reaction vessel at 25 °C after MWI.

**Table:** 

Entry	Nucleoside	Solvent	Conventional heating (55 °C)				Microwave irradiation				
			Time (min)	Yield (%)**			Hold time (min)***	Temperature (°C)	Yield (%)**		
				Nucleoside	5′-ProTide	5′,3′-ProTide			Nucleoside	5′-ProTide	5′,3′-ProTide
1^a^	(1)	THF/NMP	60	58 (1)	42 (10)	—	2	65	60 (1)	40 (10)	—
2^b^	(1)	THF/pyridine	180	45 (1)	55 (10)	—	20	85	38 (1)	61 (10)	
3^a^	(2)	DMF	200	48 (2)	51 (11)	—	25	65	19 (2)	81 (11)	—
4^b^	(2)	THF/pyridine	300	44 (2)	55 (11)	—	25	65	89 (2)	11 (11)	—
5^a^	(3)	DMF	135	1 (3)	99 (12)	—	5	75	19 (3)	81 (12)	—
6^b^	(3)	THF	300	47 (3)	53 (12)	—	10	65	10 (3)	90 (12)	—
7^a^	(4)	DMF	450	34 (4)	66 (13)	—	10	75	12 (4)	88 (13)	—
8^b^	(4)	THF/pyridine	1440	51 (4)	49 (13)	—	35	65	87 (4)	13 (13)	—
9^a^	(5)	THF/NMP	320	62 (5)	38 (14)	—	15	65	62 (5)	38 (14)	—
10^b^	(5)	THF	350	35 (5)	65 (14)	—	35	65	46 (5)	54 (14)	—
11^a,^*	(6)	THF/NMP	120	16 (6)	45 (15)	39 (24)	60	65	54 (6)	43 (15)	3 (24)
12^b^	(6)	THF	240	13 (6)	73 (15)	14 (24)	5	65	1 (6)	70 (15)	29 (24)
13^a^	(7)	DMF	150	2 (7)	98 (16)	—	1	65	19 (7)	81 (16)	—
14^b^	(7)	THF	155	16 (7)	84 (16)	—	3	65	11 (7)	89 (16)	—

Furthermore, the MWI mode did not affect selectivity towards 5′-product formation since bis-product was not detected (Table 2, entry 1). In the NMI method, THF and pyridine solvent mixture furnished the target 5′-product (10) in good yield in 20 minutes (Table 2 – entry 2). Although, pyridine tan *δ* is not reported in the literature, we believe its beneficial effect on the reaction mixture under MWI can be explained by its polar nature. To solve solubility limitation in the cytidine based ProTide synthesis, we focus our effort on the identification of a suitable solvent system. Cytidine proved to be insoluble in several solvent systems, such as 1,4-dioxane, THF alone and in co-mixture with NMP or pyridine. The use of the polar and high microwave coupling solvent *N*,*N*-dimethylformamide (DMF, tan *δ* = 0.161) considerably improved solubility and increased the yield (Table 2, entry 3), with a 10-fold shortening of the reaction time under MWI. In the NMI method, solubility of the ribonucleoside (2) was only slightly improved using a solvent mixture of THF/pyridine, and the yield dramatically decreased (Table 2, entry 4). Driven by the results achieved with cytidine, we further confirmed the beneficial effect of DMF also on the 2′,3′-dideoxycytidine (3). As expected, yield steadily improved in the microwave irradiation reaction for both synthetic approaches. Particularly because of the enhanced solubility and lack of 2′,3′ hydroxyl groups, in the Grignard method with a 20 °C increase in temperature under MWI, the desired product 12 was formed in an excellent yield and with a 27-fold reduction of the reaction time (Table 2, entry 5). Interestingly, in the NMI method, a 10 °C increase in the MWI reaction resulted in a 30-fold shorter reaction time (Table 2, entry 6). Then, we focused on the conditions optimisation for guanosine (4). To promote the kinetic of the reaction, we sought to simultaneously increase the equivalents of Grignard reagent and the reaction temperature in the microwave heating system, using DMF as solvent. In this case, we observed an excellent percentage of conversion of the parent nucleoside into the desired 5′-ProTide (13) (Table 2, entry 7) achieving a considerable decrease in the reaction time. Since guanosine (4) was unreactive in the NMI conditions, we simultaneously activated the 5′-hydroxy group on the ribose using the *tert*-butyl magnesium chloride and the phosphoramidating reagent upon treatment with the NMI (ESI, S30,†Table 2, entry 14) without any success. To the best of our efforts, we could observe only a 13% conversion in the desired compound (13), over a significantly shorter (10 minutes) reaction time (Table 2, entry 8). When we irradiated uridine (5) with microwave power, in the Grignard driven method, using THF/NMP as solvent, we successfully achieved the formation of the mono product (14) in only 15 minutes (20-fold reduction in reaction time) and good yield (38%). Then, in the NMI based methodology, the conversion of the parent nucleoside in the desired product (14) was achieved in good yield (Table 2, entry 10) with a 10-fold reduction of the reaction time (Table 2 – entries 9 and 10). With thymidine (6), the use of different solvent systems proved to be unsuccessful as increasing the Grignard reagent equivalents. Hence, we decided to clarify whether the rate of addition of both base and phoshoramidating reagent may play an important role in driving the formation of the 5′-phosphoramidate. The portion wise addition of both the base and the phosphoramidating reagent in the Grignard method, selectively promotes the formation of the desired product (15), halving the reaction time in the MW irradiated reaction, limiting the formation of the bis product (24, Table 2 – entry 11). In the NMI method, we achieved the formation of the target compound (15) in comparable yield between the two heating systems, but with a remarkable decrease of the reaction time (48-fold shorter) in MWI optimised experiment (Table 2, entry 12). In the 2′-deoxythymidine (7) optimisation of the Grignard method, the use of DMF allows to overcome the solubility issues and to increase the absorbance of microwave power. Then, we shortened the reaction time by increasing the reaction temperature in the MW vessel. In the NMI based method, we observed formation of the mono product (16) in excellent yield (81–89%) with a 150–52 fold shortening of the reaction time for both methodologies. To the best of our knowledge, this was the first application of the microwave irradiation to the phosphoramidation reaction in the synthetic preparation of the ProTides.

## Conclusions

Microwave assisted synthesis has been successfully applied to a number of ribonucleoside and deoxyribonucleoside bearing different bases and using variously substituted phosphoramidating reagents. We achieved formation of the desired 5′-ProTide in good to excellent (13–97%) yields, with a considerably shortening of the reaction time (2–150 times decrease). Additionally, with thymidine nucleosides (Table 2 – entries 3–7), microwave irradiation proved to promote the selective 5′-phosphoramidation contrary to the thermal heating where the bis by-side product was predominantly formed. This optimised methodology may be applied to facilitate the synthesis of novel pharmaceutically relevant ProTides in the future.

## Conflicts of interest

There are no conflicts to declare.

## Supplementary Material

RA-009-C9RA01754B-s001
