# Supplementary material for: Microwave-assisted organic synthesis of nucleoside ProTide analogues
Source: RSC Adv. 2019 Jun 27;9(35):20113–7. doi: 10.1039/c9ra01754b (PMC9065484; doi:10.1039/c9ra01754b)
Supplement: RA-009-C9RA01754B-s001 [file RA-009-C9RA01754B-s001.pdf]

Electronic Supplementary Material (ESI) for RSC Advances.
This journal is © The Royal Society of Chemistry 2019

Supporting information

Microwave-assisted organic synthesis of nucleoside analogues

Cinzia Bordoni,[1] Cecilia Maria Cima,[1] Elisa Azzali,[2] Gabriele Costantino,[2] Andrea Brancale[1]

[1]School of Pharmacy and Pharmaceutical Sciences, Redwood Building, King Edward VII Avenue, CF10 3NB, Cardiff.

[2]P4T Group, Dipartimento di Farmacia, University of Parma, Parco Area delle Scienze 27/A, Parma, 43124, Italy.

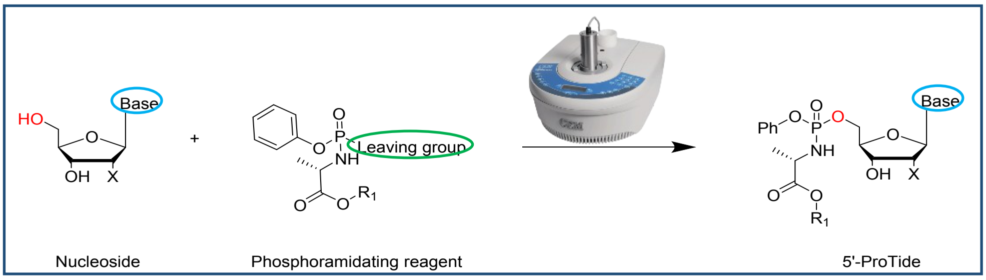

Table of contents

1	General information	S3
2	Standard procedures	S5
3	Reaction conditions and characterisation	S7
	3.1 Adenosine	S7
	3.1.1 Conditions for the optimisation study	S7
	3.1.2 HPLC spectra	S8
	3.1.3 Spectroscopic and spectrometric characterisation	S14
	3.2 Cytidine	S15
	3.2.1 Conditions for the optimisation study	S15
	3.2.2 HPLC spectra	S16
	3.2.3 Spectroscopic and spectrometric characterisation	S22
	3.3 2’,3’-Dideoxycytidine	S23
	3.3.1 Conditions for the optimisation study	S23
	3.3.2 UPLC spectra	S24
	3.3.3 Spectroscopic and spectrometric characterisation	S29
	3.4 Guanosine	S30
	3.4.1 Conditions for the optimisation study	S30
	3.4.2 HPLC spectra	S31
	3.4.3 Spectroscopic and spectrometric characterisation	S37
	3.5 Uridine	S38
	3.5.1 Conditions for the optimisation study	S38
	3.5.2 HPLC spectra	S39
	3.5.3 Spectroscopic and spectrometric characterisation	S45
	3.6 Thymidine	S46
	3.6.1 Conditions for the optimisation study	S46
	3.6.2 HPLC spectra	S47
	3.6.3 Spectroscopic and spectrometric characterisation	S54
	3.7 3’-Deoxythymidine	S56
	3.7.1 Conditions for the optimisation study	S56
	3.7.2 UPLC spectra	S57
	3.7.3 Spectroscopic and spectrometric characterisation	S61
4	Spectroscopic characterisation of compounds (8), (9), (17) – (23)	S62
5	NMR data	S71
6	Bibliography	S89

1. General information

All the chemicals, reagents and solvents were purchased from SIGMA Aldrich or Alfa Aesar without further purification or purified by standard techniques. All reactions were carried out under nitrogen in oven-dried glassware. Organic solutions were evaporated under reduced pressure using a Buchi rotary evaporator equipped with a water bath. Thin Layer Chromatography (TLC) was performed using silica gel plates (Merck Kieselgel $60F_{254}$), developed by the ascending method. After solvent evaporation, compounds were visualised by irradiation with UV light at 254 nm and 366 nm. Microwave reactions were conducted in a 10 mL glass vessel sealed with a plastic septum and place in the microwave cavity of the Discover Labmate CEM microwave reactor in closed vessel mode, irradiating at maximum power of 300W and setting the Power Max option on. Due to the size of the microwave cavity and of the microwave vial, an overall volume minor than 5 mL must be used to ensure homogeneous irradiation of the microwave power through the solution. Hence, it was not possible to perform the reactions in 1 mmol scale. Conventional heating mode reactions were performed in a two-necks round bottom flask equipped with a reflux condenser and placed in the oil bath. In the conventional thermally heated reaction, temperature was set at 55 °C, whereas in the MW irradiation experiments the initial reaction temperature was set at 65°C. Purification was performed by silica gel chromatography using silica gel 40-60 µm from Merck and the appropriate eluent mixture or using the Interchim PuriFlash 4000 automated column chromatography system using the Interchim cartridges of the appropriate size (10 - 100 g). ^{1}H-NMR, ^{13}C-NMR, ^{31}P-NMR spectra were recorded using a Bruker AVANCE (500 MHz, 125MHz and 202 MHz) spectrometer auto-calibrated to the deuterated solvent reference peak (used the applied solvent simultaneously as internal standard). TMS was used as an internal standard for ^{1}H-NMR, ^{13}C-NMR, ^{31}P-NMR (δ = 0 ppm). Chemical shifts (δ) are given in ppm (parts per million) relative to tetramethylsilane (used as internal standard, δ = 0 ppm) together with the relative assignment, the coupling constant ($J_{(H-H)}$ / Hz) and the multiplicity: singlet (s), doublet (d), triplet (t), quartet (q), multiplet (m), broad multiplet (bm). Low-resolution mass spectra were performed on Bruker Daltonics microTof-LC in positive or negative mode, atmospheric pressure ionization, electron spray ionization mass spectroscopy (ESI). All analytical high-performance liquid chromatography (HPLC) experiments were done on a Series 200 UV/Vis Detector provided with a System Controller SN4000, a pump Spectra System P4000 flow range of 0.1 to 10.0 ml/min and a maximum operating pressure of 6000psi (400 Bar), PerkinElmer Series 200 Column Oven controls, Series 200 UV/Vis Detector using a C18-Varian Pursuit (150 × 4.6 mm, 5 µM) reverse phase column. Samples were prepared by dissolving 1 mg in 5 mL acetonitrile and water solution (1:1), filtered using a 0.2-0.4 u syringe filters, at 254 nm. The reactions were monitored by HPLC using the eluents water (eluent A), methanol (eluent B), at two wavelengths (254 nm and 280 nm), under the following conditions: gradient from 100% → 70% of eluent A in 15 minutes, then to 100% of eluent B in 15 minutes (method 1). Analytical ultra-performance liquid chromatography (UPLC) experiments were done on Acquity UPLC H-Class Core System (Waters) provided with Acquity QDa Detector (Performance), Acquity UPLC PDA eLambda Detector, Acquity

H-Class with QDa, using a Acquity UPLC BEH C18 1.7µm (2.1x100mm) column and a Acquity BEH C18 1.7µM VANGUARD Pre-column. Samples were prepared by dissolving 5 mg of substance in 5 ml of water (solvent A) and acetonitrile (solvent B) solution (1:1), filtered using a 0.2-0.4 µm syringe filter under the following conditions:

1) method 1: gradient 99% of eluent A for 0,50 minutes, from 99% → 20% of eluent A in 0,90 minutes, 1,50 minutes at 100% of eluent B, 0,10 minutes to 99% of eluent A;
2) method 2: 0.10 minutes at 90% of eluent A, from 90% → 0% of eluent A in 2.60 minutes, 0.30 minutes at 100% of eluent B, from 0% → 90% of eluent A in 0.10 minutes.

On both system (HPLC and UPLC), the parent nucleoside and the phosphoramidating reagent were independently run and their retention time determined. Then, the peak of the parent nucleoside was used as reference when the reaction mixture was run to monitor the conversion of the nucleoside to the desired product. Upon completion of the reaction, the desired product was purified. The purified product was then confirmed by NMR and mass, and analysed by HPLC or UPLC to confirm its retention time and purity.

2. Standard procedures

Standard procedure A (Grignard method): reaction under conventional heating mode

In a closed 2 neck round bottom flask under N_2, nucleoside (**1-7**) (0.08g, 0.24 mmol) was dissolved in anhydrous solvent (7 mL/ mmol) and NMP (2.3 mL/mmol) under a nitrogen atmosphere and *tert*-butyl magnesium chloride 1M (0.49 mL, 0.49 mmol) in solvent (1mL/mmol) was added dropwise at room temperature. After stirring 10 minutes, a solution of the phosphoramidating reagent (2 equivalents) in anhydrous solvent (2 mL/mmol) was added slowly. The reaction mixture was heated to 55 °C for the proper reaction time, then cooled to room temperature, poured into a 10% aqueous solution of NH_4Cl (10 mL) and extracted with DCM (3x10mL), the combined organic layer was washed with water (20 mL) and brine (20 mL), dried over $MgSO_4$, filtered and evaporated under reduced pressure. The residue was purified by flash column chromatography on silica gel using DCM to DCM/MeOH as elution system to give the title compound as a colourless wax.

Standard procedure A (Grignard method): reaction under microwave irradiation (MWI)

Nucleoside (**1-7**) (0.08g, 0.24 mmol) was dissolved in anhydrous THF (7 mL/ mmol) and NMP (2.3 mL/mmol) in a 10 mL sealed microwave tube under a nitrogen atmosphere and *tert*-butylmagnesium chloride 1M (0.49 mL, 0.49 mmol) in solvent (1mL/mmol) was added dropwise at room temperature. After stirring 10 minutes, a solution of the phosphoramidating reagent (2 equivalents) in anhydrous solvent (2mL/mmol) was added slowly. The microwave vial was then placed into the microwave cavity in closed vessel mode. The reaction mixture was stirred under MWI for the proper reaction time (300W, 25 psi, Power Max mode on), then cooled to room temperature, poured into a 10% aqueous solution of NH_4Cl (10 mL) and extracted with DCM (3x10 mL), the combined organic layer was washed with water (20 mL) and brine (20 mL), dried over $MgSO_4$, filtered and evaporated under reduced pressure. The residue was purified by flash column chromatography on silica gel using DCM to DCM/MeOH (100% → 90%/ 10%) as elution system to give the title compound as a colourless wax.

Standard procedure B (NMI method): reaction under conventional heating mode

Nucleoside (**1-7**) (0.08g, 0.24 mmol) was dissolved in anhydrous solvent (7 mL/ mmol) and NMP (2.3 mL/mmol) under a nitrogen atmosphere and NMI (6 equivalents) was added dropwise at room temperature. After stirring 30 minutes, a solution of the phosphoramidating reagent (2 equivalents) in anhydrous solvent (2 mL/mmol) was added slowly. The reaction mixture was heated to 55 °C for the proper reaction time, then cooled to room temperature, poured into water (10 mL) and extracted with DCM (3x10mL), the combined organic layer was washed with water (20 mL) and brine (20 mL), dried over $MgSO_4$, filtered and evaporated under reduced pressure. The residue was purified by flash column chromatography on silica gel using DCM to DCM/MeOH as elution system to give the title compound as a colourless wax.

Standard procedure B (NMI method): reaction under microwave irradiation (MWI)

Nucleoside (**1-7**) (0.08g, 0.24 mmol) was dissolved in anhydrous solvent (7 mL/ mmol) and NMP (2.3 mL/mmol) under a nitrogen atmosphere and NMI (6 equivalents) was added dropwise at room temperature. After stirring 30 minutes, a solution of the phosphoramidating reagent (2 equivalents) in anhydrous solvent (2 mL/mmol) was added slowly. The microwave vial was then placed into the microwave cavity in closed vessel mode. The reaction mixture was stirred under MWI for the proper reaction time (300W, 25 psi, Power Max mode on), then cooled to room temperature, poured into water (10 mL) and extracted with DCM (3x10 mL), the combined organic layer was washed with water (20 mL) and brine (20 mL), dried over $MgSO_4$, filtered and evaporated under reduced pressure. The residue was purified by flash column chromatography on silica gel using DCM to DCM/MeOH (100% → 90%/ 10%) as elution system to give the title compound as a colourless wax.

3. Reaction conditions and characterisation

3.1 Adenosine

3.1.1 Conditions for the optimisation study

Table 1. Adenosine phosphoramidate and by-products. *Reagent and conditions*: a) *t*-BuMgCl (2-4 equivalents), solvent; b) NMI (6.3 equivalents), solvent. In blue and bold, best conditions presented in the main paper.

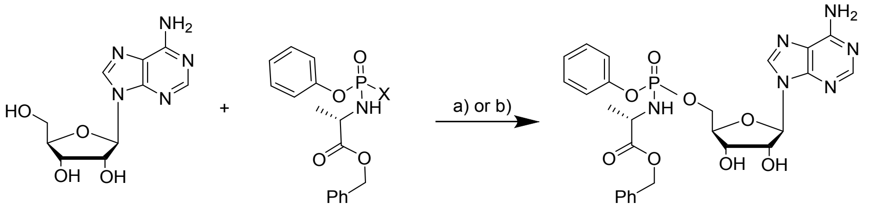

(1) **(8), (9)** **(10)**

(8): X = -pNO$_2$Ph *reagents and condition* a)
(9): X = -Cl *reagents and condition* b)

5'-O-phosphoramidate

				**Conventional heating (55 °C)**			**Microwave irradiation**			
	**entry**	**Reagents**	**Solvent**	**Time (min)**	**Yield (%) (1)**	**Yield (%) (10)**	**Hold time (min)**	**T (°C)**	**Yield (%) (1)**	**Yield (%) (10)**
**Grignard method, (a)**	1	*t*-BuMgCl (2 Eq) + (**8**)	THF	1200	53	47	60	65	72	28
	2	*t*-BuMgCl (2 Eq) + (**8**)	THF/ NMP	120	65	35	30	65	68	32
	**3***	***t*-BuMgCl (3 Eq) + (8)**	**THF/ NMP**	**60**	**58**	**42**	**2**	**65**	**60**	**40**
	4	*t*-BuMgCl (4 Eq) + (**8**)	THF/ NMP	60	degradation		2	65	degradation	
**NMI method, (b)**	**5***	**NMI + (9)**	**THF/ pyridine**	**180**	**45**	**55**	35	65	75	25
	6**	NMI + (**9**)	THF/ pyridine				**20**	**85**	**38**	**61**

* Conversion of the parent nucleoside into the desired 5'-protide was calculated on the purified compound, after column chromatography.

** Conversion of the parent nucleoside into the desired 5'-protide was calculated on UPLC analysis (method 2).

3.1.2 HPLC spectra

Adenosine (**1**) – HPLC

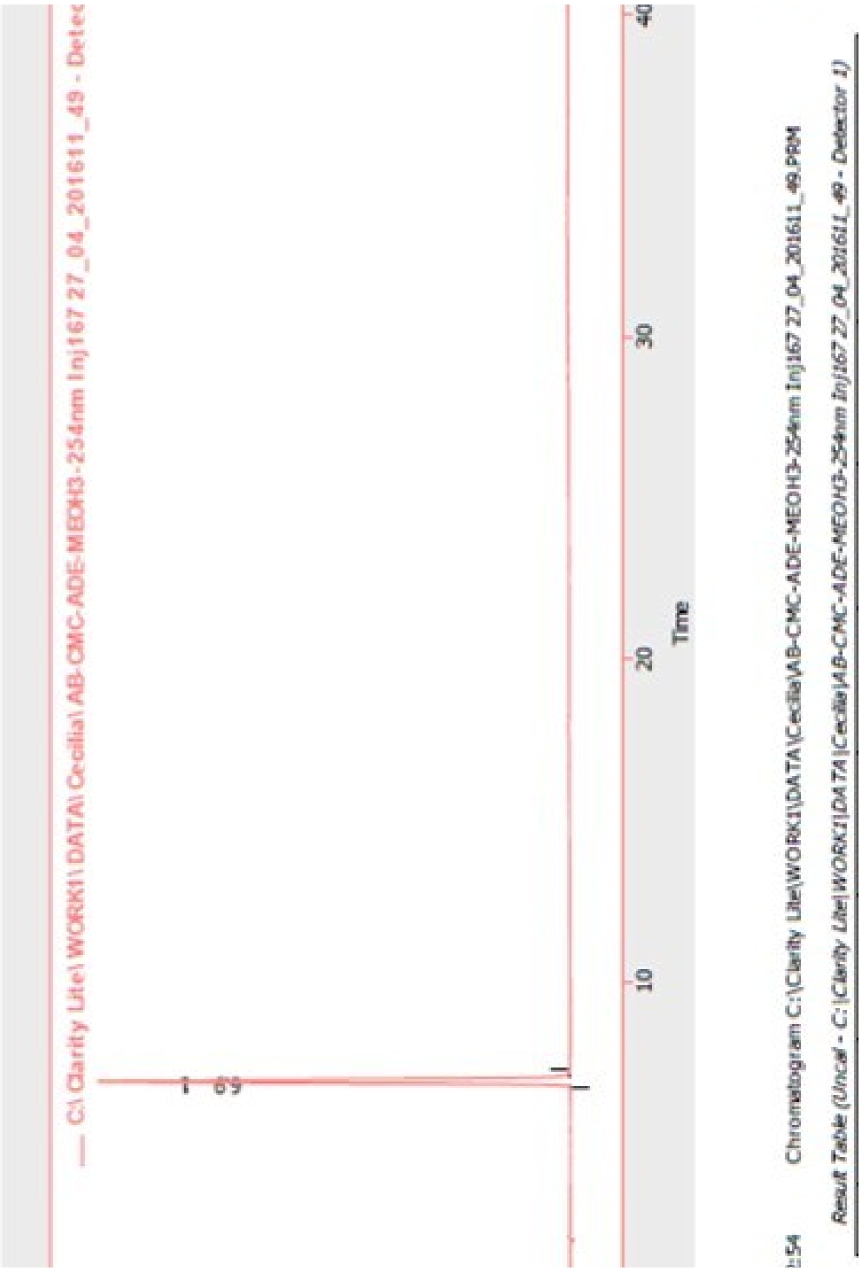

Compound (**10**) – HPI

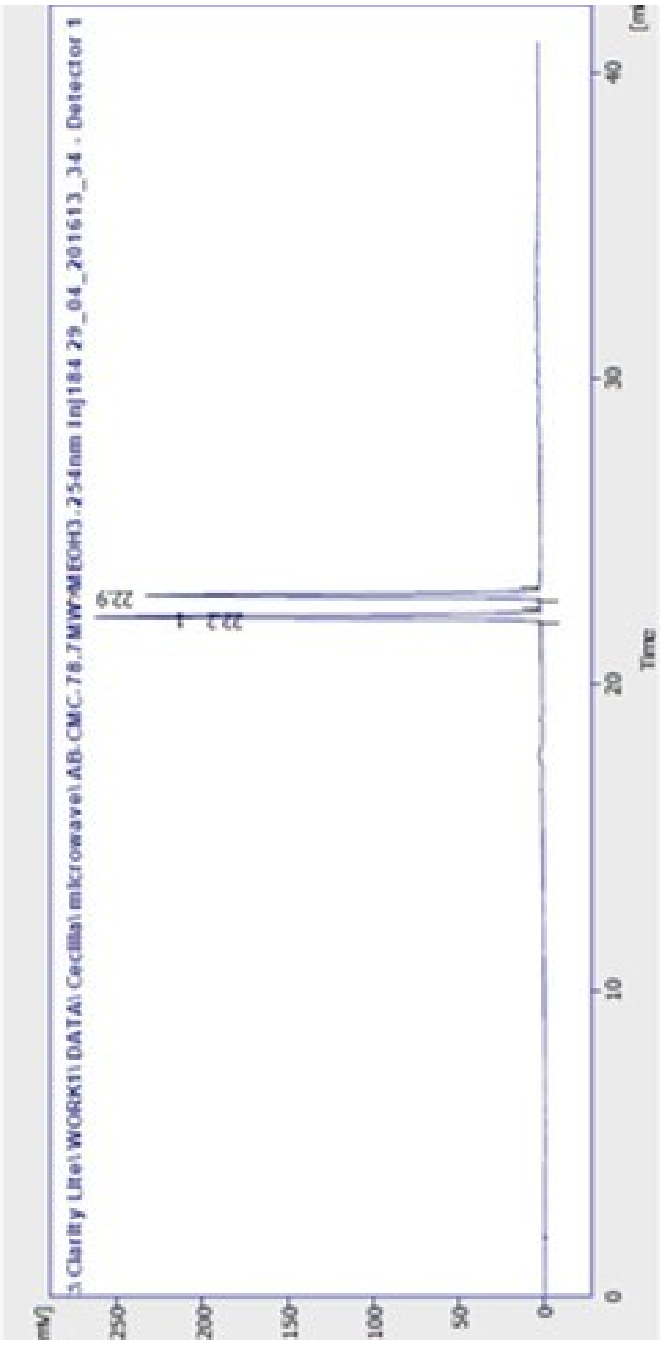

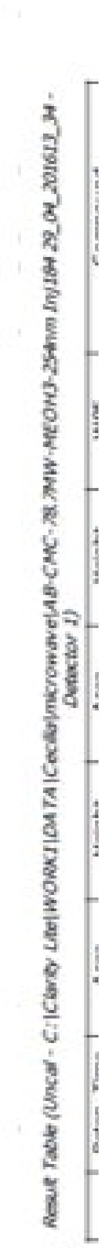

Reaction in Table

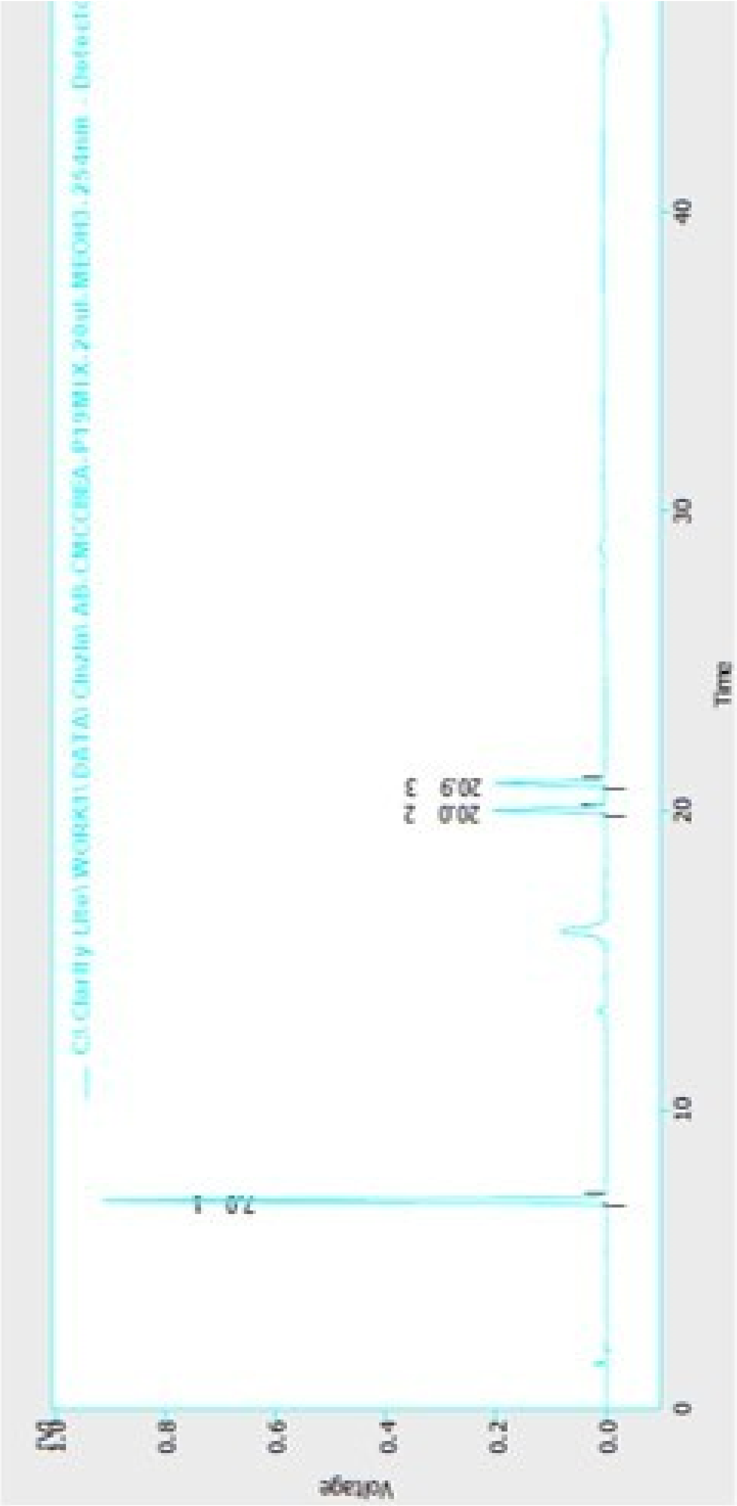

Result Table (Uncal - C:\Clarity Lite\WORK1\DATA\Cindia\AB-CMCCBEA-P19MIX-30uI-MEOH3-254nm - Detector 1)

	Reten. Time [min]	Area [mV.s]	Height [mV]	Area [%]	Height [%]	W05 [min]	Compound Name
1	6.957	7069.652	909.420	67.7	69.5	0.12	
2	20.016	1760.075	200.864	16.9	15.3	0.14	
3	20.904	1609.187	198.703	15.4	15.2	0.13	

Reaction in Table 1 –entry 3 (conventional heating)

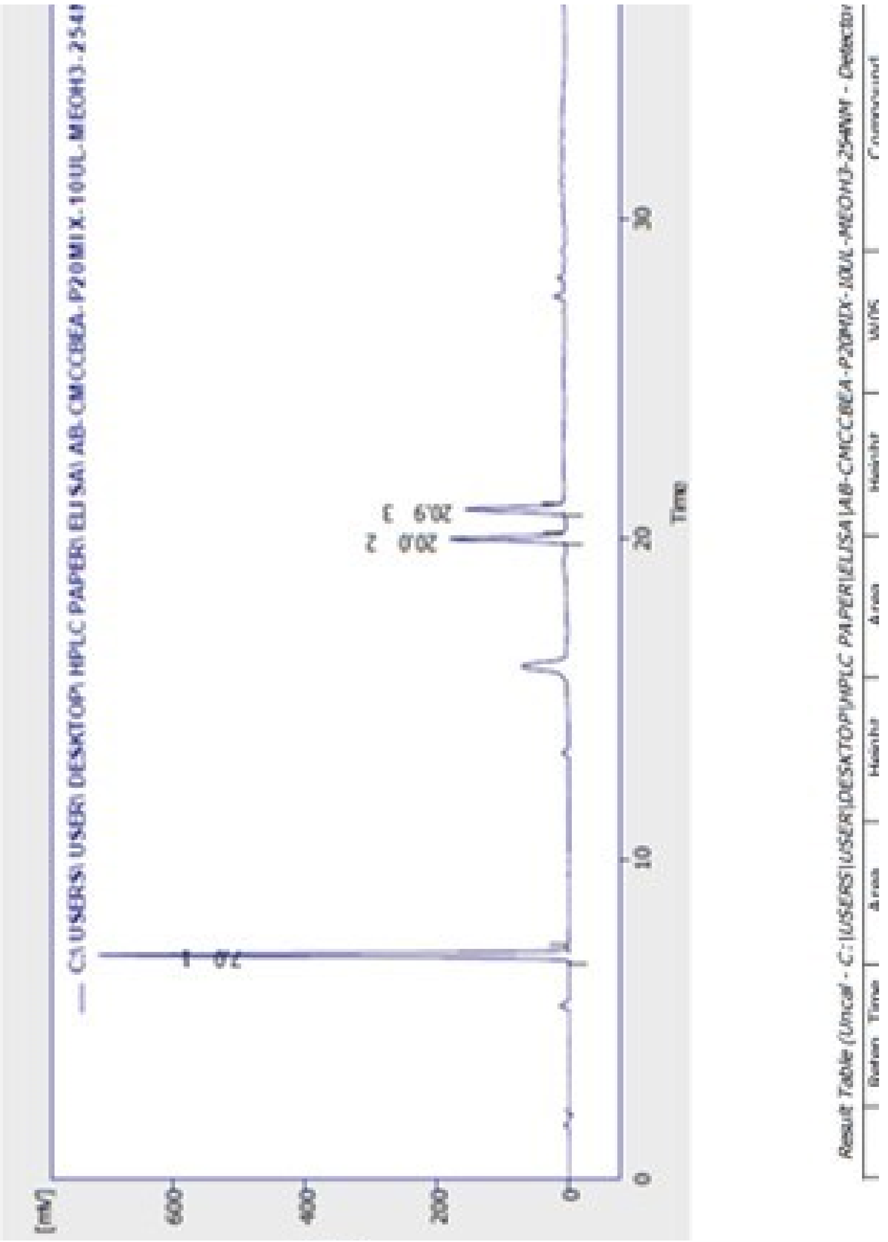

Reaction in Table 1 –entry 5 (conventional heating)

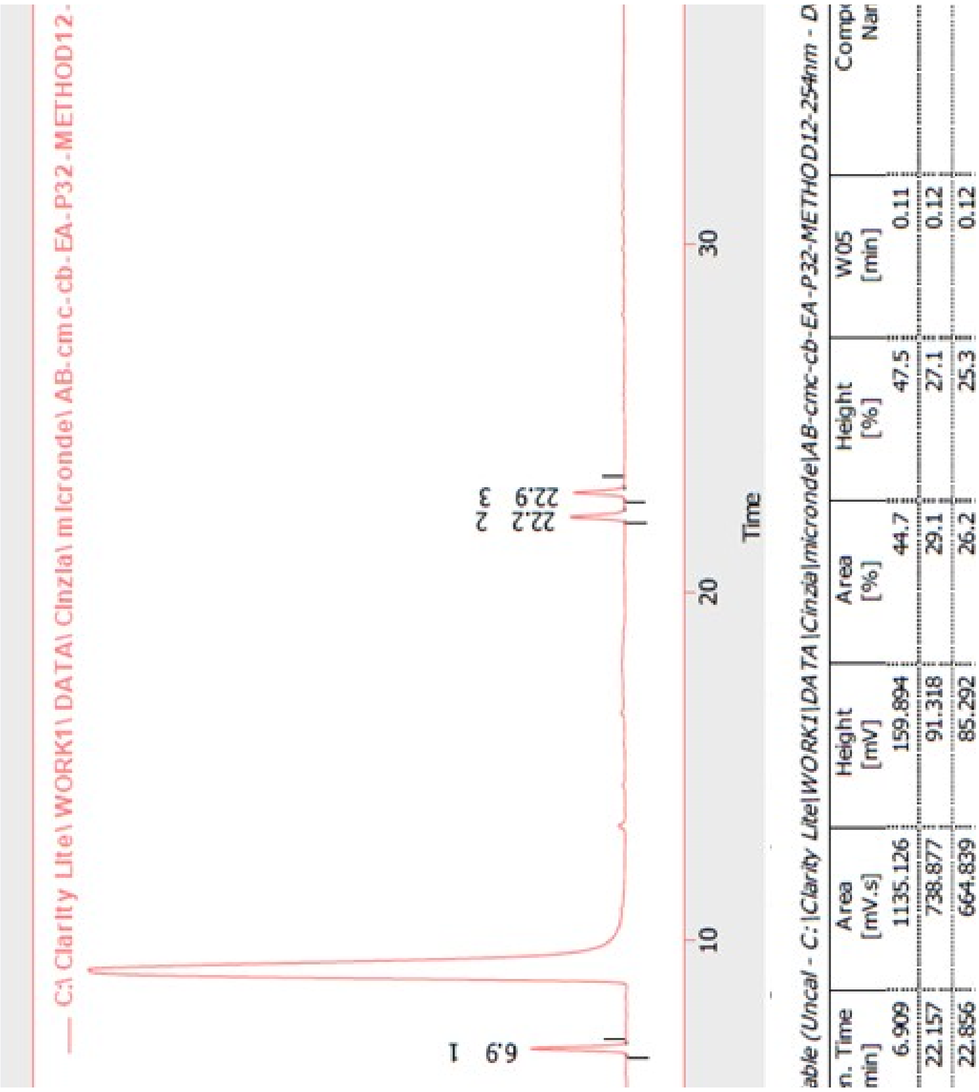

Reaction in Table 1 – entry 6 (microwave irradiation heating, UPLC data)

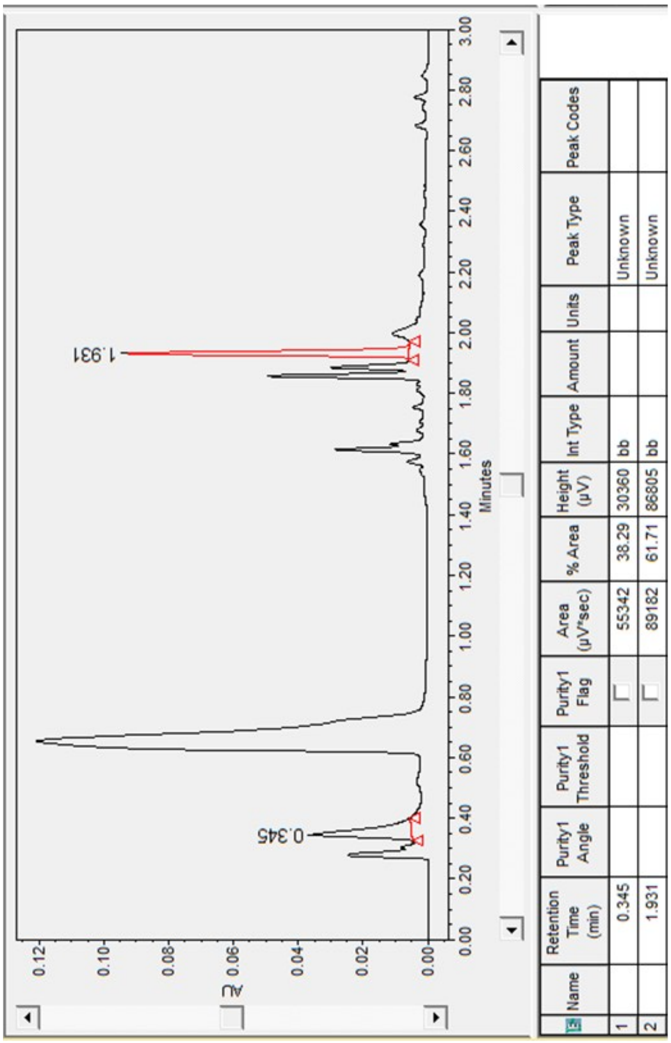

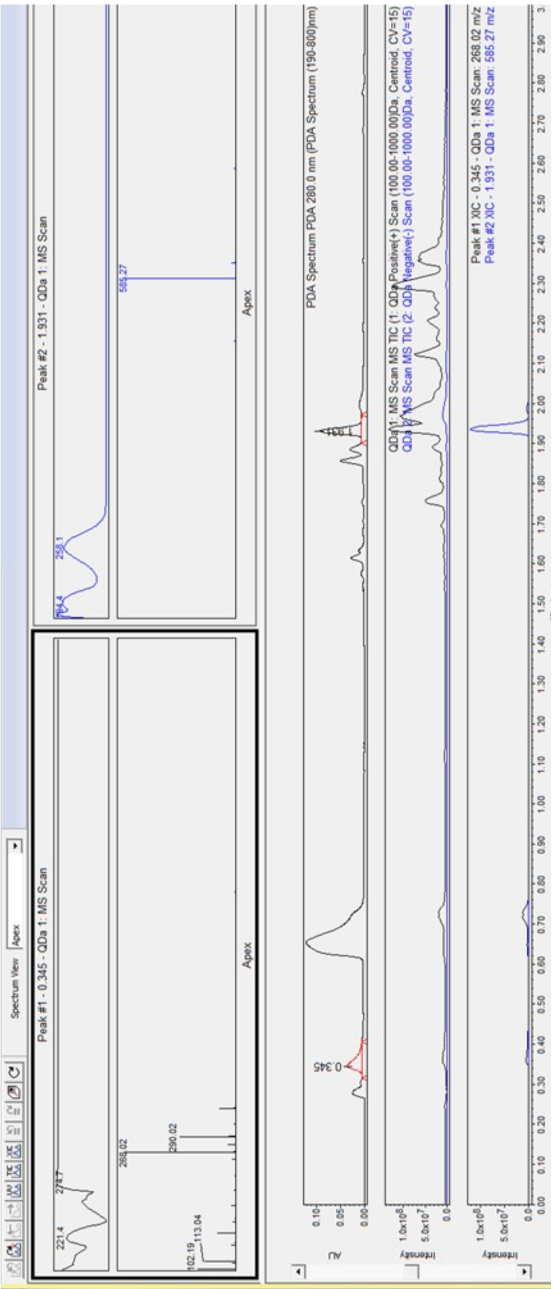

3.1.3 Spectroscopic and spectrometric characterisation

Adenosine-5'-O[phenyl-(benzyloxy-L-alaninyl)] phosphate (10)[4]

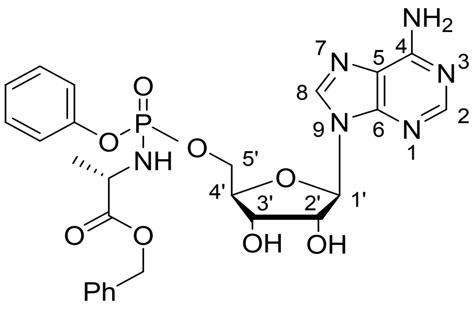

Procedure: standard procedures A and B

State: colourless wax (70 mg)

^{1}H-NMR (500 MHz, $CDCl_3$) δ 8.28 - 8.19 (m, 2H, -H_2, -H_8), 7.35 - 7.26 (m, 7H, -C_6H_5), 7.22-7.14 (m, 3H, -C_6H_5), 6.07 - 6.03 (m, 1H, -$H_{1'}$), 5.15 - 5.05 (m, 2H, -CH_2), 4.67 - 4.64 (t, J = 5.1 Hz, 1H, -$H_{2'}$), 4.44 - 4.37 (m, 2H, -$H_{5'}$), 4.36-4.28 (m, 1H,-$H_{3'}$), 4.27-4.24 (m, 1H, -NH), 4.04-3.91 (m, 1H, -$H_{4'}$), 3.98 (m, 1H, -CH), 1.33-1.28 (d, J = 7.2Hz, 3H, -CH_3) ppm.

^{13}C-NMR (126 MHz, $CDCl_3$) δ 173.69 (C, C-aromatic, C=O), 155.97 (C, C-aromatic, -C_2), 152.24 (CH, C-aromatic), 150.97 (C, C-aromatic, -C_5), 149.11 (CH, C-aromatic), 136.06 (C, C-aromatic), 129.50 (CH, C-aromatic), 128.00 (CH, C-aromatic), 124.78 (CH, C-aromatic), 102.19 (CH, C-aromatic), 119.29 (CH, C-aromatic), 88.56 (CH, C-aliphatic, $C_{1'}$), 83.25 (-CH, C-aliphatic, $C_{3'}$), 73.92 (-CH, C-aliphatic, $C_{4'}$), 70.18 (CH, C-aliphatic, $C_{2'}$), 66.47 (CH_2, C-aliphatic), 65.49 (CH, C-aliphatic, $C_{5'}$), 50.29 (CH, C-aliphatic), 19.01 (CH_3, C- aliphatic) ppm.

^{31}P-NMR (202 MHz, $CDCl_3$) δ 3.90, 3.66 ppm.

MS(ES)$^+$ m/z 607.2 $[M+ Na]^+$, 585.2 $[M+ H]^+$

3.2 Cytidine

3.2.1 Conditions for the optimisation study

Table 2. Cytidine phosphoramidate and by-products. *Reagent and conditions*: a) *t*-BuMgCl (3 equivalents), solvent; b) NMI (6.3 equivalents), solvent. In blue and bold, best conditions presented in the main paper.

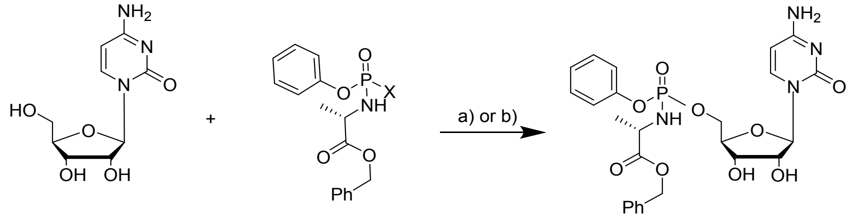

(2) **(8), (9)** **(11)**

(8): X = -pNO$_2$Ph *reagents and condition* a)
(9): X = -Cl *reagents and condition* b)

5'-O-phosphoramidate

				**Conventional heating (55 °C)**			**Microwave irradiation**			
	**entry**	**Reagents**	**Solvent**	**Time (min)**	**Yield (%) (2)**	**Yield (%) (11)**	**Hold time (min)**	**T (°C)**	**Yield (%) (2)**	**Yield (%) (11)**
**Grignard method, a)**	1*	*t*-BuMgCl (3 Eq) + (**8**)	THF	200	31	69	35	65	74	26
	**2***	***t*-BuMgCl (3 Eq) + (8)**	**DMF**	**250**	**48**	**52**	**25**	**65**	**19**	**81**
**NMI method, b)**	**3****	**NMI + (9)**	**THF/Pyridine**	**300**	**45**	**55**	**25**	**65**	**89**	**11**

* Conversion of the parent nucleoside into the desired 5'-protide was calculated on HPLC analysis (method 1).
** Conversion of the parent nucleoside into the desired 5'-protide was calculated on UPLC analysis (method 1).

3.2.2 HPLC spectra

Cytidine (**2**) – HPLC purity

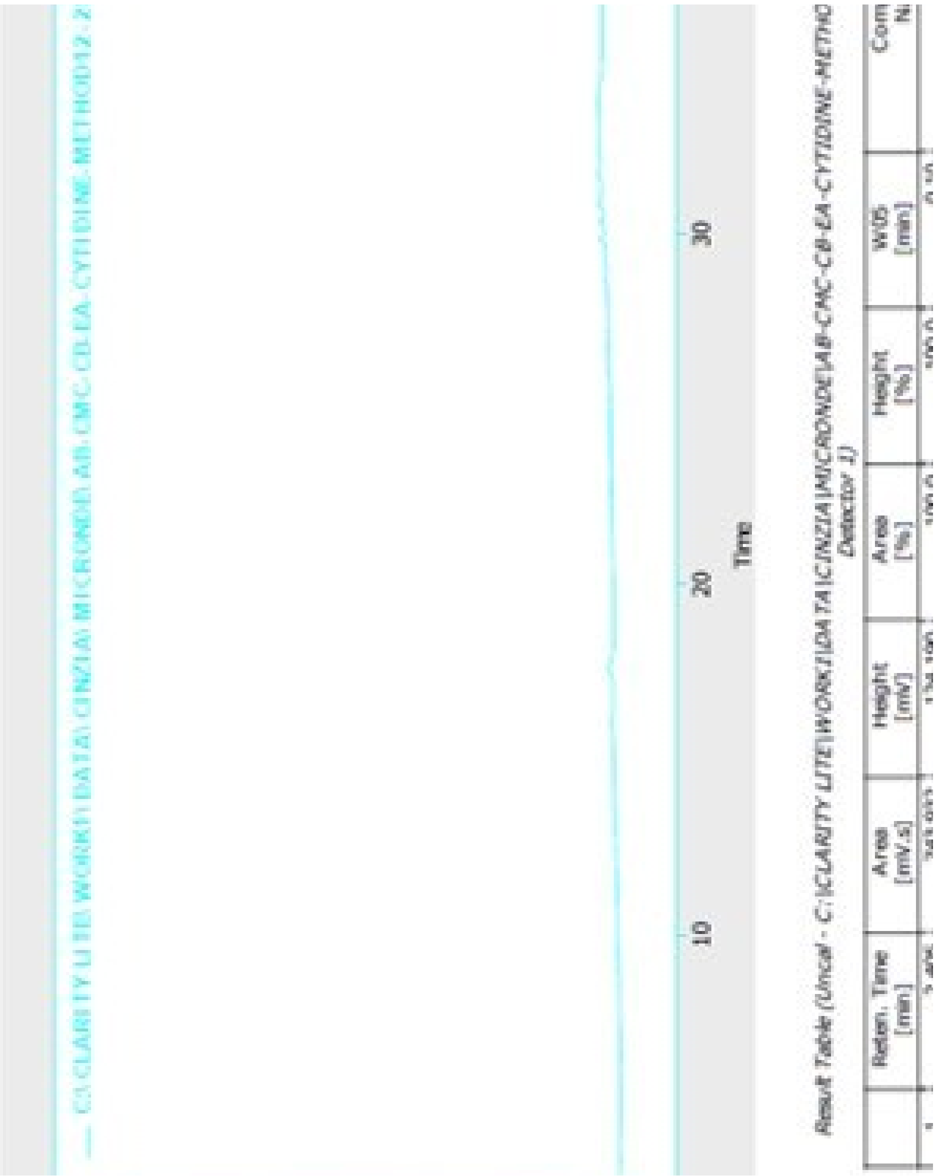

Compound (**11**) – UPLC purity

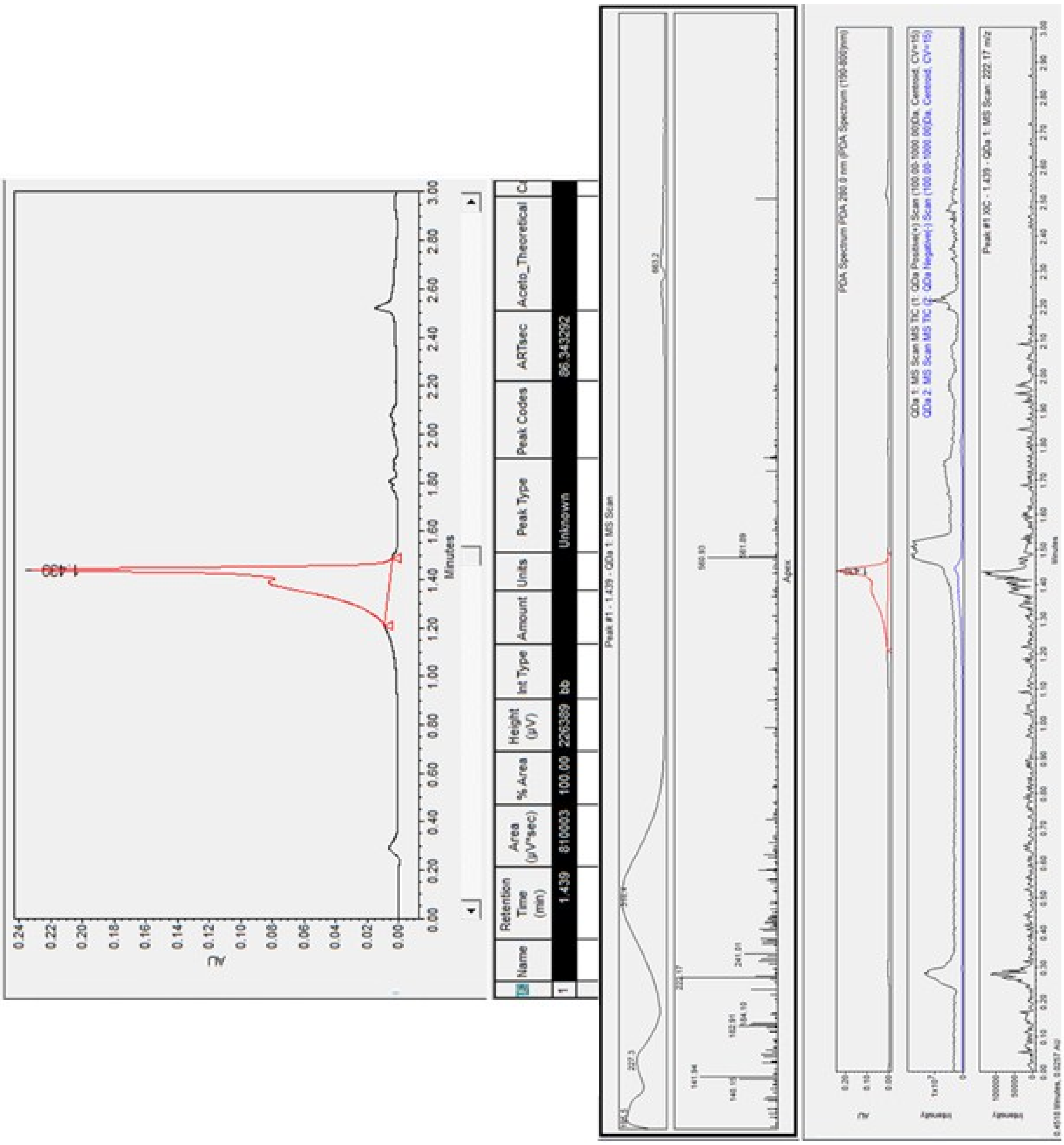

Reaction in Table 2 –entry 2 (conventional heating)

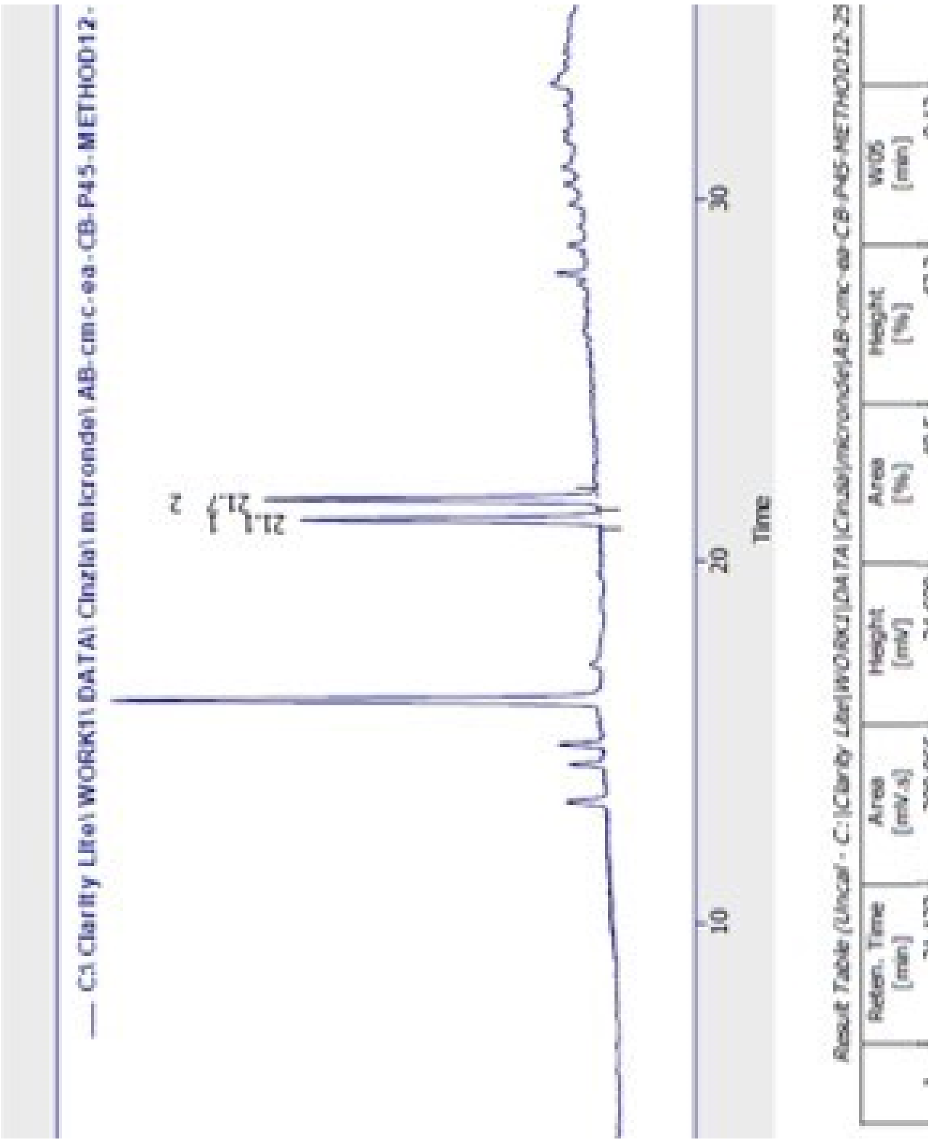

Reaction in Table 3 –entry 2 (microwave heating)

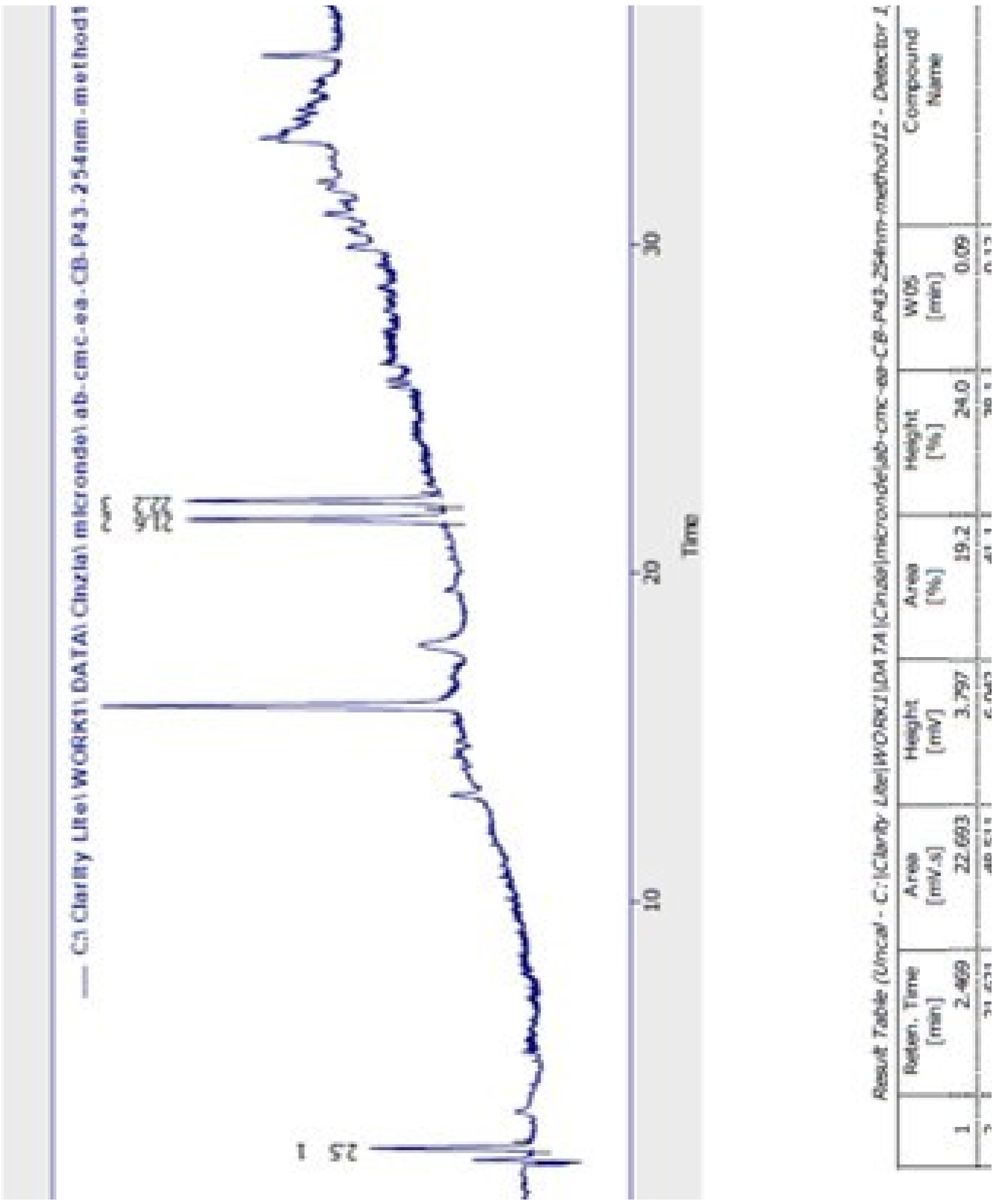

Reaction in Table 2 –entry 3 (conventional heating)

Reaction in Table 2 –entry 3 (microwave heating)

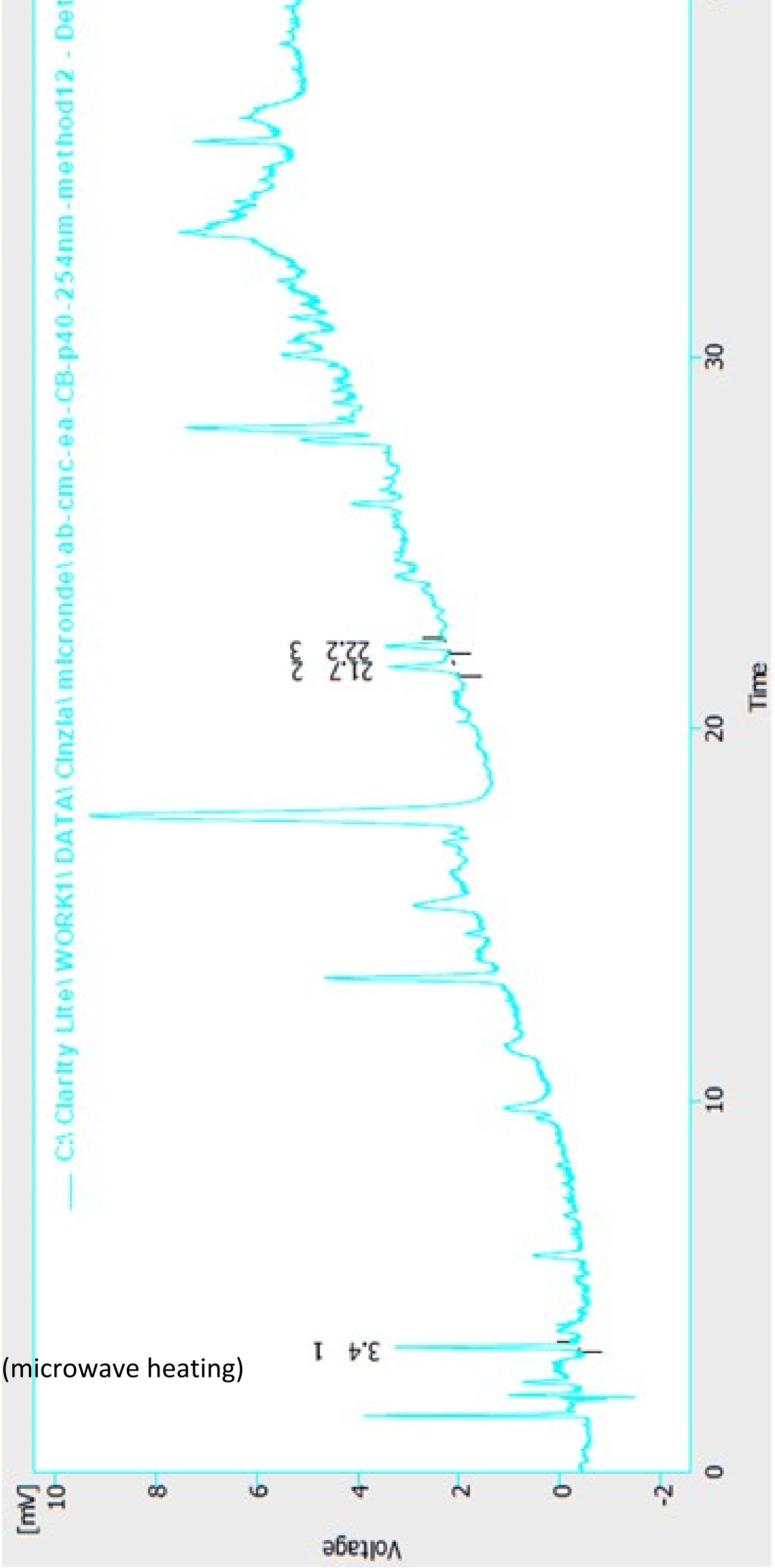

Result Table (Uncal - C:|Clarity Lite|WORK1|DATA|Cinzia|microonde|ab-cmc-ea-CB-p40-254nm-method12 - Detector 1)

	Reten. Time [min]	Area [mV.s]	Height [mV]	Area [%]	Height [%]	W05 [min]	Compound Name
1	3.363	18.469	3.633	44.7	58.8	0.08	
2	21.667	13.315	1.331	32.2	21.5	0.14	
3	22.224	9.533	1.220	23.1	19.7	0.12	
	Total	41.317	6.184	100.0	100.0		

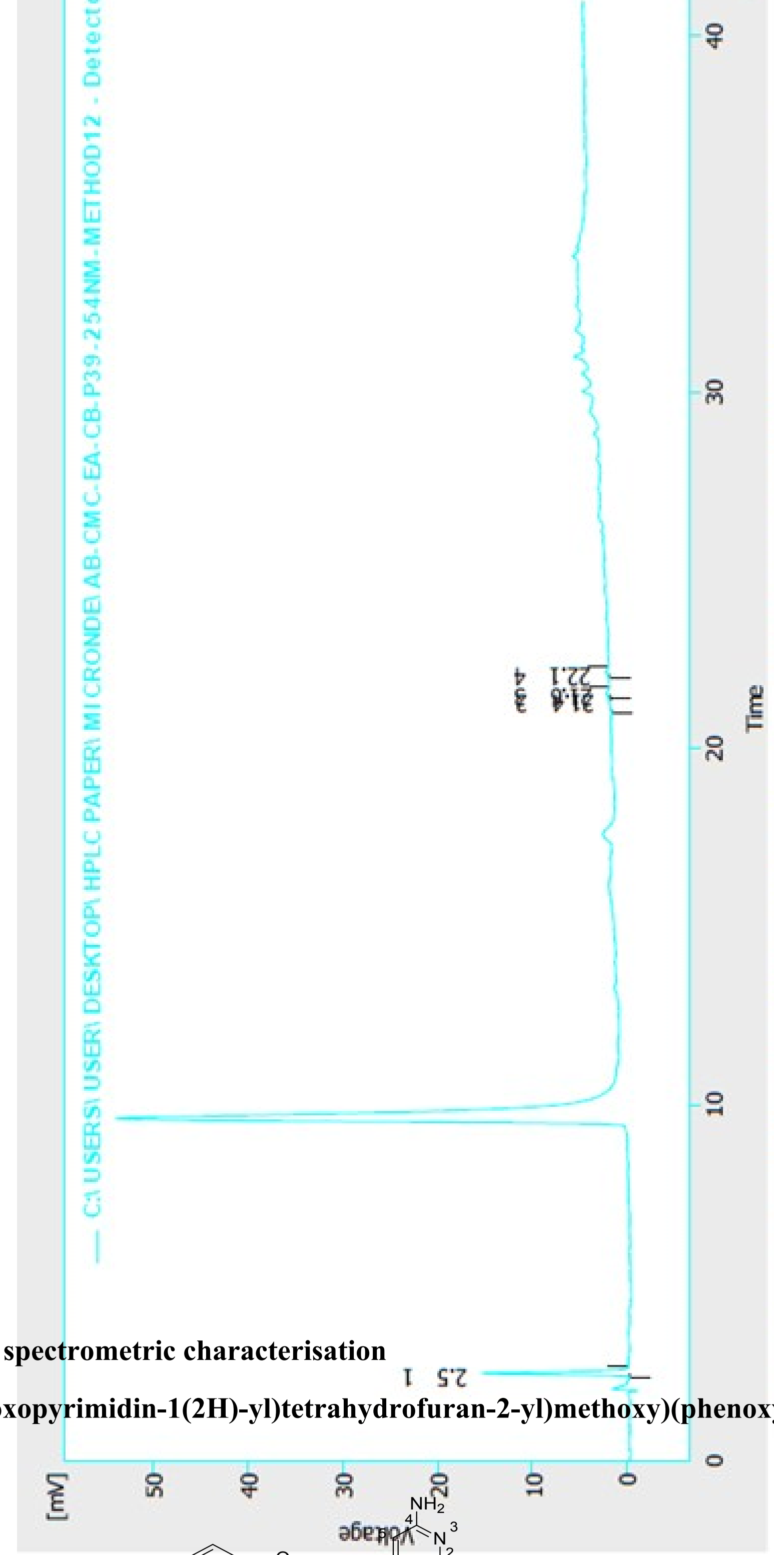

Result Table (Uncal - C:\USERS\USER\DESKTOP\HPLC PAPER\MICRONDE\AB-CMC-EA-CB-P39-254NM-METHOD12 - Detector 1)

	Reten. Time [min]	Area [mV.s]	Height [mV]	Area [%]	Height [%]	W05 [min]	Compound Name
1	2.464	83.468	15.583	89.2	93.5	0.09	
2	21.387	2.939	0.258	3.1	1.5	0.04	
3	21.555	4.318	0.438	4.6	2.6	0.14	
4	22.141	2.802	0.380	3.0	2.3	0.11	
	Total	93.528	16.659	100.0	100.0		

3.2.3 Spectroscopic and spectrometric characterisation

Benzyl(((5-(4-amino-2-oxopyrimidin-1(2H)-yl)tetrahydrofuran-2-yl)methoxy)(phenoxy)phosphoryl)-L-alaninate (11)

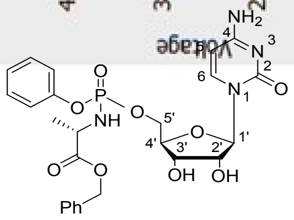

Procedure: standard procedure A and B

State: colourless wax (30 mg)

^{1}H-NMR (500 MHz, CDCl$_3$) δ 8.03 (d, J = 8.8 Hz, 2H, -C_6H_5, -H_5), 7.25 (dt, J = 13.2, 6.1 Hz, 7H, -C_6H_5, -NH_2), 7.12 (dd, J = 16.5, 8.3 Hz, 4H, -C_6H_5), 6.78 (d, J = 8.8 Hz, 2H, -H_6, -$H_{1'}$), 5.17 – 4.90 (m, 2H, -CH_2), 4.18 – 3.87 (m, 2H, -$H_{5'}$), 3.86 – 3.67 (m, 1H, -CH), 1.34 (d, J = 7.0 Hz, 2H, -$H_{3'}$, -$H_{4'}$), 1.30 – 1.24 (m, 1H, -$H_{2'}$), 1.20 (d, J = 15.3 Hz, 3H, -CH_3) ppm.

^{13}C-NMR (126 MHz, $CDCl_3$) δ 129.73 (CH, C-aromatic), 129.70 (CH, C-aromatic), 128.66 (CH, C-aromatic), 128.53 (CH, C-aromatic), 128.24 (CH, C-aromatic), 125.10 (CH, C-aromatic), 120.23 (CH, C-aromatic), 67.32 (CH_2, C-aliphatic, -$C_{5'}$), 67.22 (CH_2, C-aliphatic), 50.52 (CH, C-aliphatic), 29.71 (CH, C-aliphatic, -$C_{3'}$), 21.08 (CH_3, C-aliphatic) ppm.

^{31}P-NMR (202 MHz, $CDCl_3$) δ 3.49, 3.47 ppm.

MS(ES)$^+$ m/z 561.21 $[M+H]^+$

3.3 2',3'-Dideoxycytidine

3.3.1 Conditions for the optimisation study

Table 3. 2′, 3′-Dideoxycytidine phosphoramidate and by-products. *Reagent and conditions*: a) *t*-BuMgCl (3 equivalents), solvent; b) NMI, solvent. In blue and bold, best conditions presented in the main paper.

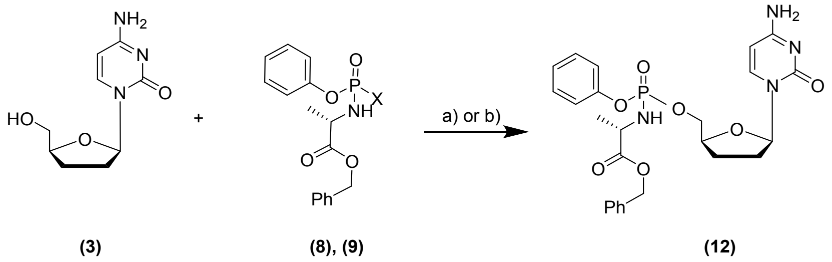

(8): X = -pNO$_2$Ph *reagents and condition* a)
(9): X = -Cl *reagents and condition* b)

5'-O-phosphoramidate

	Conventional heating (55 °C)	Microwave irradiation

	entry	Reagents	Solvent	Time (min)	Yield* (%)		Hold time (min)	T (°C)	Yield* (%)	
					(3)	(12)			(3)	(12)
**Grignard method, (a)**	**1***	***t*-BuMgCl (3 Eq) + (8)**	**DMF**	**135**	**1**	**99**	30	65	30	70
	2	*t*-BuMgCl (3 Eq) + (**8**)	DMF	-	-	-	**5**	**75**	**19**	**81**
**NMI method, (b)**	**3****	**NMI** + (**9**)	**THF**	**300**	**57**	**43**	**10**	**65**	**3**	**97**

* Conversion of the parent nucleoside into the desired 5'-protide was calculated on UPLC analysis (method 2).
** Conversion of the parent nucleoside into the desired 5'-protide was calculated on UPLC analysis (method 1).

3.3.2 UPLC spectra

2’, 3’-dideoxycytidine (**3**) – HPLC purity

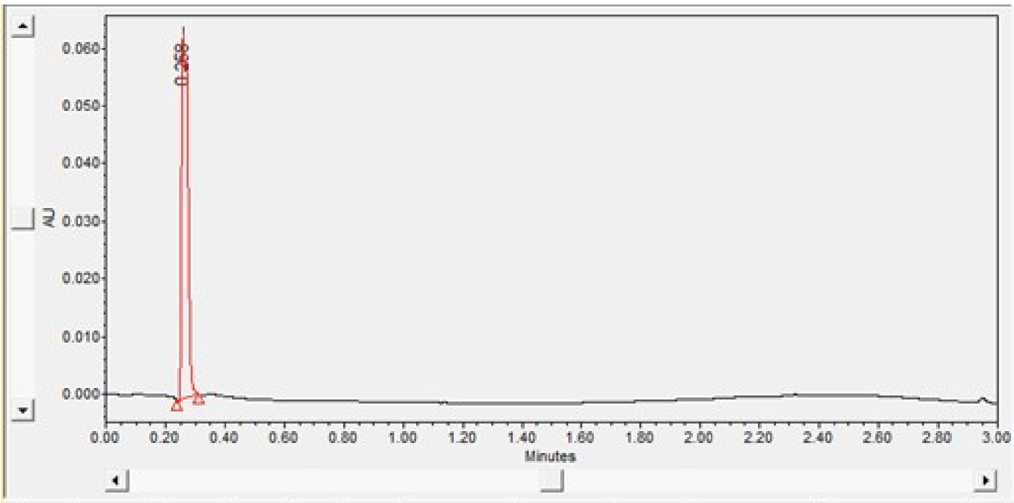

	Name	Retention Time (min)	Area (µV*sec)	% Area	Height (µV)	Int Type	Peak Type	Peak Codes	ARTsec	Aceto_Theoretical	Caffeine_Theoretical	Mass
1		0.258	96363	100.00	63316	bb	Unknown		15.491683			

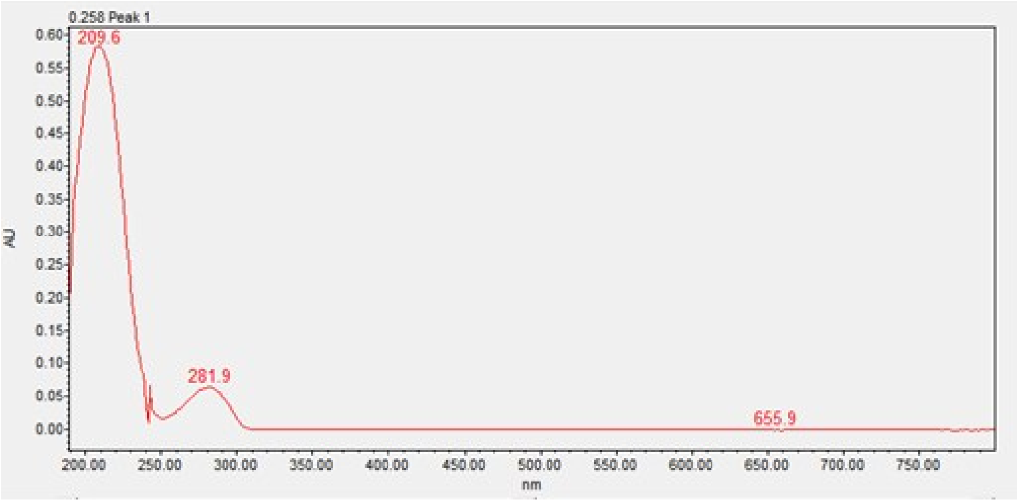

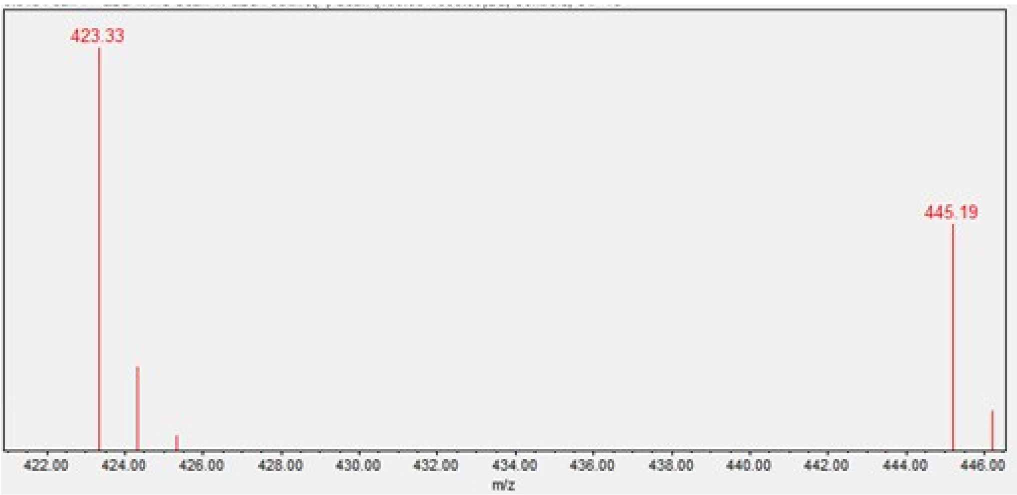

Reaction in Table 3 – entry 3 (conventional heating- standard procedure)

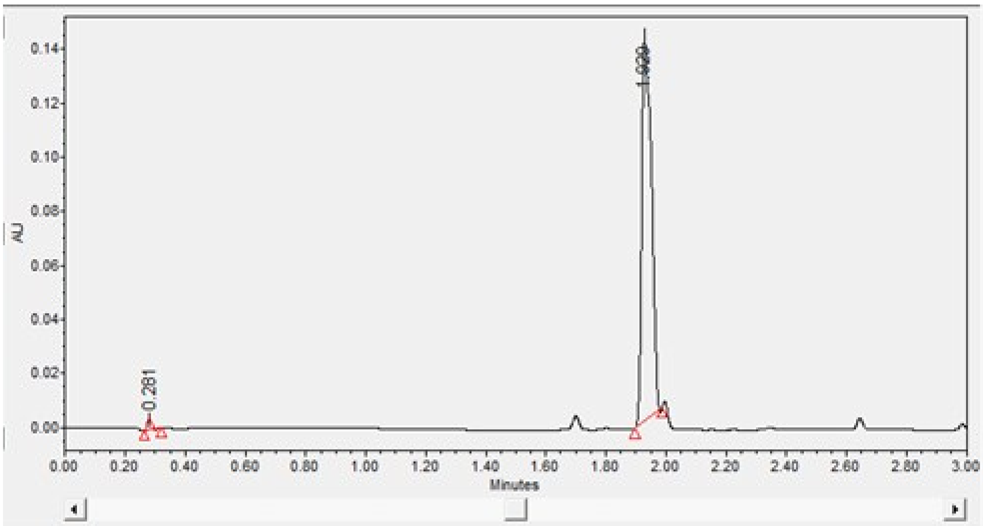

lame	Retention Time (min)	Area (µV*sec)	% Area	Height (µV)	Int Type	Peak Type	Peak Codes	ARTsec	Aceto_Theoretical	Caffeine_Theoretical	Mass_
	0.281	4507	1.40	3796	bb	Unknown		16.874484			
	1.929	316267	98.60	142429	bb	Unknown		115.744658			

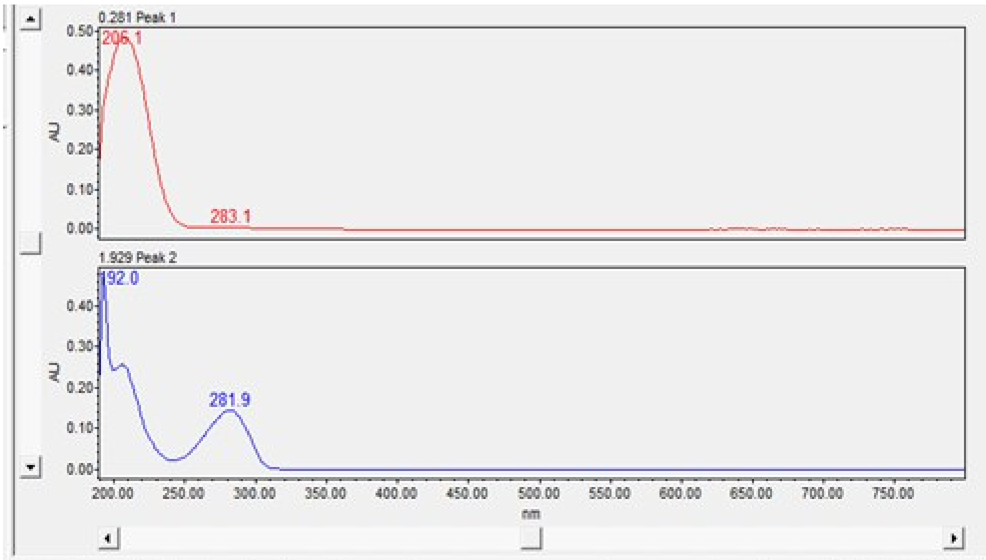

	Image	Select	Retention Time (min)	Info	Max. Absorbance (au or eu)	Baseline Correction	Smoothing Level	Deriv. Level	Smoothing Type	Spline	Resolution (nm)	Start Wavelength (nm)	End Waveleng (nm)
1		☑	0.281	Peak 1	0.48490	☑	None	None	None	☐	1.2	189.7	799
2		☑	1.929	Peak 2	0.47123	☑	None	None	None	☐	1.2	189.7	799

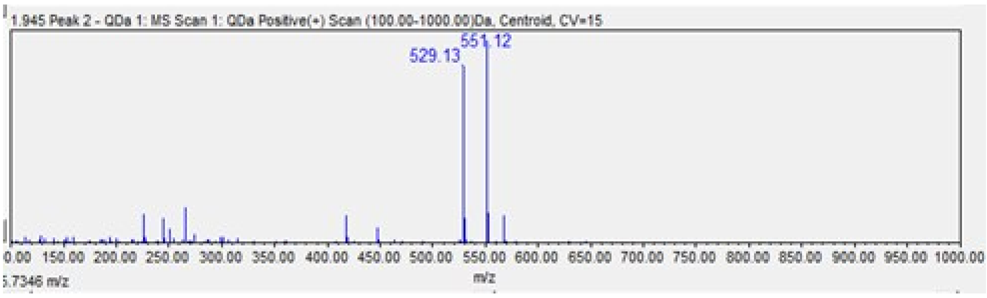

Reaction in Table 3 – entry 3 (microwave irradiation)

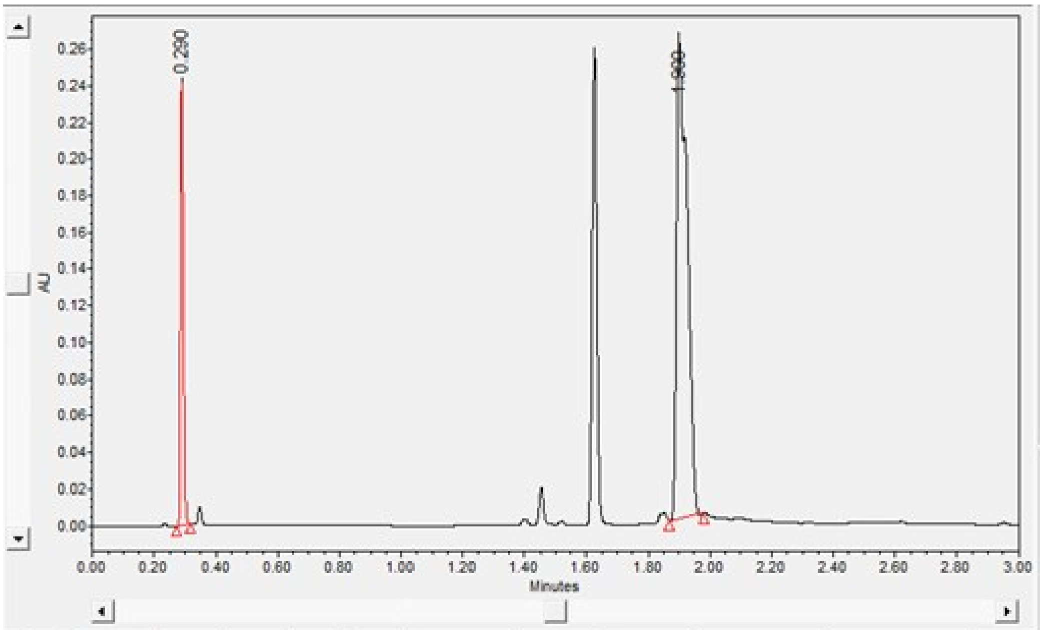

	Name	Retention Time (min)	Area (µV*sec)	% Area	Height (µV)	Int Type	Peak Type	Peak Codes	ARTsec	Aceto_Theoretical	Caffeine_Theoretical	Mass_E
1		0.290	156527	19.62	242167	bb	Unknown		17.374051			
2		1.900	641428	80.38	260887	bb	Unknown		114.022365			

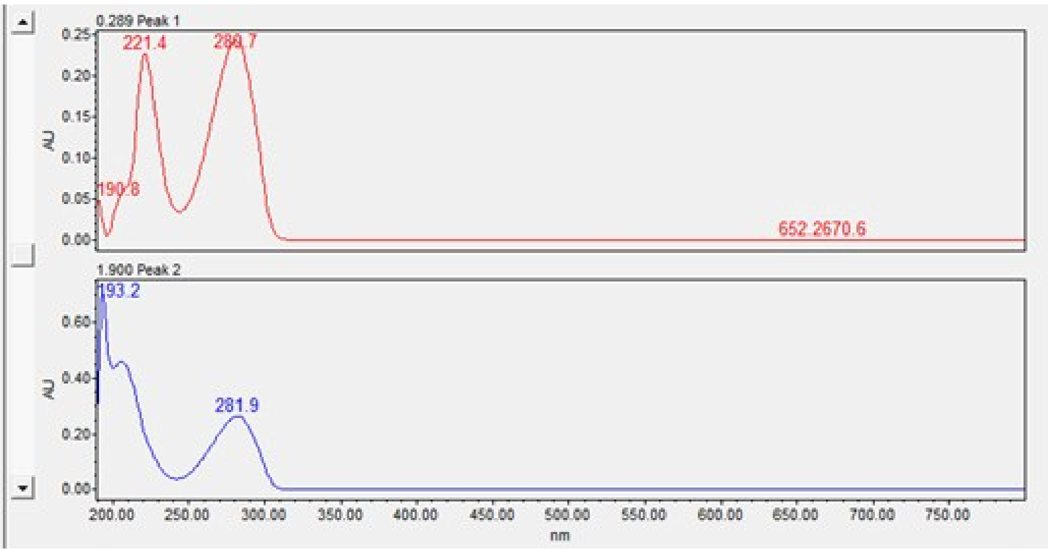

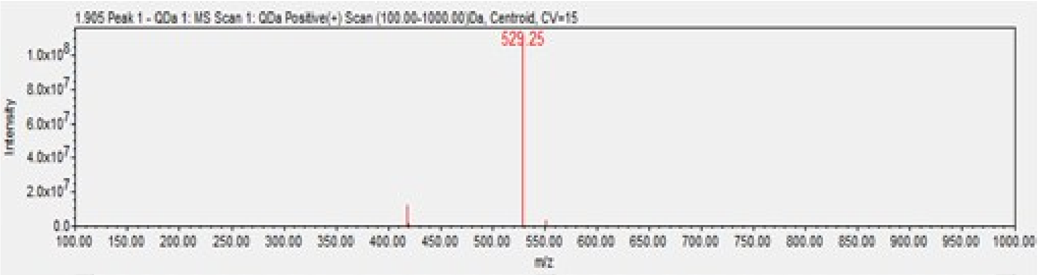

Reaction in Table 3 – entry 4 (conventional heating)

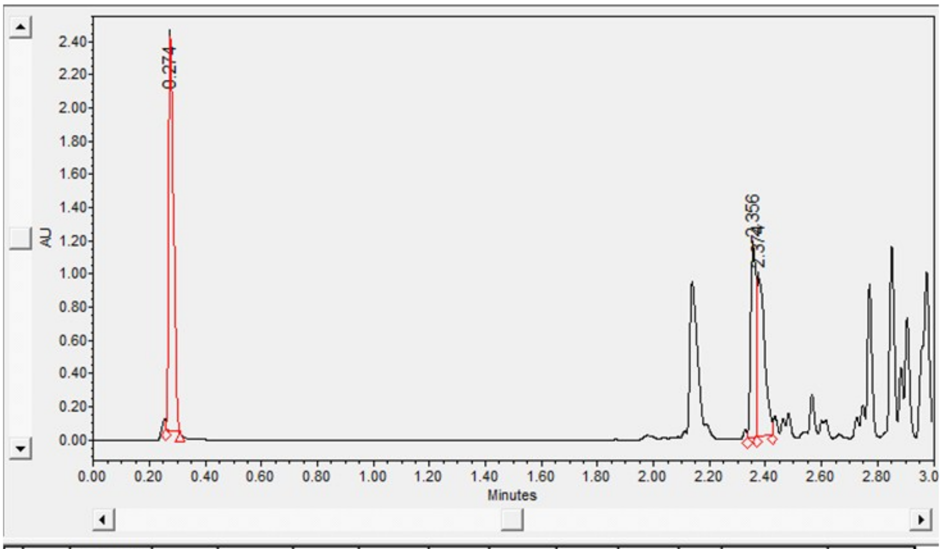

	Name	Retention Time (min)	Purity1 Angle	Purity1 Threshold	Purity1 Flag	Area (µV*sec)	% Area	Height (µV)	Int Type	Amount	Units	Peak Type	Peak Codes
1		0.274	54.940	49.472	☑	2913372	46.83	2373067	Vb			Unknown	
2		2.356	27.313	52.891	☐	1562054	25.11	1144661	VV			Unknown	
3		2.374	16.544	90.000	☐	1746270	28.07	953189	VV			Unknown	

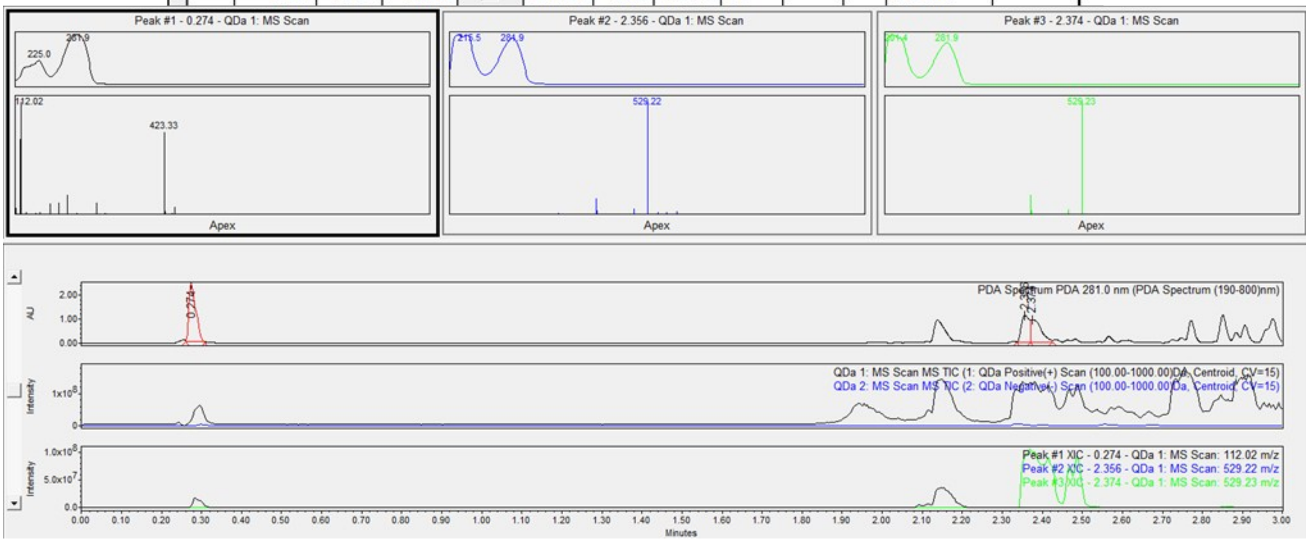

Reaction in Table 3 – entry 4 (microwave heating)

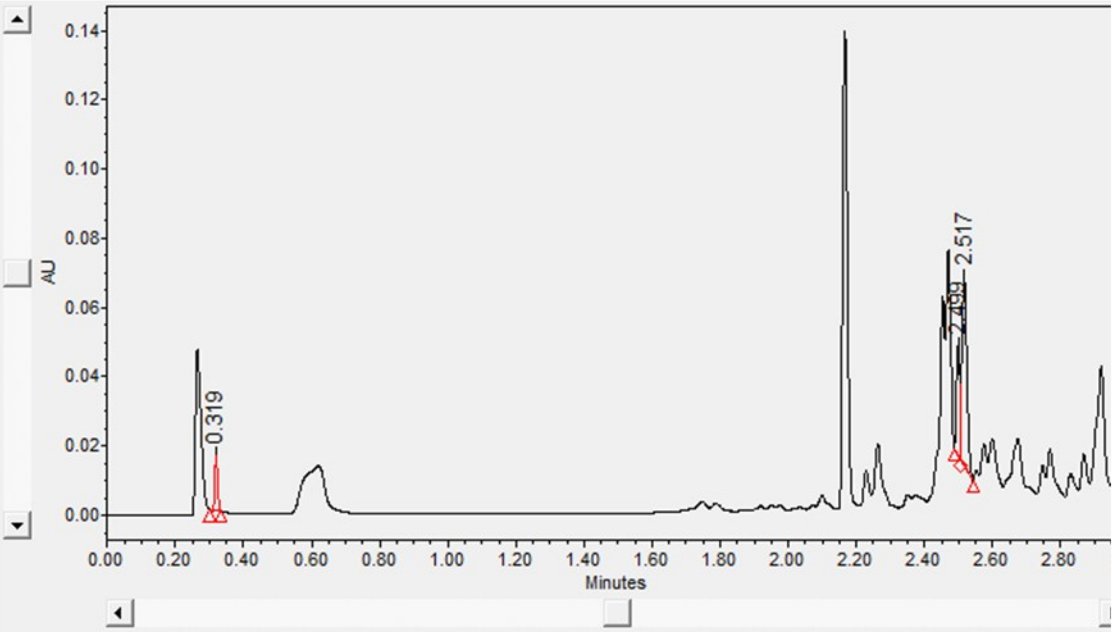

	Name	Retention Time (min)	Purity1 Angle	Purity1 Threshold	Purity1 Flag	Area (µV*sec)	% Area	Height (µV)	Int Type	Amount	Units	Peak Type	Peak Codes
1		0.319	11.813	28.060	☐	9083	10.43	15899	bb			Unknown	
2		2.499	4.397	44.813	☐	23036	26.46	31720	bV			Unknown	
3		2.517	16.464	30.987	☐	54948	63.11	54345	Vb			Unknown	

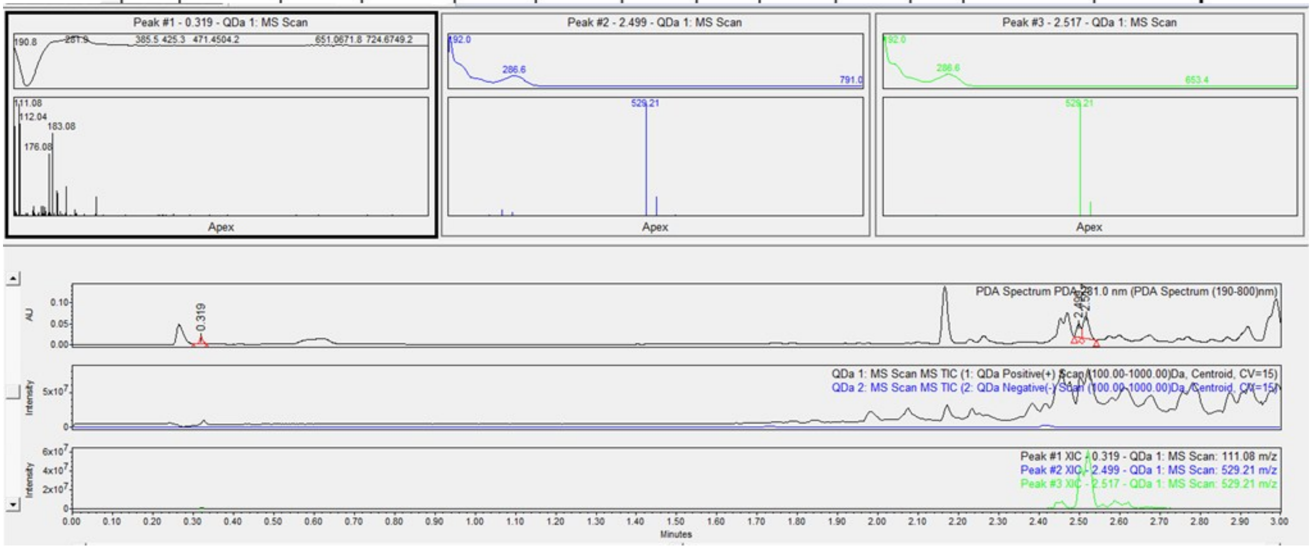

3.3.3 Spectroscopic and spectrometric characterisation

2', 3'-Dideoxycytidine-5'-O[phenyl-(benzyloxy-L-alaninyl)] phosphate (12)

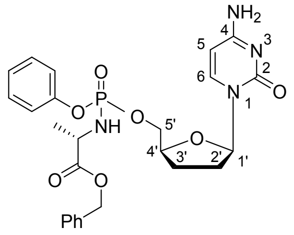

Procedure: standard procedure A and B

State: colourless wax (25 mg)

^{1}H-NMR (500 MHz, CDCl$_3$) δ 7.64 (d, J = 7.4 Hz, 1H, -H_6), 7.30 – 7.20 (m, 7H, -C_6H_5), 7.15 – 7.04 (m, 3H, -C_6H_5), 5.95 (m, 1H, -$H_{1'}$), 5.62 (dd, J =7.4 Hz, 1H, -H_6), 5.10 – 5.00 (m, 2H, -CH_2), 4.27 (m, 1H, -$H_{4'}$), 4.19 – 4.03 (m, 2H, -$H_{5'}$), 3.97 (m, 2H, -CH, -NH), 2.32 (td, J = 13.7, 7.3 Hz, 1H, -$H_{2'}$), 1.94 – 1.77 (m, 2H, -$H_{2'}$, -$H_{3'}$), 1.75 – 1.60 (m, 1H, , -$H_{3'}$), 1.31 (dd, J = 12.7, 7.6 Hz, 3H, -CH_3) ppm.

^{13}C-NMR (126 MHz, CDCl$_3$) δ 173.65 (t, J = 7.56 Hz, C, C=O), 165.49 (C, C-aromatic), 155.74 (C, C-aromatic), 150.65 (t, J = 6.3 Hz, C, C-aromatic), 140.95 (CH, C-aromatic, C_6), 135.34 (C, C-aromatic), 129.75 (CH, C-aromatic), 128.68 (CH, C-aromatic), 128.58 (CH, C-aromatic), 128.49 (CH, C-aromatic), 126.09 (CH, C-aromatic), 125.12 (CH, C-aromatic), 120.16 (CH, C-aromatic), 120.05 (C, C-aromatic), 115.99 (C, C-aromatic), 94.05 (CH, C-aromatic, C_5), 87.51 (CH, C-aliphatic, $C_{1'}$), 79.43 (CH_2, C-aliphatic, $C_{4'}$), 67.36 (CH_2, C-aliphatic, $C_{5'}$), 67.20 (CH_2, C-aliphatic), 50.50 (CH, C-aliphatic), 32.71 (CH_2, C-aliphatic, $C_{2'}$), 25.39 (CH_2, C-aliphatic, $C_{3'}$), 20.76 (CH_3, C-aliphatic) ppm.

^{31}P-NMR (202 MHz, CDCl$_3$) δ 2.81, 2.71 ppm.

MS(ES)$^+$ m/z 529.24 $[M+H]^+$, 527.2 $[M-H]^-$

3.4 Guanosine

3.4.1 Conditions for the optimisation study

Table 4. Guanosine phosphoramidate and by side products. *Reagent and conditions*: a) *t*-BuMgCl (3 equivalents), solvent; b) NMI (6.3 equivalents), solvent. In blue and bold, best conditions presented in the main paper.

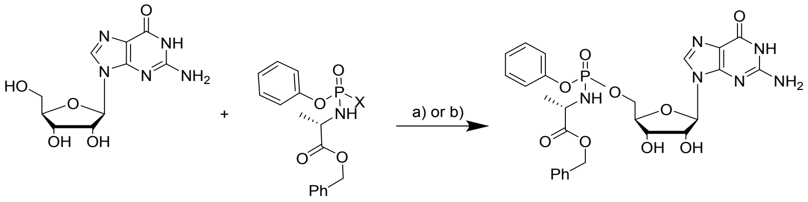

(4) **(8), (9)** **(13)**

(8): X = -pNO$_2$Ph *reagents and condition* a)
(9): X = -Cl *reagents and condition* b)

5'-O-phosphoramidate

				Conventional heating (55 °C)			Microwave irradiation			
	entry	Reagents	Solvent	Time (min)	Yield* (%) (4)	Yield* (%) (13)	Hold time (min)	T (°C)	Yield* (%) (4)	Yield* (%) (13)
Grignard method, (a)	1	*t*-BuMgCl (3 Eq) + (**8**)	THF/NMP	300	100		5	65	83	17
	2						20	65	100	-
	**3***	***t*-BuMgCl (3 Eq) + (8)**	**DMF**	**450**	**34**	**66**	60	65	54	46
	4	*t*-BuMgCl (3 Eq) + (**8**)	DMF				25	65	33	67
	5	*t*-BuMgCl (3 Eq) + (**8**)	DMF				30	65	18	82
	6**	*t*-BuMgCl (3 Eq) + (**8**)	DMF				**10**	**75**	**12**	**88**
	7	*t*-BuMgCl (3 Eq) + (**8**)	DMF				40	75	60	40
	8	*t*-BuMgCl (3 Eq) + (**8**)	DMF				5	85	66	34
	9	*t*-BuMgCl (3 Eq) + (**8**)	DMF				25	85	77	23
NMI method, (b)	10	NMI + (**9**)	THF/pyridine	120	100	-	10	65	89	11
	11*	**NMI + (9)**	**THF/pyridine**	**1440**	**51**	**49**	**35**	**65**	**87**	**13**
	12	NMI + (**9**)	THF/pyridine				35	75	100	-
	13	NMI + (**9**)	THF/pyridine				25	85	100	-
	14	*t*-BuMgCl (3 Eq) + (**9**)	THF	240	100	-	30	65	98	2

*Conversion of the parent nucleoside into the desired 5'-protide was calculated on HPLC analysis (method 1).
**Conversion of the parent nucleoside into the desired 5'-protide was calculated on UPLC analysis (method 1).

3.4.2 HPLC spectra

Guanosine (**4**) -

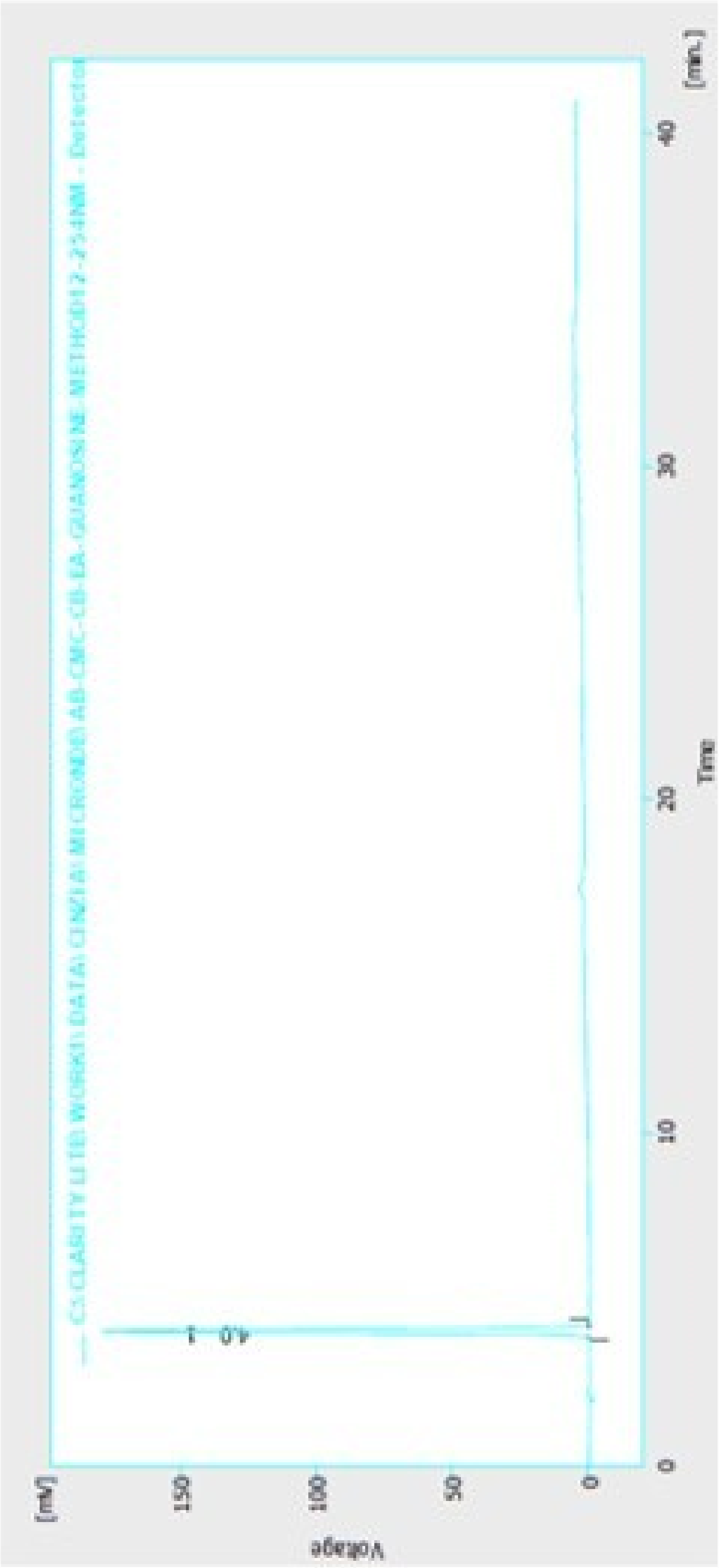

Result Table (Uncal - C:\CLARITY LITE\WORK1\DATA\CINZIA\MICRONDE\AB-CMC-CB-EA-GUANOSINE-METHOD12-254NM - Detector 1)

	Reten. Time [min]	Area [mV.s]	Height [mV]	Area [%]	Height [%]	W05 [min]	Compound Name
1	4.043	1462.755	179.399	100.0	100.0	0.13	
	Total	1462.755	179.399	100.0	100.0		

Compound (**13**) – HPLC purit

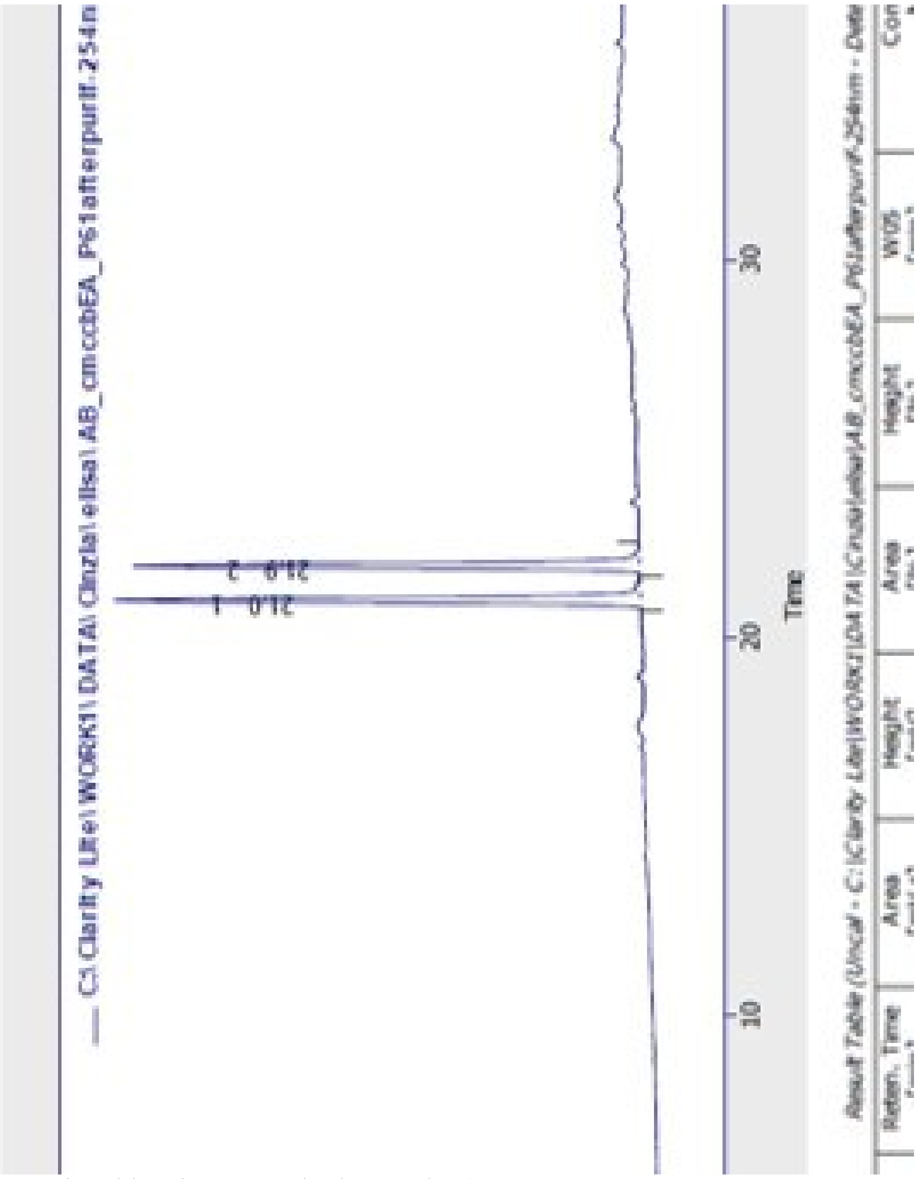

Reaction in Table 4 – entry 3 (conventional heating – standard procedure)

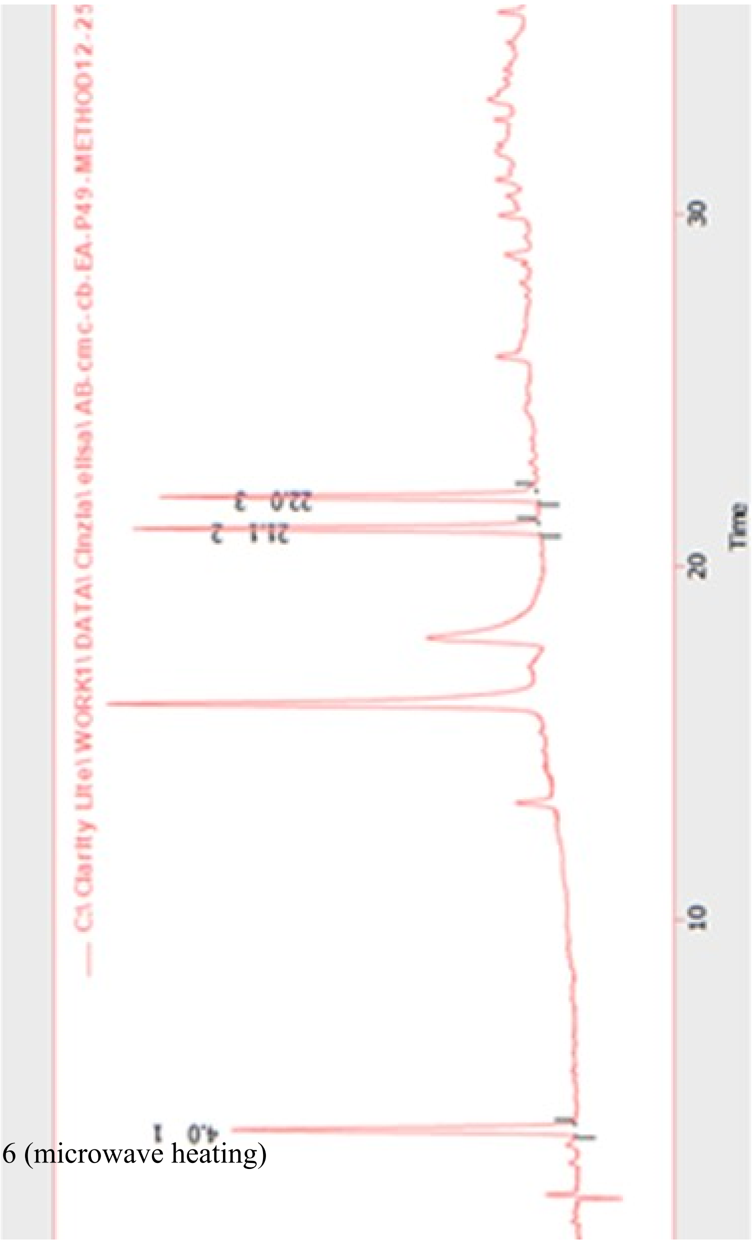

Result Table (Uncal - C:\Clarity Lite\WORK1\DATA\Cinzia\elisa\AB-cmc-cb-EA-P49-METHOD12-254NM - Detecto

	Reten. Time [min]	Area [mV.s]	Height [mV]	Area [%]	Height [%]	W05 [min]	Compour Name
1	4.024	218.283	23.358	32.5	30.5	0.15	
2	21.061	239.891	27.634	35.7	36.0	0.14	
3	21.965	214.209	25.694	31.9	33.5	0.13	

Reaction in Table 4 – entry 6 (microwave heating)

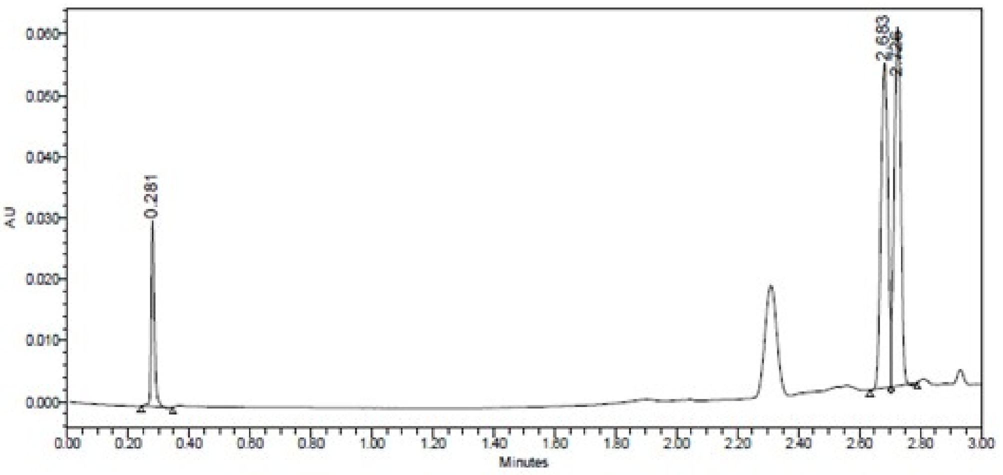

Processed Channel: PDA Spectrum
PDA 254.0 nm (PDA Spectrum (190-800)nm)

	Retention Time (min)	Area	% Area	Height	Purity1 Flag
1	0.281	23566	11.90	30339	No
2	2.683	85946	43.40	52999	No
3	2.726	88500	44.69	58433	No

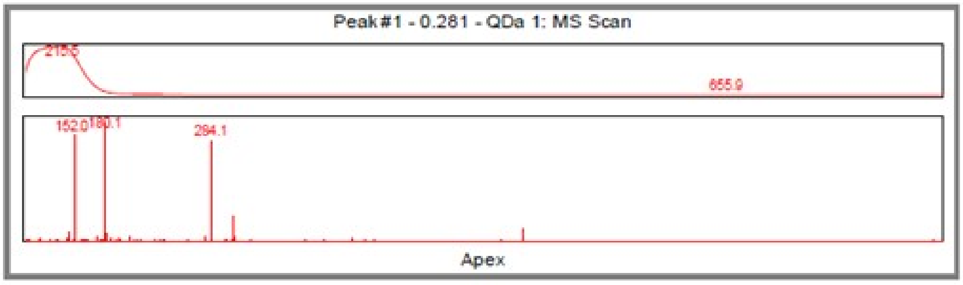

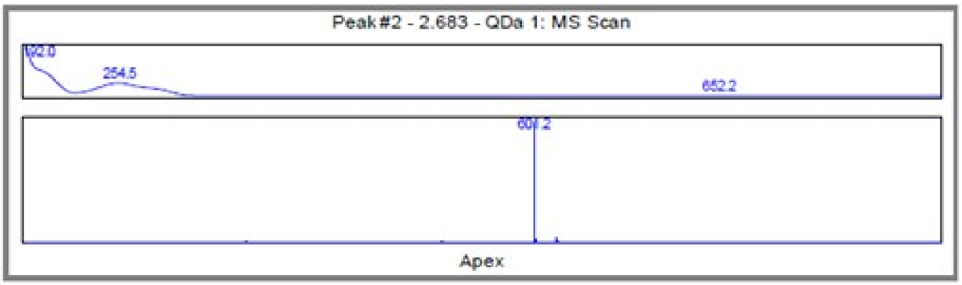

Reaction in Table 4 – entry 11 (conventional heating)

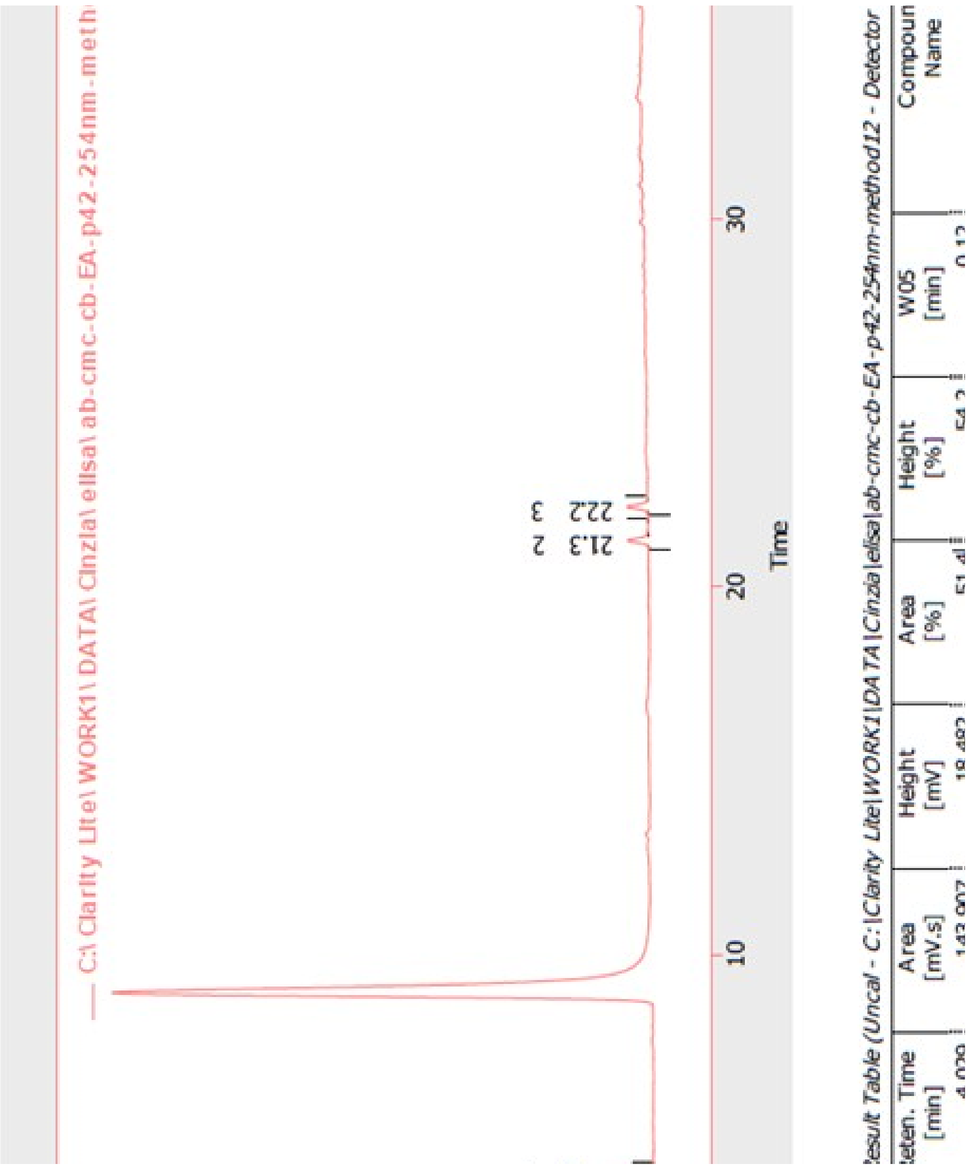

Reaction in Table 4 – entry 11 (microwave heating)

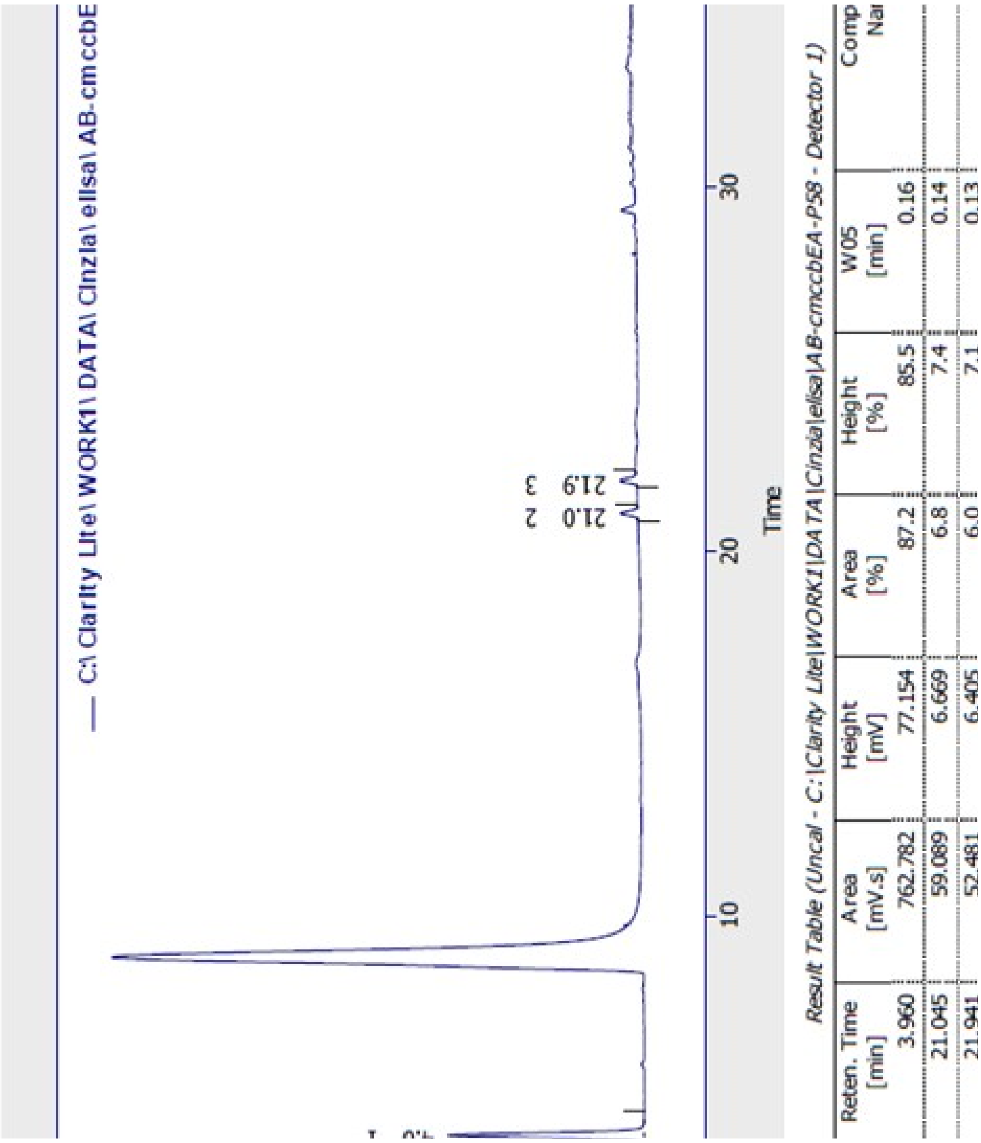

Guanosine-5’-O[phenyl-(benzyloxy-L-alaninyl)] phosphate (13)

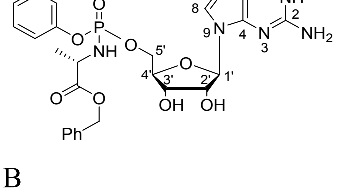

Procedure: standard procedure A and B

State: pale brown wax (30 mg)

^{1}H-NMR (500 MHz, CD_3OD) δ 7.88 (d, J = 9.0 Hz, 1H), 7.38 – 7.26 (m, 7H,-C_6H_5), 7.26 – 7.09 (m, 4H, -C_6H_5), 7.00 (t, J = 7.1 Hz, 1H, -NH), 5.88 (dd, J = 9.7, 5.2 Hz, 1H, - $H_{1'}$), 5.17 – 5.04 (m, 2H, -CH_2), 4.61 (dt, J = 10.0, 5.1 Hz, 1H, -$H_{2'}$), 4.42 – 4.25 (m, 3H, -$H_{4'}$, –$H_{5'}$), 4.21 (s, 1H, -$H_{3'}$), 4.05 – 3.93 (m, 1H, -

CH), 1.39 – 1.25 (m, 3H, -CH_3) ppm.

^{13}C-NMR (126 MHz, $CDCl_3$) δ 173.49 (C, C-aromatic), 158.00 (C, C-aromatic), 153.90 (C, C-aromatic), 153.18 (C, C-aromatic), 150.67 (C, C-aromatic), 135.80 (C, C-aromatic), 129.34, 129.18 (CH, C-aromatic), 128.71, 128.62 (CH, C-aromatic), 128.29 (CH, C-aromatic), 126.15 (CH, C-aromatic), 124.34 (CH, C-aromatic), 123.58 (CH, C-aromatic), 120.96, 120.89 (CH, C-aromatic), 115.69 (CH, C-aromatic), 114.23, 114.19 (CH, C-aromatic), 111.29 (CH, C-aromatic), 88.40 (CH, C-aliphatic, -$C_{1'}$), 82.89 (CH, C-aliphatic, -$C_{3'}$), 73.75 (CH, C-aliphatic, -$C_{4'}$), 70.28 (CH, C-aliphatic, -$C_{2'}$), 67.47 (CH_2, C-aliphatic, -$C_{5'}$), 65.44 (CH_2, C-aliphatic), 50.50 (CH, C-aliphatic), 19.01 (CH_3, C-aliphatic) ppm.

^{31}P-NMR (202 MHz, $CDCl_3$) δ 4.07, 3.77 ppm.

MS(ES)$^+$ m/z 601.3 [M+ H]$^+$

3.4.3 Spectroscopic and spectrometric characterisation

3.5 Uridine

3.5.1 Conditions for the optimisation study

Table 5. Uridine phosphoramidate and by-side products. *Reagent and conditions*: a) *t*-BuMgCl (3 equivalents), solvent; b) NMI (6.3 equivalents), solvent. In blue and bold, best conditions presented in the main paper.

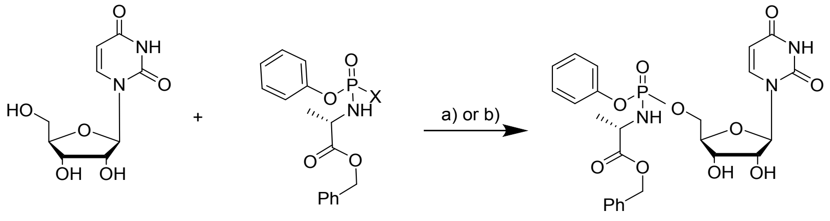

(5) **(8), (9)** **(14)**

(8): X = -pNO$_2$Ph *reagents and condition* a)
(9): X = -Cl *reagents and condition* b)

5'-O-phosphoramidate

				**Conventional heating (55 °C)**			**Microwave irradiation**			
	**entry**	**Reagents**	**Solvent**	**Time (min)**	**Yield* (%) (5)**	**Yield* (%) (14)**	**Hold time (min)**	**T (°C)**	**Yield* (%) (5)**	**Yield* (%) (14)**
**Grignard method, (a)**	**1***	***t*-BuMgCl (3 Eq) + (8)**	**THF/NMP**	**320**	**62**	**38**	**15**	**65**	**62**	**38**
	2**	*t*-BuMgCl (3 Eq) + (**8**)	DMF	180	83	17	30	65	70	30
**NMI method, (b)**	**3***	**NMI + (9)**	**THF**	**350**	**35**	**65**	**35**	**65**	**46**	**54**
	4	NMI + (**9**)	THF				15	75	67	33
	5	NMI + (**9**)	THF				15	85	67	33

* Conversion of the parent nucleoside into the desired 5'-protide was calculated on HPLC analysis (method 1).

**Conversion of the parent nucleoside into the desired 5'-protide was calculated on UPLC analysis (method 1).

3.5.2 HPLC spectra

Uridine (**5**) – HPLC purity

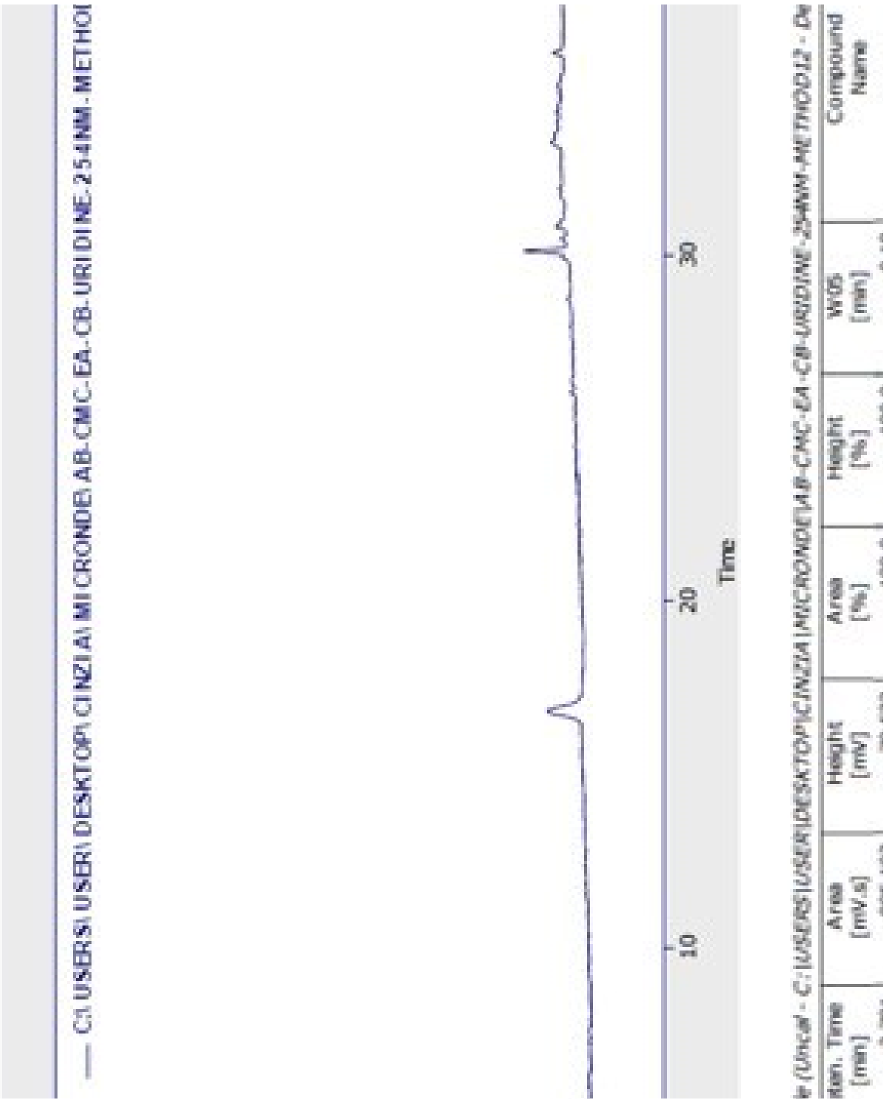

Compound (**14**) – HPLC purit

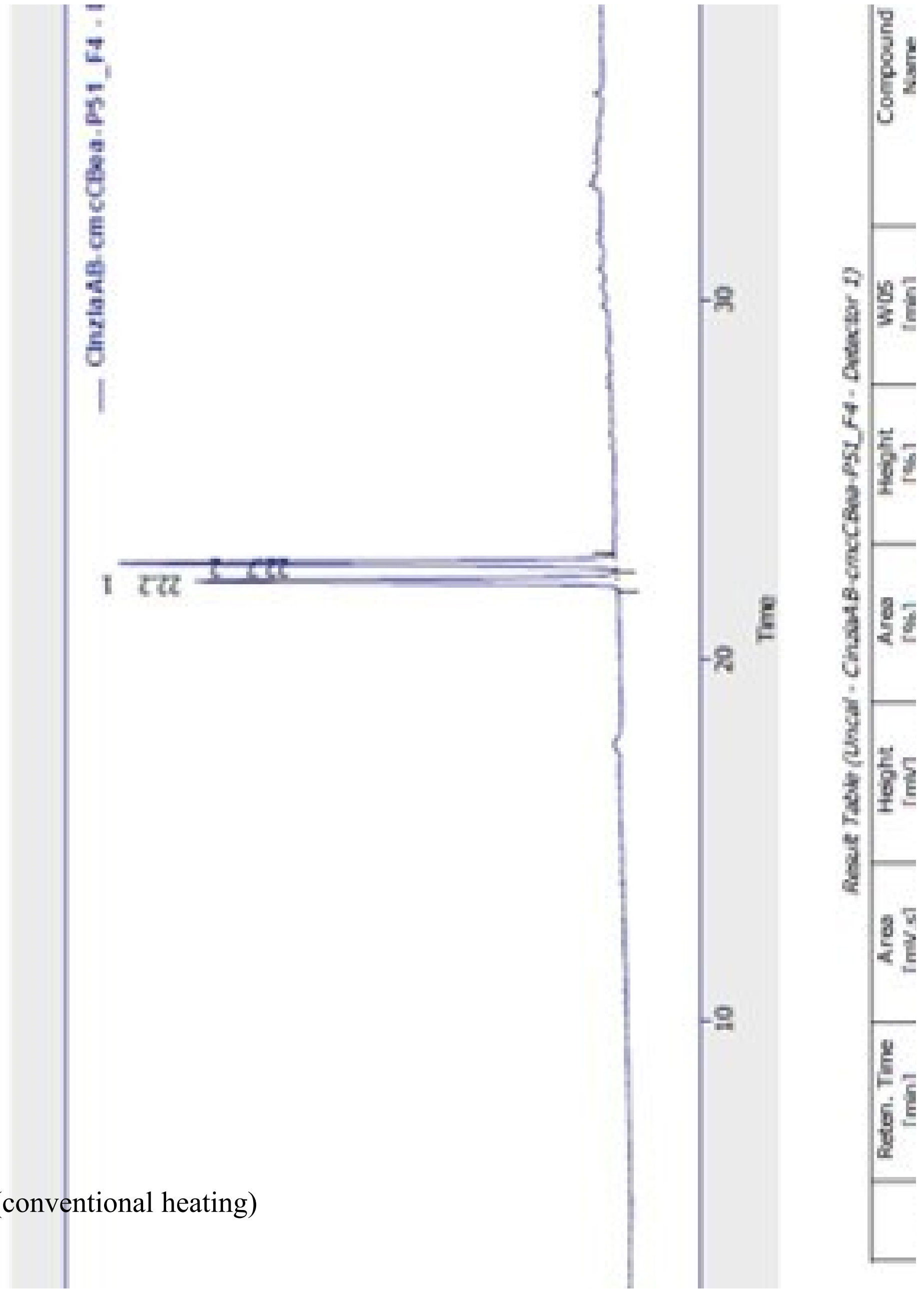

Reaction in Table 5 – entry 1 (conventional heating)

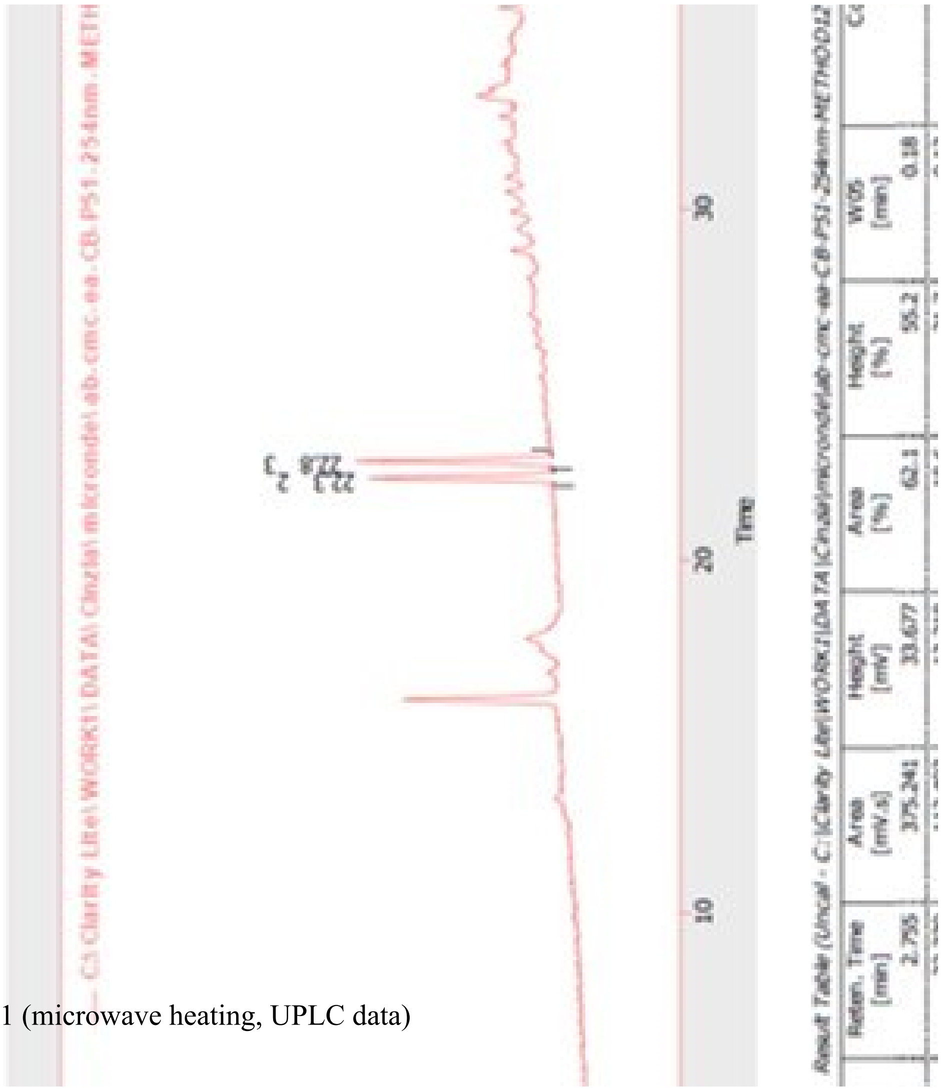

Reaction in Table 5 – entry 1 (microwave heating, UPLC data)

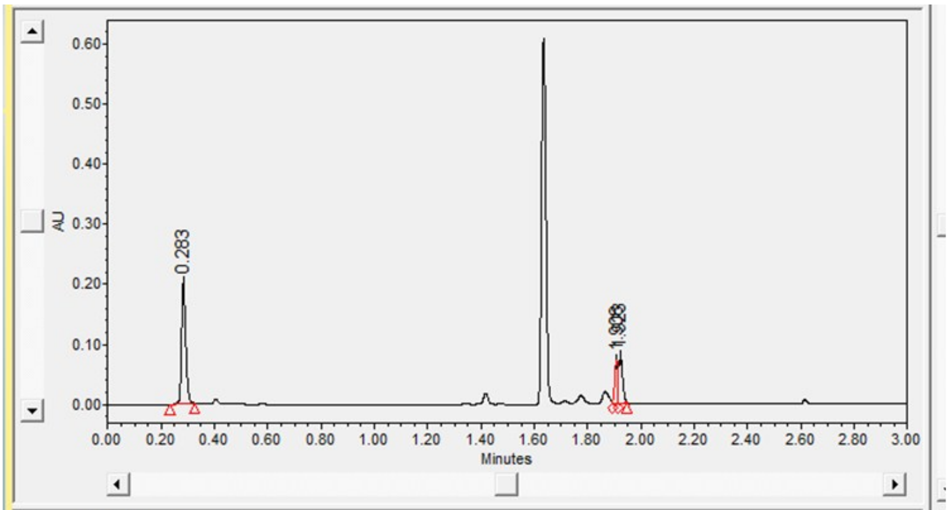

	Name	Retention Time (min)	Purity1 Angle	Purity1 Threshold	Purity1 Flag	Area (µV*sec)	% Area	Height (µV)	Int Type	Amount	Units	Peak Type	Peak Co
1		0.283	40.109	90.000	☐	219070	61.47	200737	bb			Unknown	
2		1.908	25.087	33.709	☐	59124	16.59	70924	VV			Unknown	
3		1.923	45.566	90.000	☐	78191	21.94	75992	Vb			Unknown	

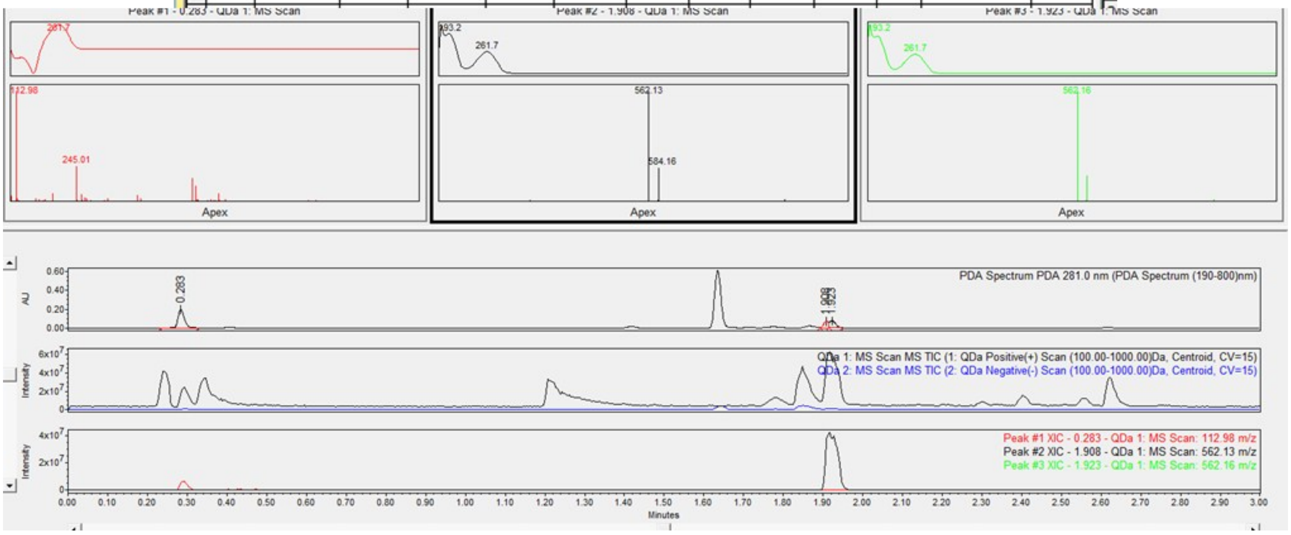

Reaction in Table 5 - entry 3 (conventional heating)

Reaction in Table 5 - entry 3 (microwave heating)

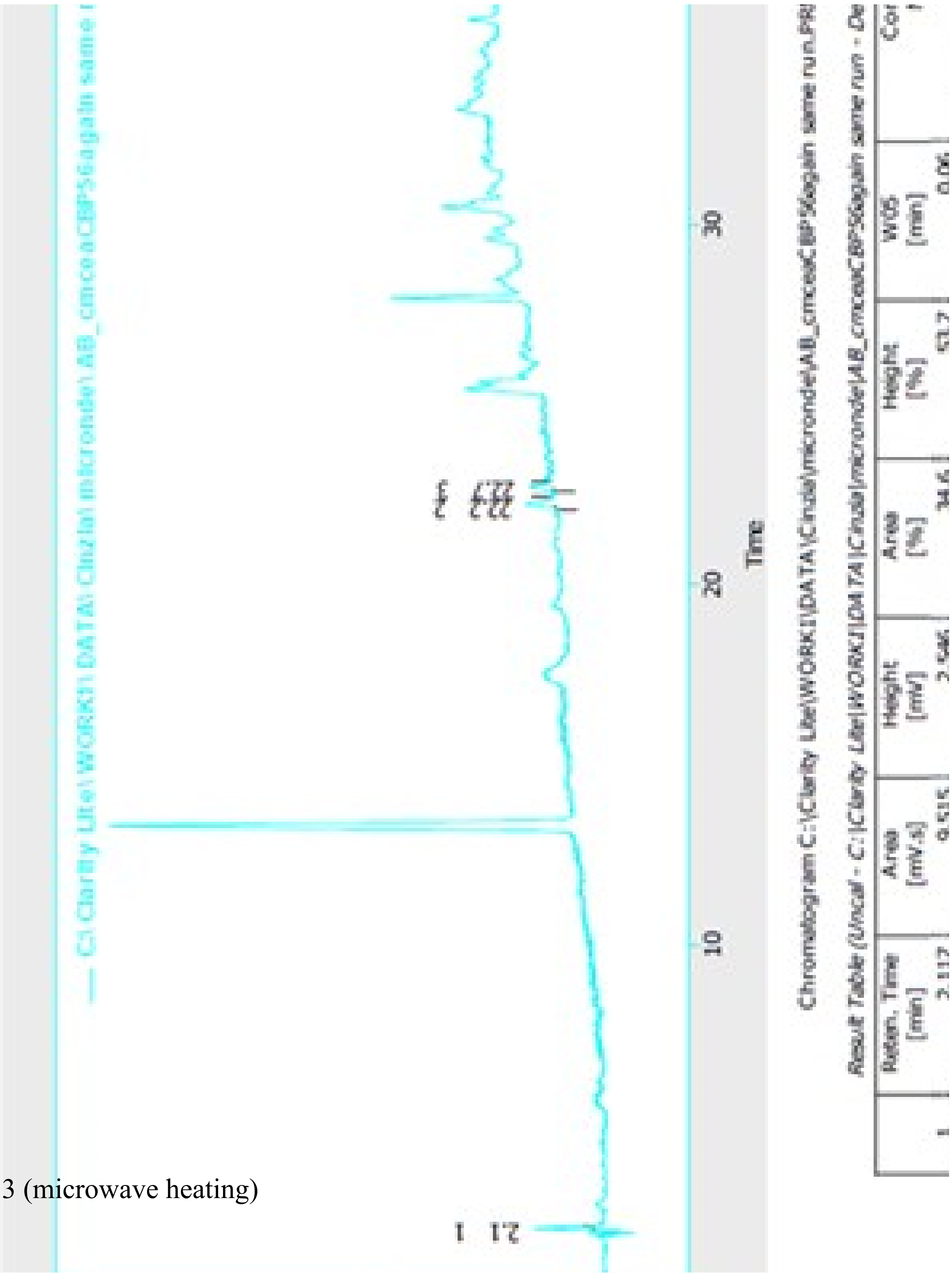

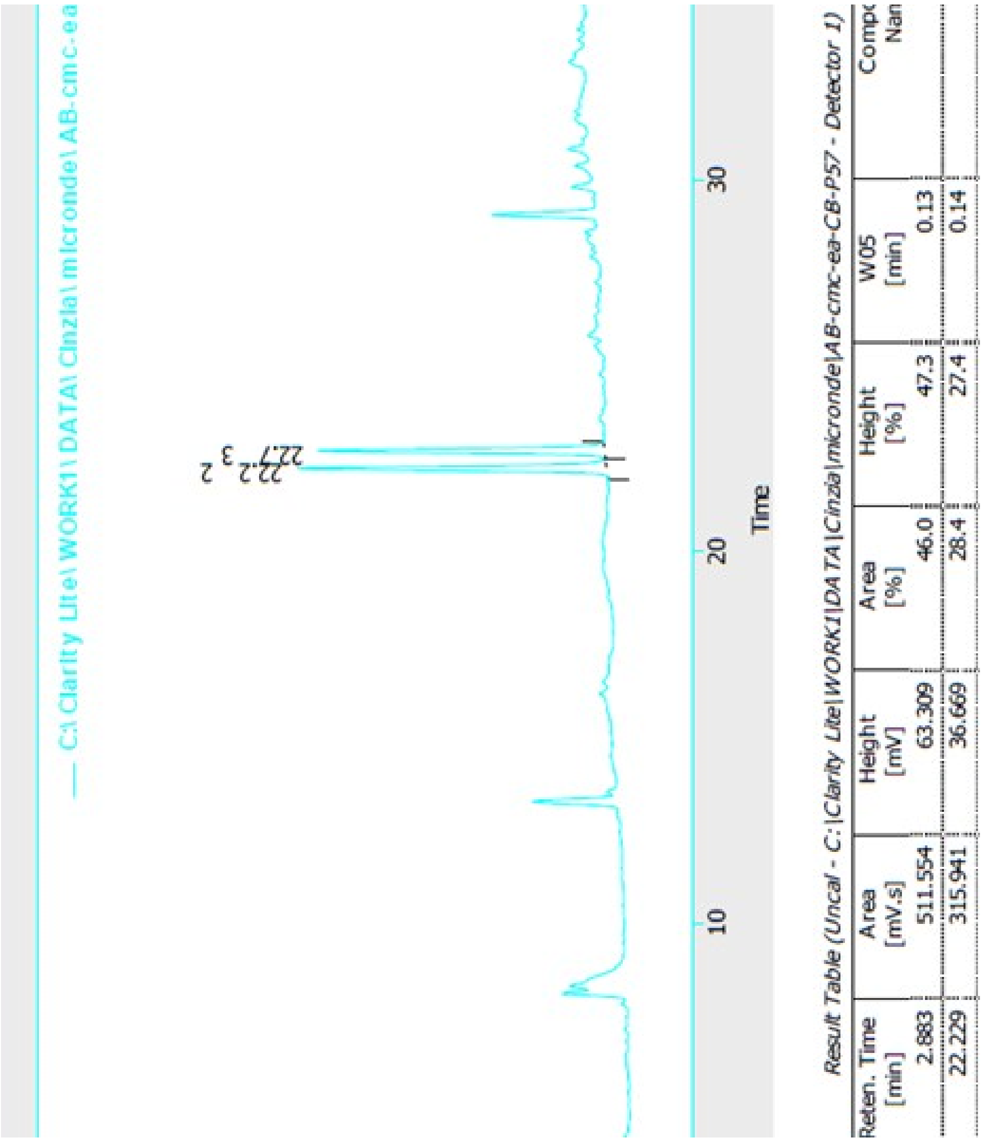

3.5.3 Spectroscopic and spectrometric characterisation

Uridine-5'-O[phenyl-(benzyloxy-L-alaninyl)] phosphate (14)[2]

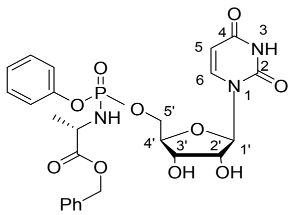

Procedure: standard procedure A and B

State: colourless wax (60 mg)

^{1}H-NMR (500 MHz, $CDCl_3$) δ 9.56 (s, 1H, -NH), 7.43 (t, J = 8.3 Hz, 2H, -C_6H_5, -H_6), 7.33 – 7.16 (m, 5H, -C_6H_5), 7.08 (m, 3H, -C_6H_5), 5.73 (m,1H, -$H_{1'}$), 5.57 (m, 1H, -H_5), 5.23 (s, 2H, -CH_2), 5.12 – 4.97 (m, 3H, -$H_{5'}$, -$H_{4'}$), 4.69 (s, 1H, -$H_{3'}$), 4.39 – 3.89 (m, 1H, -$H_{2'}$), 1.34 – 1.25 (m, 3H, -CH_3) ppm.

^{13}C-NMR (126 MHz, CDCl$_3$) δ 173.51 (C, C-aromatic), 163.71 (C, C-aromatic), 151.14 (C, C-aromatic), 150.42 (C, C-aromatic), 135.17 (C, C-aromatic), 129.86 (CH, C-aromatic), 129.83 (CH, C-aromatic), 128.67, 128.64 (CH, C-aromatic), 128.52, 128.50 (CH, C-aromatic), 128.40 (CH, C-aromatic), 128.21, 128.19 (CH, C-aromatic), 126.11 (CH, C-aromatic), 125.25, 125.21 (CH, C-aromatic), 120.11, 120.08 (CH, C-aromatic), 120.00, 119.96 (CH, C-aromatic), 115.66 (CH, C-aromatic), 102.70, 102.62 (CH, C-aromatic), 89.86 (CH, C-aliphatic, -$C_{1'}$), 82.65 (CH, C-aliphatic, -$C_{4'}$), 69.70 (CH, C-aliphatic, -$C_{3'}$), 67.33 (CH, C-aliphatic, -$C_{5'}$), 51.79 (CH_2, C-aliphatic), 31.59 (CH, C-aliphatic, -$C_{2'}$), 14.20 (CH_3, C-aliphatic) ppm.

^{31}P-NMR (202 MHz, CDCl$_3$) δ 3.06, 2.94 ppm.

MS(ES)$^+$ m/z 584.18 [M+ Na]$^+$, 562.23 [M+ H]$^+$

3.6 Thymidine

Table 6. Thymidine phosphoramidate and by side products. *Reagent and conditions*: a) *t*-BuMgCl (2-3 equivalents), solvent; b) NMI (6.3 equivalents), solvent. In blue and bold, best conditions presented in the main paper.

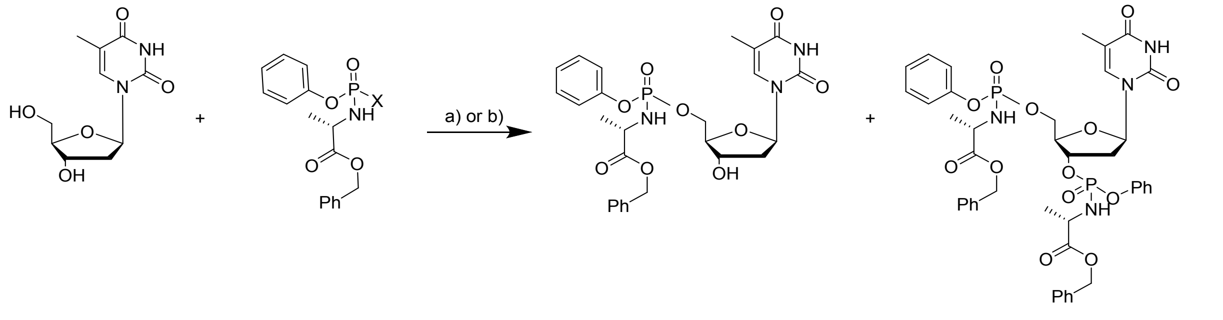

(6) **(8), (9)** **(15)** **(24)**

(8): X = -pNO$_2$Ph *reagents and condition* a)
(9): X = -Cl *reagents and condition* b)

5'-O-phosphoramidate

3', 5'-O, O-phosphoramidate (bis)

				Conventional heating (55 °C)				Microwave irradiation				
	**entry**	**Reagents**	**Solvent**	**Time (min)**	**Yield* (%)**			**Hold time (min)**	**T (°C)**	**Yield* (%)**		
					**(6)**	**(15)**	**(24)**			**(6)**	**(15)**	**(24)**
**Grignard method, scheme 1, (a)**	1	*t*-BuMgCl (3 Eq) + (**8**)	THF/NMP	20	22	13	65	2	65	100	-	-
	2	*t*-BuMgCl (2 Eq) + (**8**)	THF/NMP	60	9	40	51	20	65	6	25	69
	**3**	***t*-BuMgCl (0.5*4 Eq) + (8)**	**THF/NMP**	**120**	**16**	**45**	**39**	**60**	**65**	**55**	**43**	**2**
	4	*t*-BuMgCl (2 Eq) + (**8**)	1,4-dioxane	160	23	39	38	30	65	80	8	12
	5	*t*-BuMgCl (2 Eq) + (**8**)	THF/NMP					40	65	26	44	31
	6	*t*-BuMgCl (2 Eq) + (**8**)	DMF	40	5	29	66	40	65	30	45	25
**NMI method, scheme 1, (b)**	7	NMI + (**9**)	THF	20	51	29	20	3	65	45	55	-
	**8**	**NMI + (9)**	**THF**	**240**	**13**	**73**	**14**	**5**	**65**	**1**	**70**	**29**

* Conversion of the parent nucleoside into the desired 5'-protide was calculated on HPLC analysis (method 1).

3.6.1 Conditions for the optimisation study

3.6.2 HPLC spectra

Thymidine (**6**) – HPLC pu

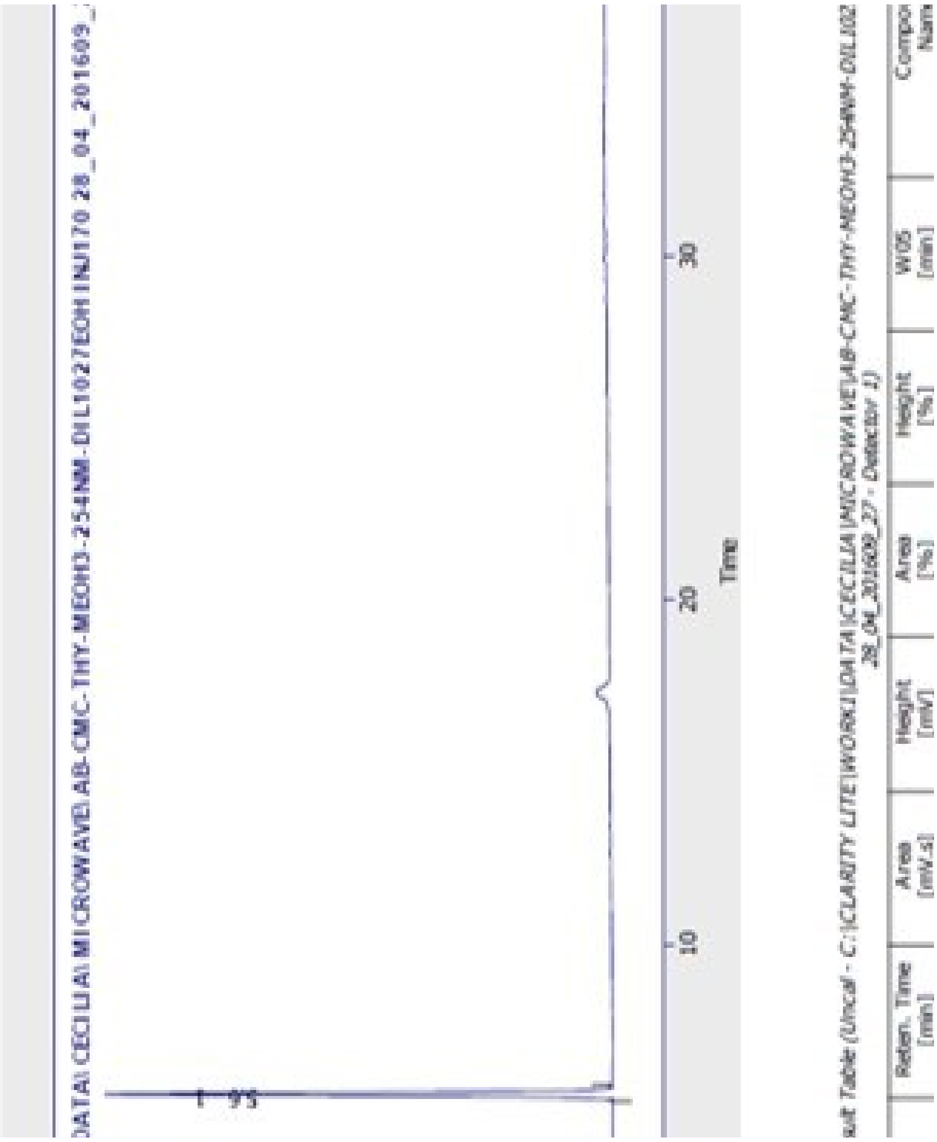

Compound (**15**) – HPLC p

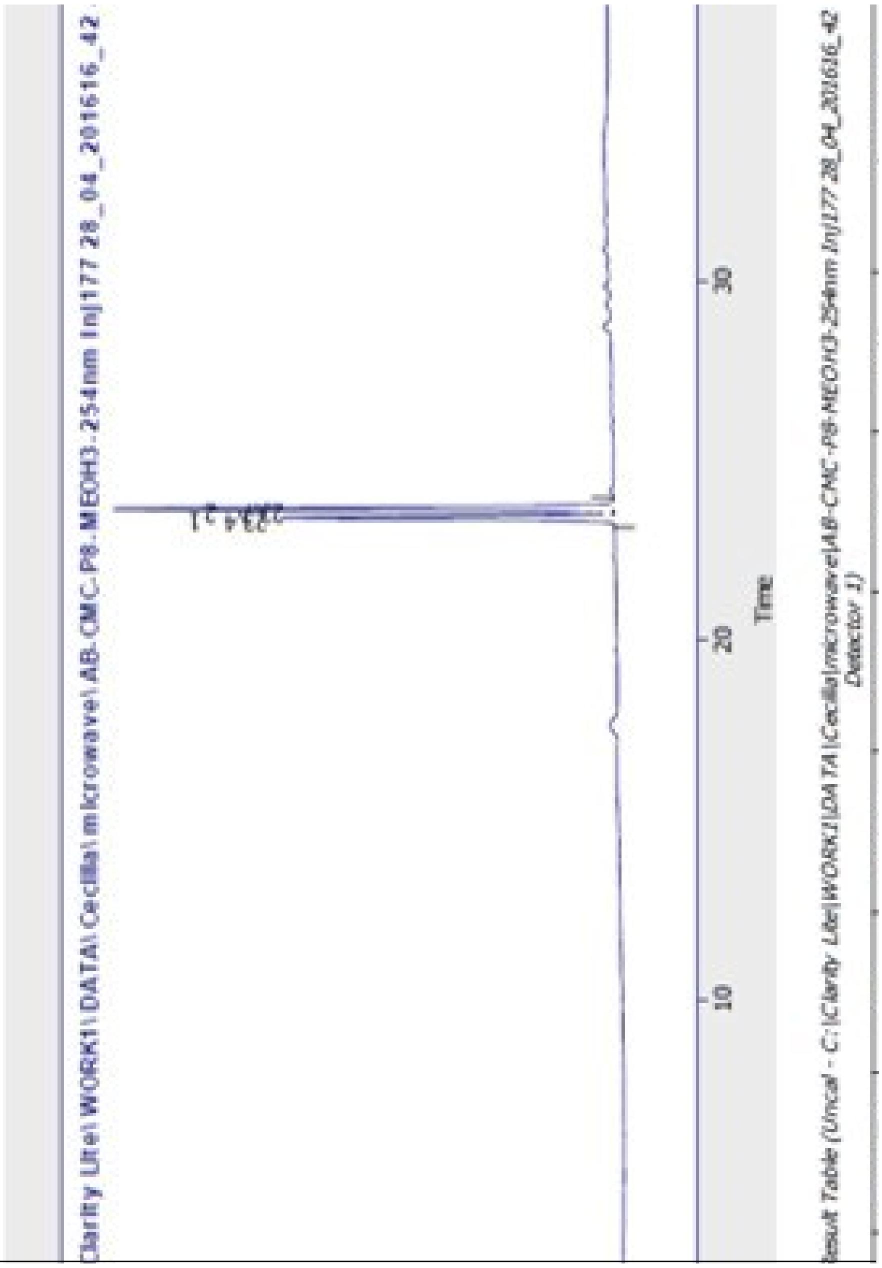

Compound (**24**) – HPLC purity

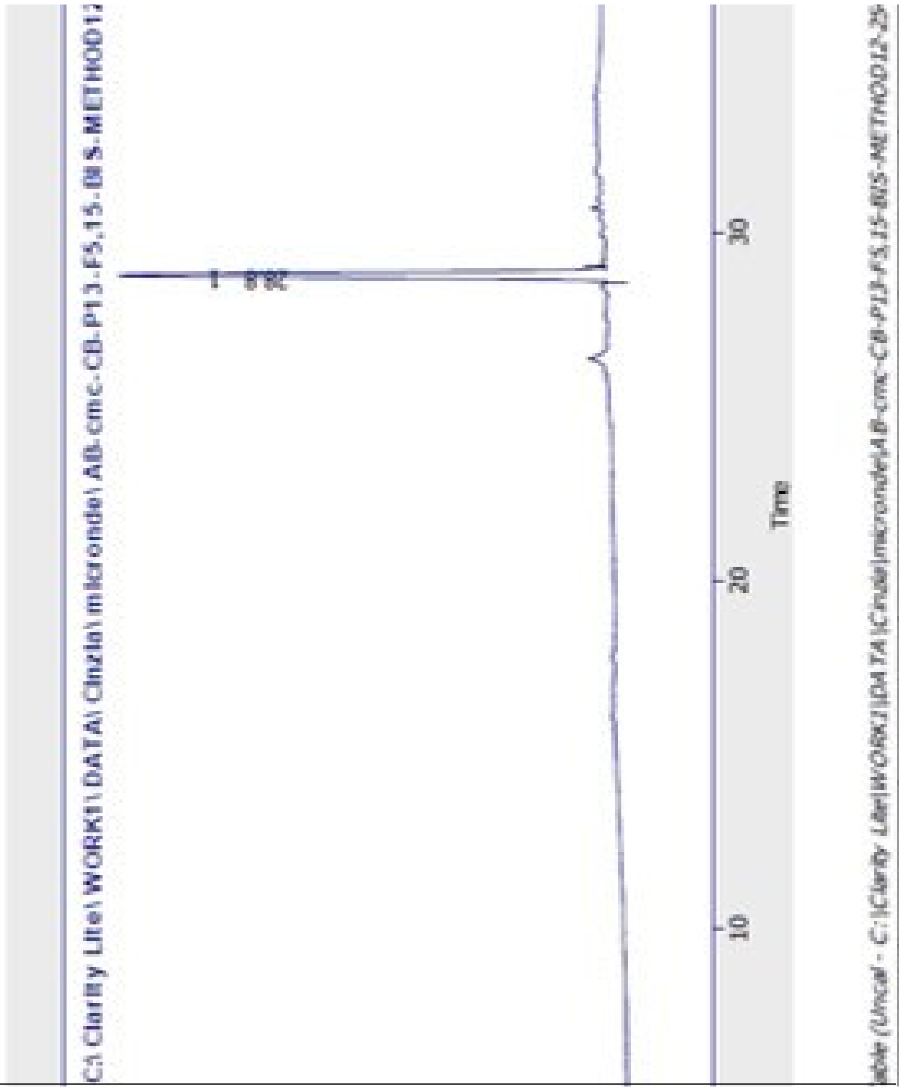

Reaction in HPLC Table 7 – entry 3 (conventional heating reaction):

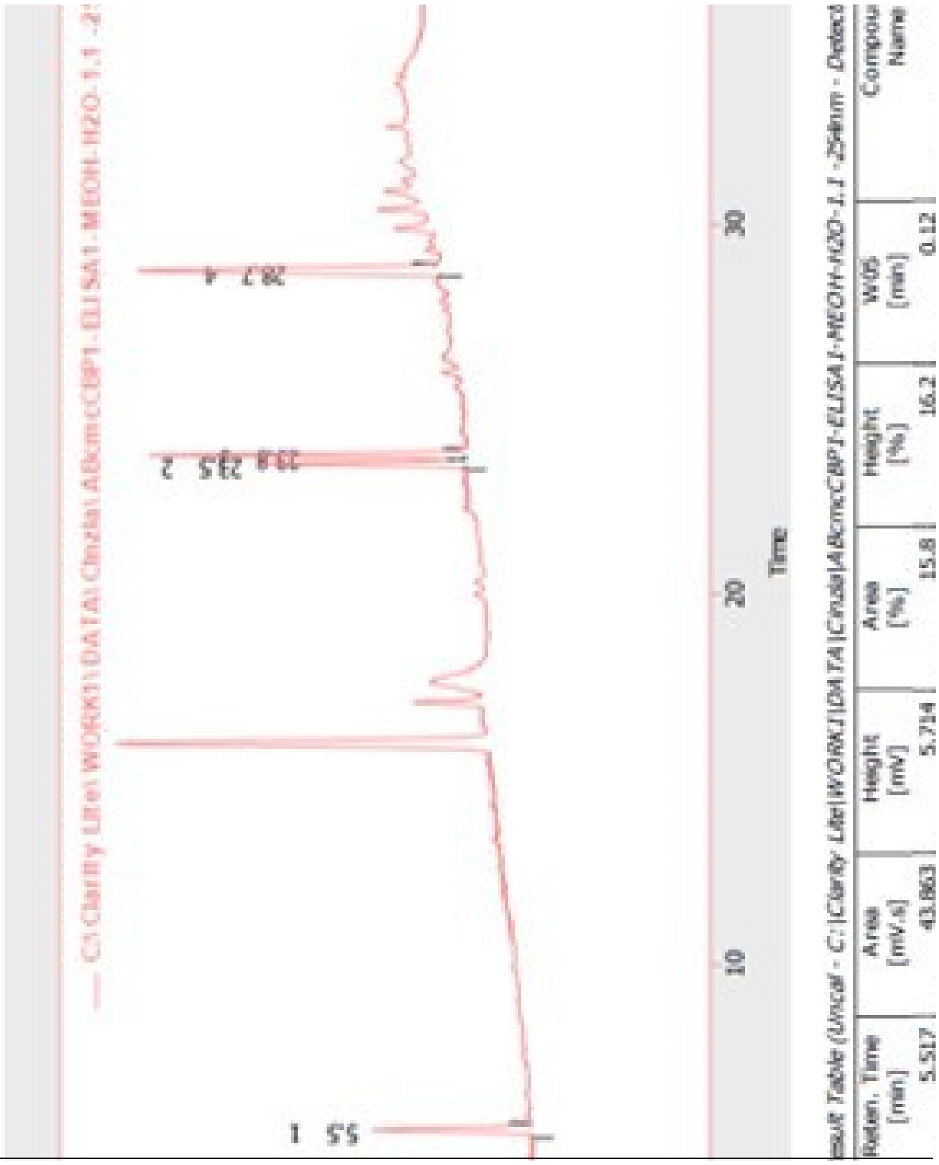

Reaction in HPLC Table 7 – entry 3 (microwave heating reaction)

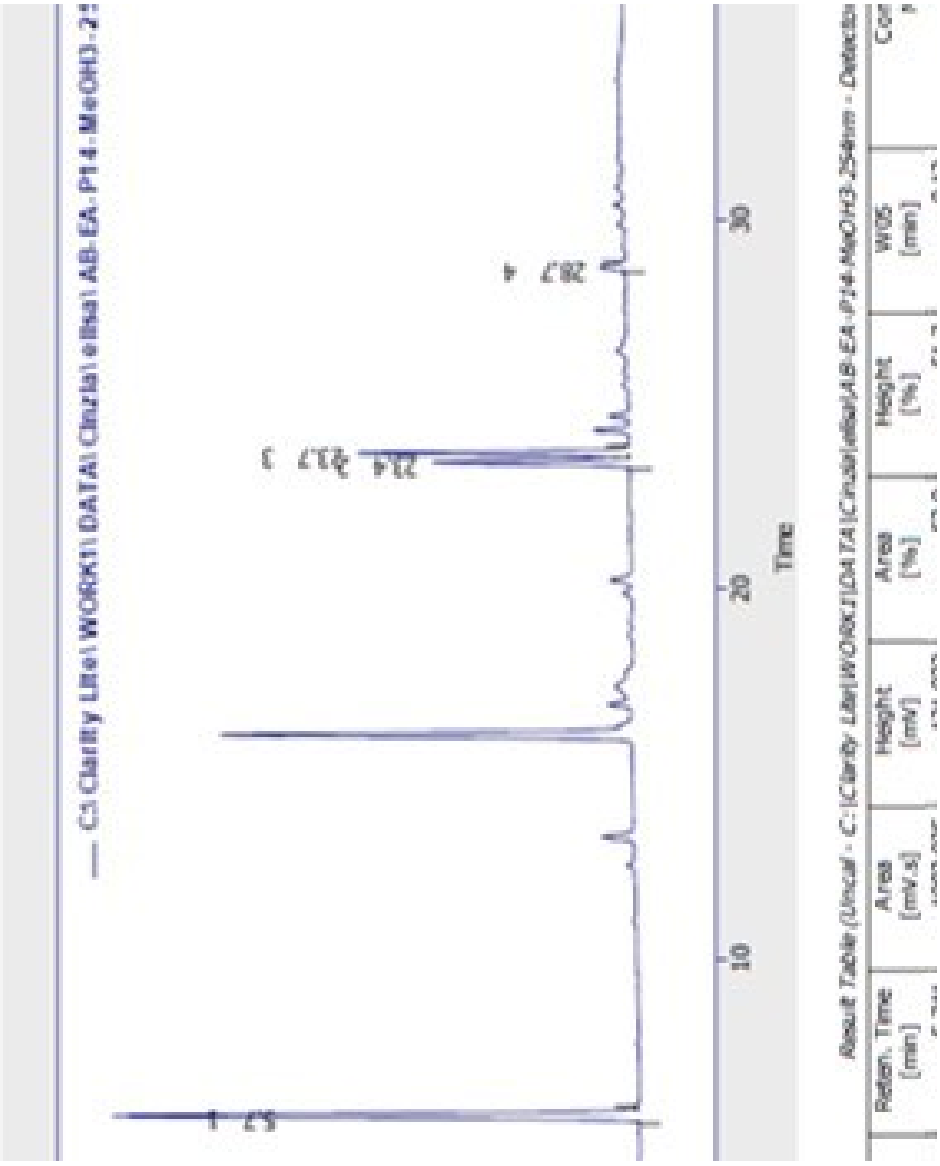

Reaction in HPLC Table 2 – entry 12 (conventional heating reaction)

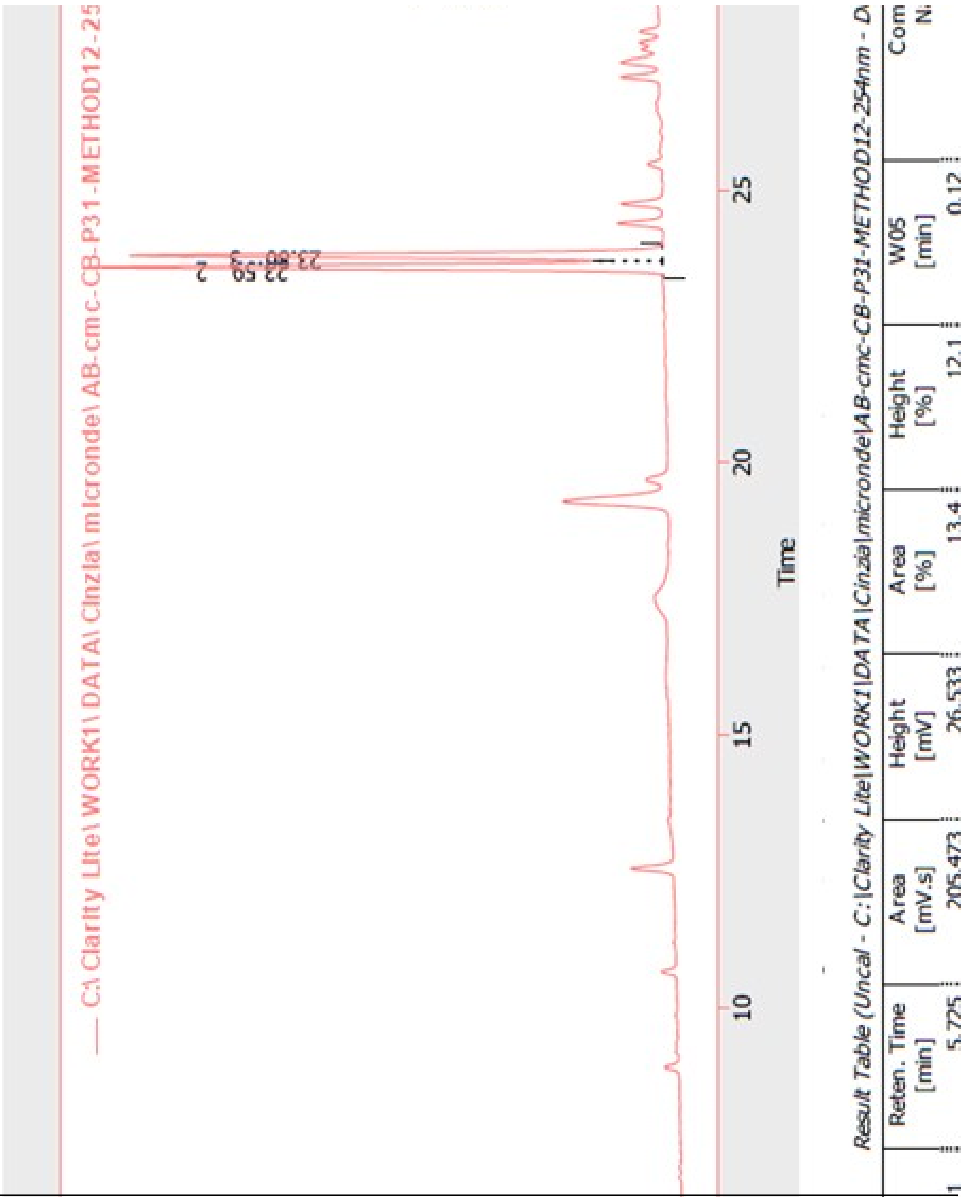

Reaction in HPLC Table 2 – entry 12 (microwave heating)

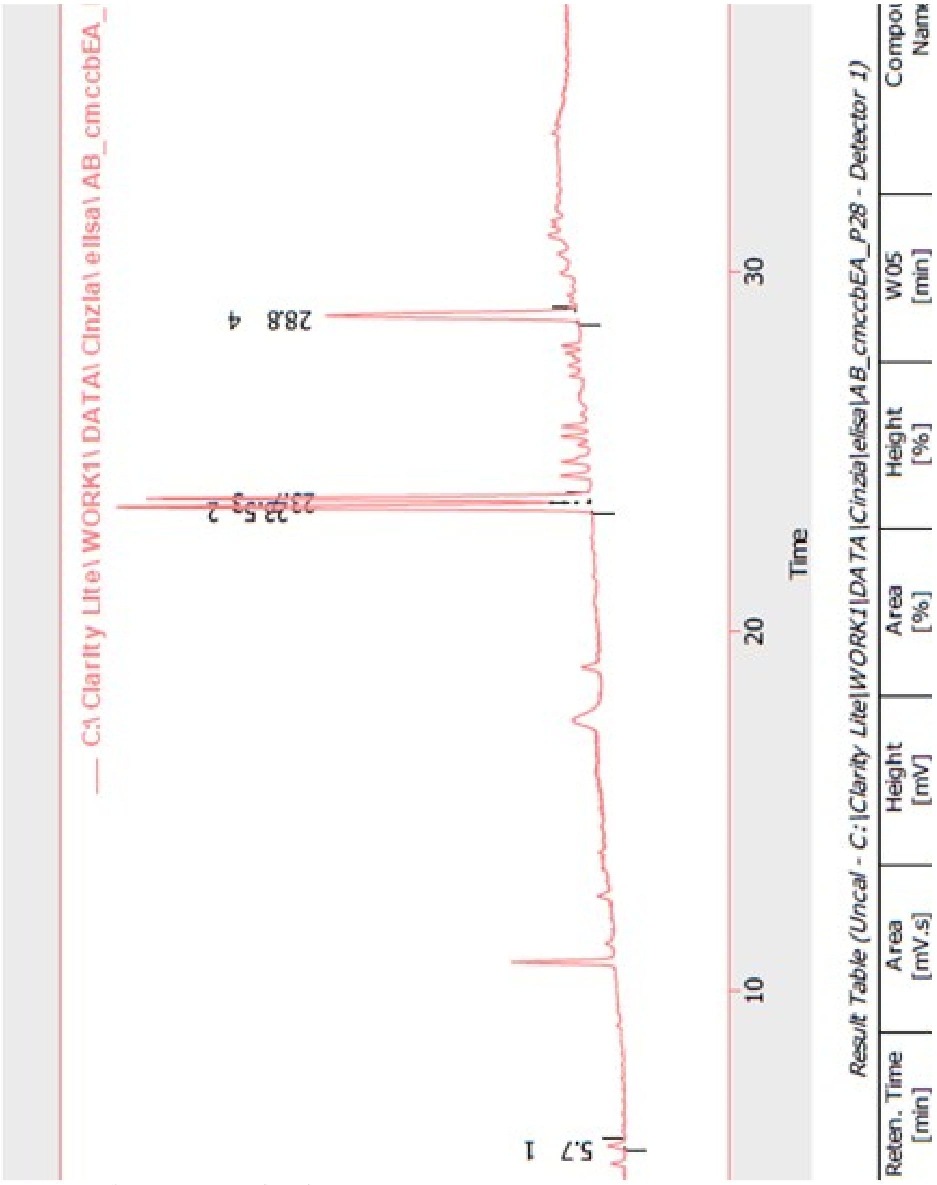

3.6.3 Spectroscopic and spectrometric characterisation

Thymidine-5'-O[phenyl-(benzyloxy-L-alaninyl)] phosphate (15)

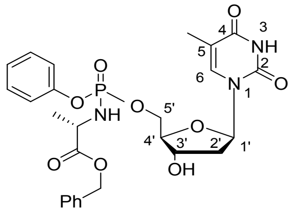

Procedure: standard procedure A and B

State: colourless wax (20 mg)

^{1}H-NMR (500 MHz, $CDCl_3$) δ 8.48 (s, 1H, -NH), 7.44-7.19 (m, 8H, $-C_6H_5$, $-H_6$), 7.10 (dd, J = 14.6, 7.1 Hz, 3H, $-C_6H_5$), 6.17 (m, 1H, $-H_{1'}$), 5.06 (s, 2H, $-CH_2$), 4.48 – 4.23 (m, 2H, $-H_{3'}$, -OH), 4.23 – 4.07 (m, 2H, $-H_{5'}$), 4.07 – 3.84 (m, 2H, -CH aliphatic, -NH), 3.83 – 3.58 (m, 1H,$-H_{4'}$), 2.34 – 2.18 (m, 1H, $-H_{2'}$), 2.12 – 1.93 (m, 1H, $-H_{2'}$), 1.80 (s, 3H, $-CH_3$, C_5), 1.41 – 1.25 (m, 3H, $-CH_3$ aliphatic) ppm.

^{13}C-NMR (126 MHz, $CDCl_3$) δ 174.03 (C, C-aromatic, C=O), 163.48 (C, C-aromatic, C=O), 150.52 (C,

C-aromatic), 135.62 (C, C-aromatic), 135.50 (C, C-aromatic), 135.38 (CH, C-aromatic), 129.86 (CH, C-aromatic), 128.70 (CH, C-aromatic), 128.63 (CH, C-aromatic), 128.27 (CH, C-aromatic), 125.33 (CH, C-aromatic), 120.07 (CH, C-aromatic), 120.00 (CH, C-aromatic), 111.23 (CH, C-aromatic), 84.84 (CH, C-aliphatic, -$C_{1'}$), 84.47 (CH, C-aliphatic, -$C_{4'}$), 70.50 (CH, C-aliphatic, -$C_{3'}$), 67.49 (CH_2, C-aliphatic), 65.69 (CH_2, C-aliphatic, -$C_{5'}$), 50.38 (CH, C-aliphatic), 39.81 (CH_2, C-aliphatic, -$C_{2'}$), 20.84 (CH_3, C-aliphatic), 12.40 (CH_3, C-aliphatic) ppm.

^{31}P-NMR (202 MHz, $CDCl_3$) δ 3.27, 2.84 ppm.

MS(ES)$^+$ m/z 582.16 [M+ Na]$^+$, 560.18 [M+ H]$^+$

Thymidine-3', 5'-O, O[phenyl-(benzyloxy-L-alaninyl)] phosphate (24)

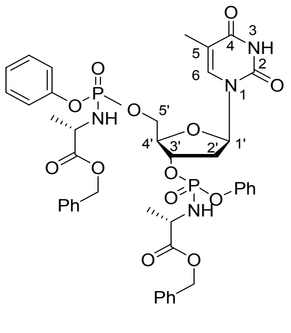

Procedure: standard procedure A and B

State: pale yellow wax (80 mg)

^{1}H-NMR (500 MHz, $CDCl_3$) δ 8.68 (s, 1H, -NH), 7.46 – 7.02 (m, 20H. –C_6H_5), 6.33 – 6.02 (m, 1H, -$H_{1'}$), 5.10 – 4.94 (m, 4H, -CH_2 aliphatic), 4.37 – 4.15 (m, 2H, -$H_{5'}$), 4.15 – 4.01 (m, 1H, -$H_{3'}$), 3.96 (m, 2H, -CH aliphatic), 3.88 – 3.52 (m, 1H, -NH), 2.47 – 2.17 (m, 2H, -$H_{2'}$), 1.98 – 1.83 (m, 1H, -$H_{4'}$), 1.83 – 1.71 (m, 3H, -CH_3), 1.35 – 1.27 (m, 6H, -CH_3) ppm.

^{13}C-NMR (126 MHz, $CDCl_3$) δ 173.22 (C, C-aromatic), 163.56 (C, C-aromatic), 150.42 (C, C-aromatic), 150.19 (C, C-aromatic), 150.13 (C, C-aromatic), 135.24 (C, C-aromatic), 135.21 (CH, C-aromatic), 135.18 (CH, C-aromatic), 135.11 (CH, C-aromatic), 129.82 (CH, C-aromatic), 129.66 (CH, C-aromatic), 128.69 (CH, C-aromatic), 128.65 (CH, C-aromatic), 128.59 (CH, C-aromatic), 128.52 (CH, C-aromatic), 128.48 (CH, C-aromatic), 128.21 (CH, C-aromatic), 126.99 (CH, C-aromatic), 125.34 (CH, C-aromatic), 125.25 (CH, C-aromatic), 124.94 (CH, C-aromatic), 120.65 (CH, C-aromatic), 120.57 (CH, C-aromatic), 120.24 (CH, C-aromatic), 120.14 (CH, C-aromatic), 119.44 (CH, C-aromatic), 111.48 (CH, C-aromatic), 84.34 (CH, C-aliphatic, -$C_{1'}$), 83.41 (CH, C-aliphatic, -$C_{3'}$), 67.31 (CH_2, C-aliphatic), 65.55 (CH, C-aliphatic, -$C_{5'}$), 50.34 (CH, C-aliphatic), 38.34 (CH, C-aliphatic, -$C_{4'}$), 30.01(CH, C-aliphatic, -$C_{2'}$), 20.77 (CH_3, C-aliphatic), 12.42 (CH_3, C-aliphatic) ppm.

^{31}P-NMR (202 MHz, $CDCl_3$) δ 3.97, 3.84, 3.71, 3.53 ppm.

MS(ES)$^+$ m/z 899.25 [M+ Na]$^+$, 877.25 [M+ H]$^+$

3.7 3’-Deoxythymidine

3.7.1 Conditions for the optimisation study

Table 8. 2’-Deoxythymidine phosphoramidate and by side products. *Reagent and conditions*: a) *t*-BuMgCl (2-3 equivalents), solvent; b) NMI (6.3 equivalents), solvent. In blue and bold, best conditions presented in the main paper.

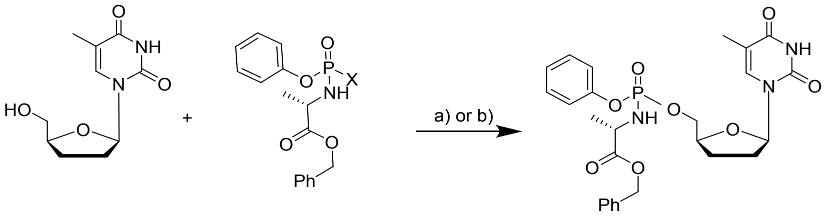

(7) **(8), (9)** **(16)**

(8): X = -pNO_2Ph *reagents and condition* a)
(9): X = -Cl *reagents and condition* b)

5'-O-phosphoramidate

				Conventional heating (55 °C)			Microwave irradiation			
	entry	Reagents	Solvent	Time (min)	Yield* (%)		Hold time (min)	T (°C)	Yield* (%)	
					(7)	(16)			(7)	(16)
**Grignard method,(a)**	**1**	***t*-BuMgCl (3 Eq) + (8)**	**DMF**	**150**	**2**	**98**	**1**	**65**	**19**	**81**
	2						5	75	17	83
	3						10	65	15	85
**NMI method, (b)**	**4**	**NMI +(9)**	**THF**	**155**	**16**	**84**	**3**	**65**	**11**	**89**

* Conversion of the parent nucleoside into the desired 5’-protide was calculated on UPLC analysis (method 1).

3.7.2 UPLC spectra

Reaction in Table 2 – entry 13 (conventional heating)

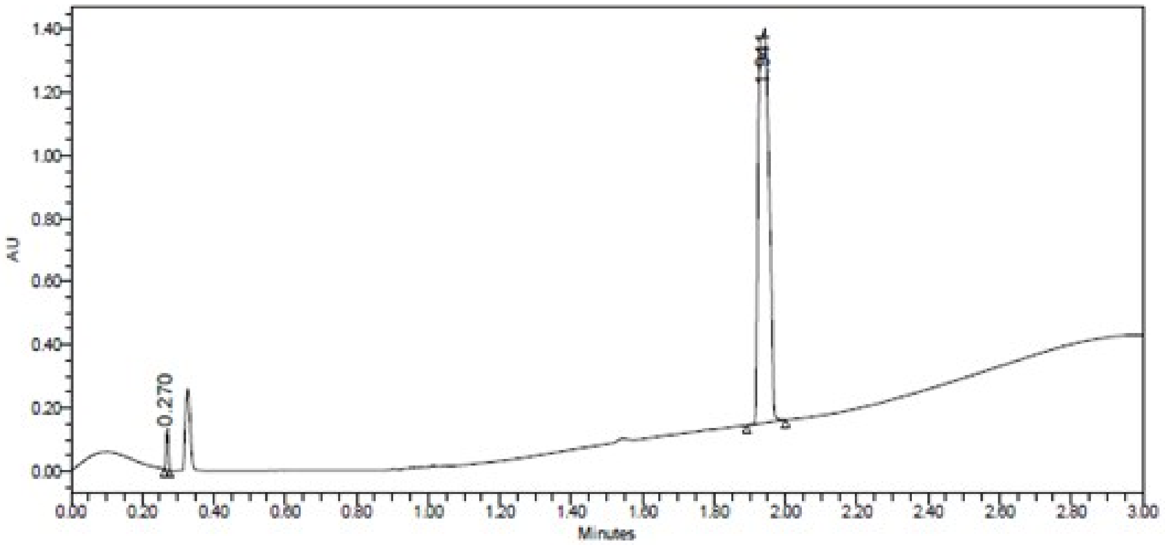

Processed Channel: PDA Spectrum
PDA MaxPlot (190.0 nm to 800.0 nm)
(PDA Spectrum (190-800)nm)

	Retention Time (min)	Area	% Area	Height	Purity1 Flag
1	0.270	58905	2.21	129047	No
2	1.941	2601816	97.79	1246826	No

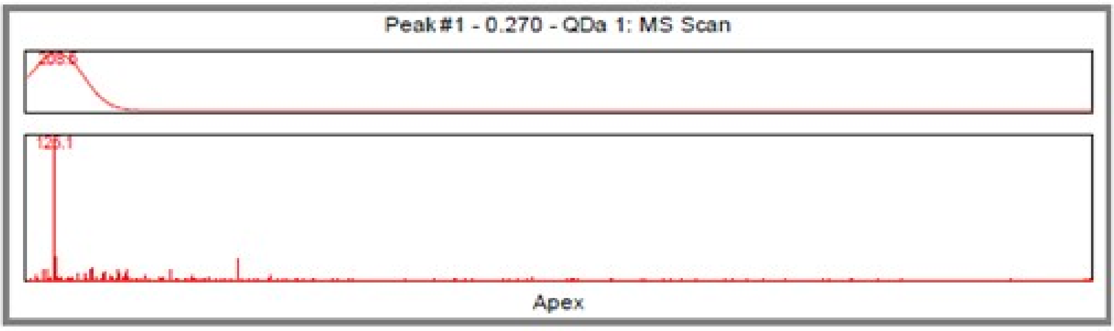

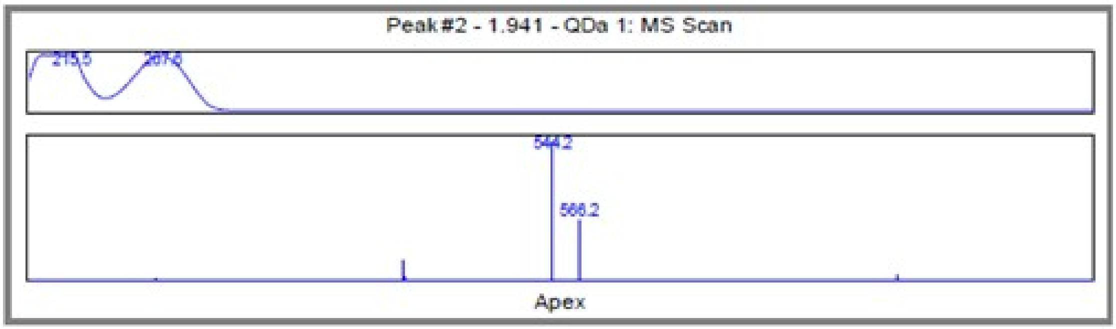

Reaction in Table 2 – entry 13 (microwave heating)

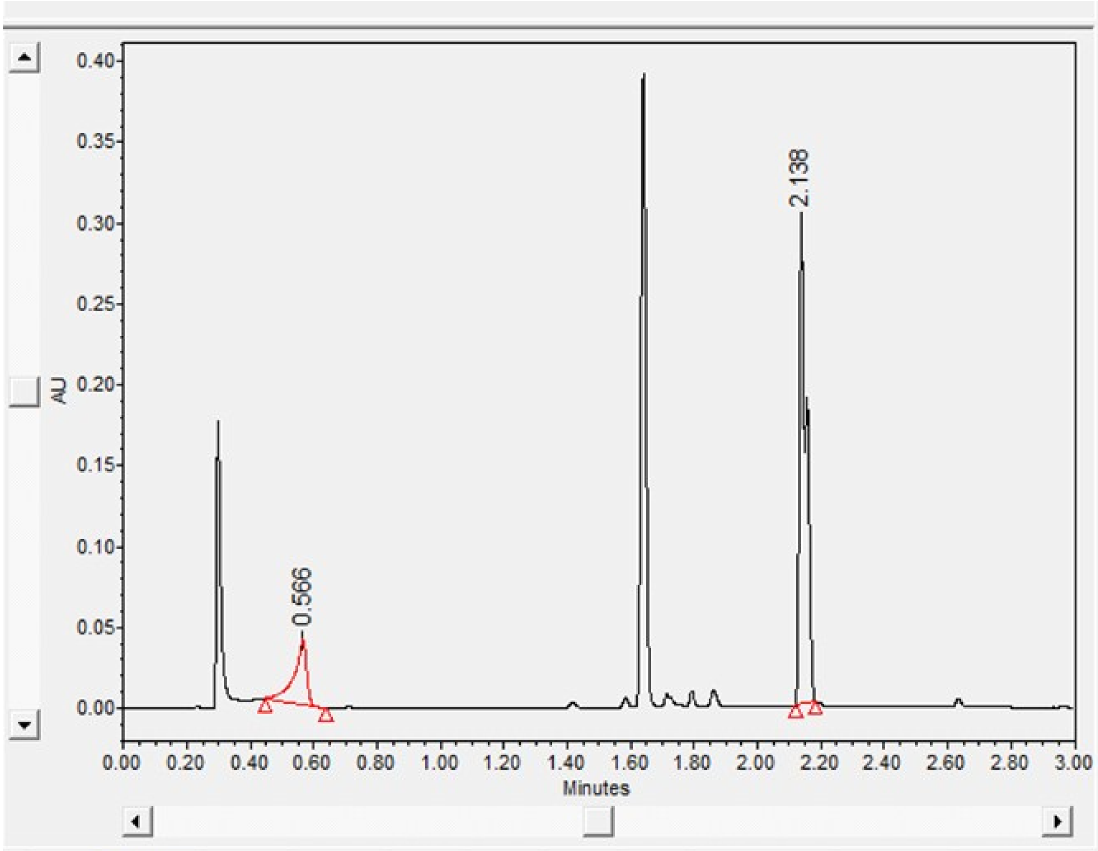

	Name	Retention Time (min)	Area (µV*sec)	% Area	Height (µV)	Int Type	Amount	Units	Peak Type	Peak Codes	ARTsec	A
1		0.566	109566	19.18	39860	bb			Unknown		33.961606	
2		2.138	461635	80.82	298983	bb			Unknown		128.259425	

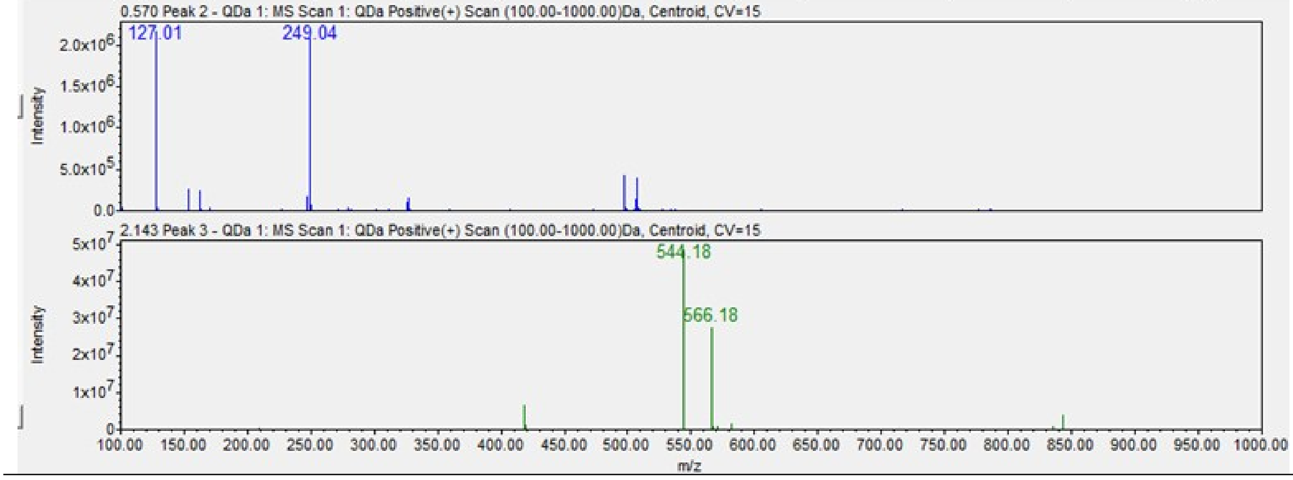

Reaction in Table 2 – entry 14 (conventional heating)

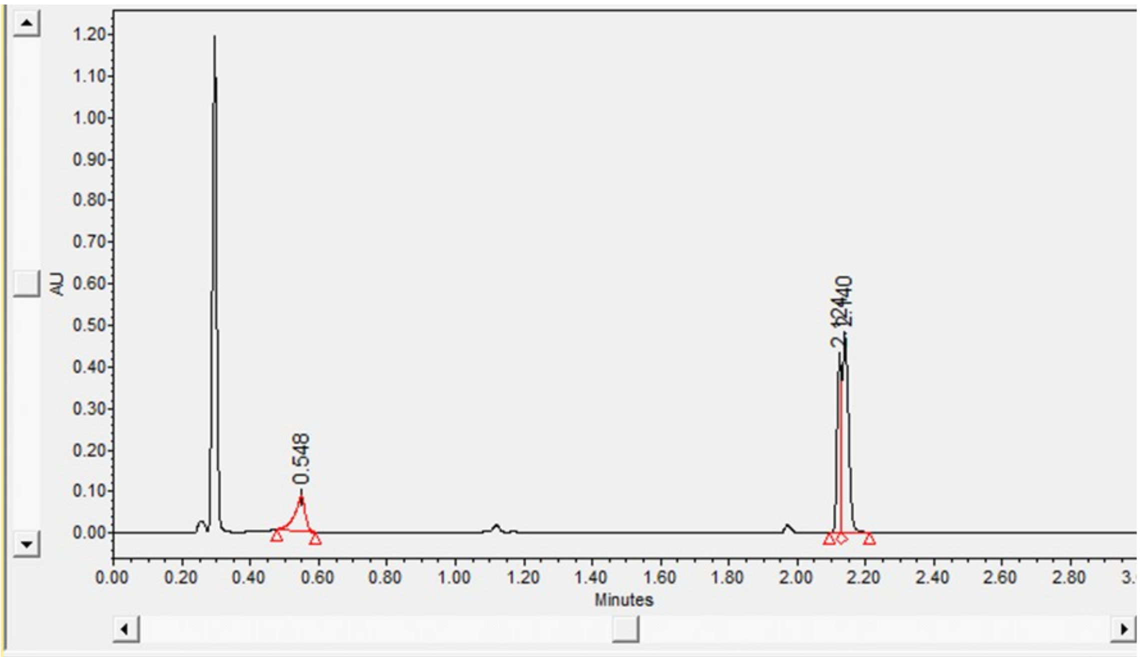

	Name	Retention Time (min)	Purity1 Angle	Purity1 Threshold	Purity1 Flag	Area (μV*sec)	% Area	Height (μV)	Int Type	Amount	Units	Peak Type	Peak Codes
1		0.548	48.054	90.000	☐	180162	15.93	83358	bb			Unknown	
2		2.124	10.106	90.000	☐	364344	32.22	417259	bV			Unknown	
3		2.140	18.141	90.000	☐	586279	51.85	466144	Vb			Unknown	

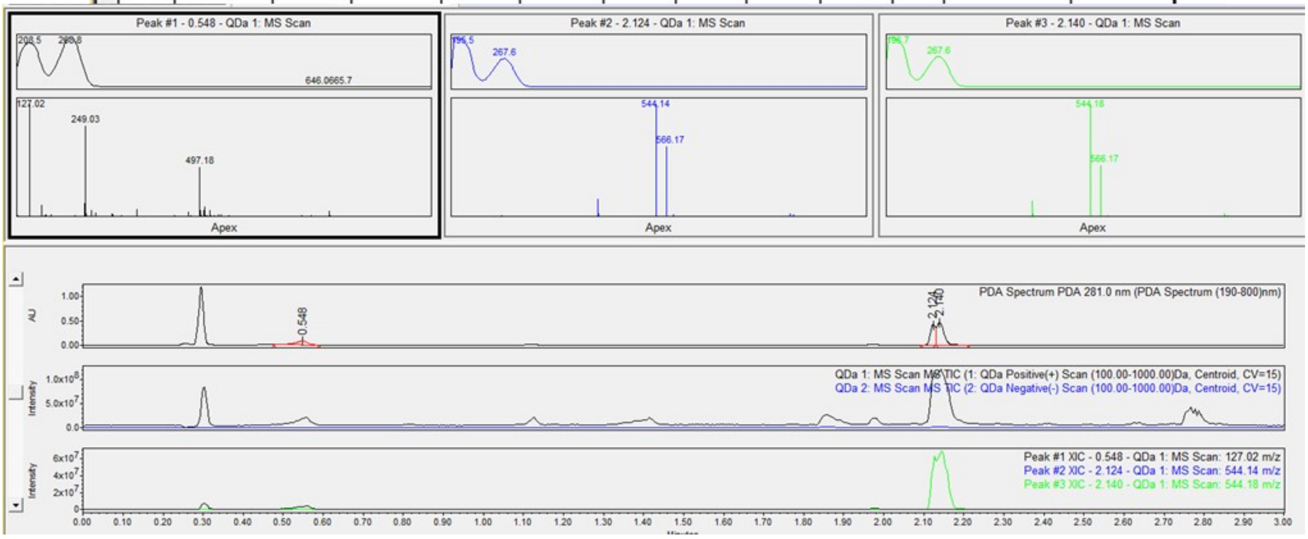

Reaction in Table 2 – entry 14 (microwave heating)

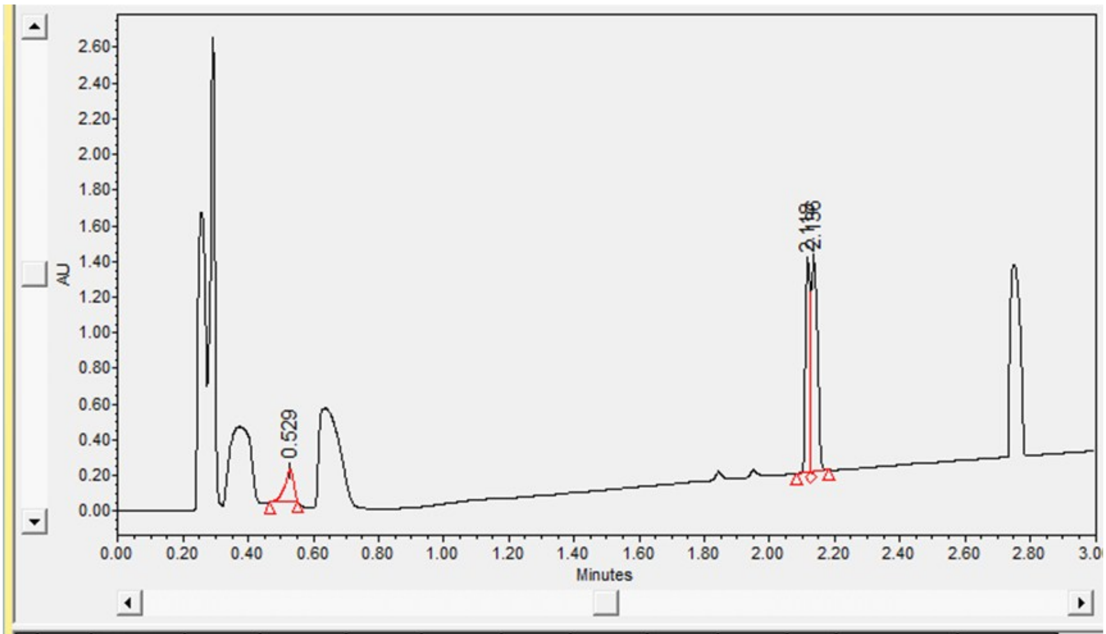

	Name	Retention Time (min)	Purity1 Angle	Purity1 Threshold	Purity1 Flag	Area (µV*sec)	% Area	Height (µV)	Int Type	Amount	Units	Peak Type	Peak Codes
1		0.529	38.346	90.000	☐	348305	11.18	180867	bb			Unknown	
2		2.118	16.394	90.000	☐	1199418	38.51	1168694	bV			Unknown	
3		2.136	21.038	90.000	☐	1566460	50.30	1178837	Vb			Unknown	

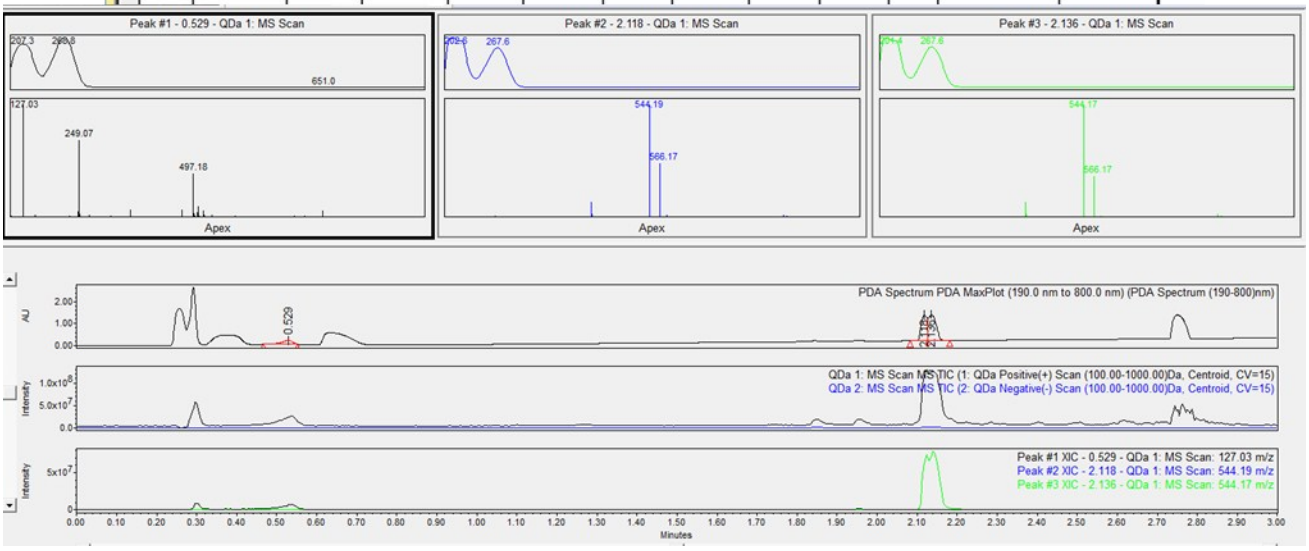

3.7.3 Spectroscopic and spectrometric characterisation

3'-Deoxy-thymidine-5'-O[phenyl-(benzyloxy-L-alaninyl)] phosphate (16)

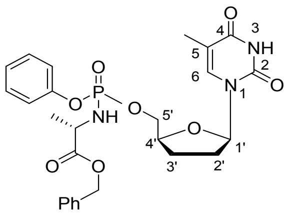

Procedure: standard procedure A and B

State: colourless wax (22 mg)

^{1}H-NMR (500 MHz, $CDCl_3$) δ 8.62 (d, J = 22.4 Hz, 1H, -H_6), 7.53 – 7.10 (m, 11H, -C_6H_5, -NH), 6.15 – 5.98 (m, 1H, -$H_{1'}$), 5.25 – 4.98 (m, 2H, -CH_2), 4.46 – 4.31 (m, 1H, -$H_{4'}$), 4.31 – 4.21 (m, 2H, -$H_{5'}$), 4.20 – 4.03 (m, 1H, CH aliphatic), 3.76 (m, 1H, -NH aliphatic), 2.44 – 2.19 (m, 1H, -$H_{3'}$), 2.13 – 1.96 (m, 2H, -$H_{2'}$, -$H_{3'}$), 1.90 (m, 4H, -CH_3, -$H_{2'}$), 1.40 (m, 3H, -CH_3) ppm.

^{13}C-NMR (126 MHz, $CDCl_3$) δ 171.25 (C, C-aromatic, C=O), 163.63 (C, C-aromatic, C=O), 150.51 (C, C-aromatic), 150.28 (C, C-aromatic), 135.46 (CH, C-aromatic), 135.14 (CH, C-aromatic, -C_6), 129.78 (CH, C-aromatic), 128.63 (CH, C-aromatic), 128.28 (CH, C-aromatic), 125.19 (CH, C-aromatic), 121.11 (CH, C-aromatic), 110.79 (CH, C-aromatic). 85.75 (CH, C-aliphatic, -$C_{1'}$), 78.44 (CH, C-aliphatic, -$C_{4'}$), 67.36 (CH_2, C-aliphatic, d, J = 2.52 Hz, -$C_{5'}$), 67.30 (CH_2, C-aliphatic, *J* = 2.52 Hz, -CH_2 aliphatic), 50.37 (CH, C-aliphatic), 31.82 (CH_2, C-aliphatic, -$C_{3'}$), 25.58 (CH_2, C-aliphatic, -$C_{2'}$), 20.99 (CH_3, C-aliphatic), 12.48 (CH_3, C-aliphatic) ppm.

^{31}P-NMR (202 MHz, $CDCl_3$) δ 2.90, 2.62 ppm.

MS(ES)$^+$ m/z 566.20 $[M+ Na]^+$, 544.19 $[M+ H]^+$

4. Spectroscopic characterisation of compounds (8), (9), (17) – (23)

Benzyl ((4-nitrophenoxy)(phenoxy)phosphoryl)-L-alaninate (8)[1]

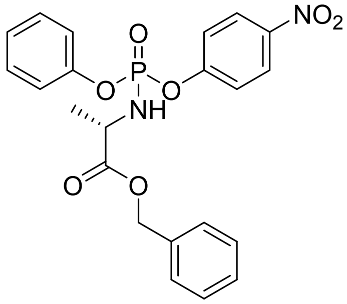

Procedure: phenol (0.735 g, 7.81 mmol) was dissolved in dry DCM (16 mL) and triethyl amine (0.12 mL, 8.5 mmol) was added drop wise. At this mixture, a solution of 4-nitrophenyl phosphorodichloridate (2g, 7.81 mmol) in dry DCM (16 mL) cooled to -78 °C was added drop wise. The reaction mixture was stirred at -78 °C for 1 hour. L-alanine benzyl ester *p*-tosylate salt (0.275 g, 7.8 mmol) was added. The reaction mixture was allowed to attain room temperature and stirred for additional 2 hours. The solvent was removed under reduced pressure, the crude residue was purified by column chromatography on silica gel using DCM/MeOH (100% → 80%:20% as eluent) to afford compound (**8**) as a colourless oil (yield: 90%).

^{1}H-NMR (500 MHz, CDCl$_3$) δ 8.08 (m, 2H, -C_6H_5), 7.31-7.09 (m, 12H, -C_6H_5), 5.10-5.00 (m, 2H, -CH_2), 4.17-3.95 (m, H, -CH), 1.34 (d, 3H, *J*=4.0 Hz, -CH_3) ppm.

^{31}P-NMR (202 MHz, CDCl$_3$) δ -3.22, -3.35 ppm.

Benzyl (chloro(phenoxy)phosphoryl)-L-alaninate (9)[2]

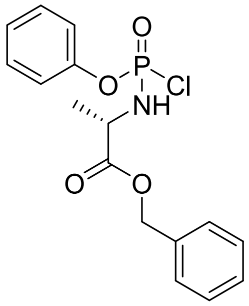

Procedure: L-alanine benyl ester *p*-tosylate salt (0.50 g, 1.42 mmol) was suspended in dry DM (15 mL) under an argon atmosphere and phenyl dichlorophosphate (0.21 mL, 1.42 mmol) was added at room temperature. The mixture was cooled to -78 °C before adding triethylamine (0.40 mL, 2.84 mmol) drop wise. After stirring 1 hour at -78 °C, the reaction mixture was allowed to attain room temperature and stirred for additional 2 hours. The solvent was removed under reduced pressure and the crude residue was purified by column chromatography on silica gel eluting with hexane/ethyl acetate (50:50 %v/v) to obtain compound (**9**) (yield: 94%) as a colourless oil.

^{1}H-NMR (500 MHz, $CDCl_3$) δ 7.31-7.10 (m, 10H, -C_6H_5), 5.12-5.10 (s, 2H, -CH_2), 4.57-4.46 (m, 1H, -NH), 4.22- 4.06 (m, 1H, -CH), 1.43-1.42 (d, 3H, *J*=7.0 Hz, -CH_3) ppm.

^{31}P-NMR (202 MHz, $CDCl_3$) δ 8.03, 7.79 ppm.

Isopropyl ((4-nitrophenoxy)(phenoxy)phosphoryl)-L-alaninate (18)[3]

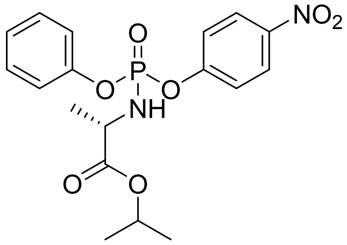

Procedure: phenol (0.735 g, 7.81 mmol) was dissolved in dry DCM (16 mL) and triethyl amine (0.12 mL, 8.5 mmol) was added drop wise. At this mixture, a solution of 4-nitrophenyl phosphorodichloridate (2g, 7.81 mmol) in dry DCM (16 mL) cooled to -78 °C was added drop wise. The reaction mixture was stirred at -78 °C for 1 hour. L-alanine isopropyl ester hydrochloride (0.275 g, 7.8 mmol) was added. The reaction mixture was allowed to attain room temperature and stirred for additional 2 hours. The solvent was removed under reduced pressure, the crude residue was purified by column chromatography on silica gel using hexane to hexane/ethyl acetate 75:25 v/v as elution system to obtain compound (**23**) as a colourless oil (yield: 86%).

^{1}H-NMR (500 MHz, $CDCl_3$) δ 8.25 (m, 2H, -C_6H_5), 7.44-7.35 (m, 4H, -C_6H_5), 7.28-7.21 (m, 3H, -C_6H_5), 5.08-4.98 (m, 1H, -CH isopropyl), 4.17-4.06 (s, 1H, -NH), 3.93 (m, 1H, -CH), 1.43-1.42 (d, *J*=2.4 Hz, 3H, -CH_3), 1.24-1.19 (m, 6H, -CH_3 isopropyl) ppm.

^{31}P-NMR (202 MHz, $CDCl_3$) δ -3.15, -3.19 ppm.

Isopropyl (chloro(phenoxy)phosphoryl)-L-alaninate (19)[3]

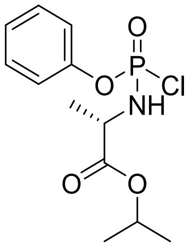

L-alanine isopropyl ester hydrochloride (1 g, 5.97 mmol) was dissolved in dry DCM (15 mL) under an argon atmosphere and phenyl dichlorophosphate (0.90 mL, 5.97 mmol) was added at room temperature. The mixture was cooled to -78 °C before adding triethylamine (1.66 mL, 11.93 mmol) drop wise. After stirring 1 hour at -78 °C, the reaction mixture was allowed to attain room temperature and stirred for additional 2 hours. The solvent was removed under reduced pressure and the crude residue was suspended in anhydrous diethyl ether under a nitrogen atmosphere and stirred for 15 minutes, then filtered under vacuum protected by a flow of nitrogen. The ethereal filtrate was evaporated under reduced pressure to obtain compound (**23**) as a colourless oil (yield: 95%).

^{1}H-NMR (500 MHz, $CDCl_3$) δ 7.34-7.16 (m, 5H, -C_6H_5), 5.06-4.97 (m, 1H, -CH isopropyl), 4.29 (s, 1H, -CH), 4.13-3.99 (m, 1H, -NH), 1.43-1.42 (d, *J*=2.4 Hz, 3H, -CH_3), 1.23-1.18 (m, 6H, -CH_3 isopropyl) ppm.

^{31}P-NMR (202 MHz, $CDCl_3$) δ 8.09, 7.72 ppm.

Benzyl (diphenoxyphosphoryl)-L-alaninate (20)

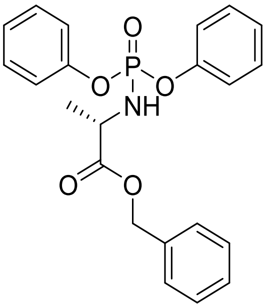

Procedure: L-alanine benzyl ester p-tosylate salt (0.50g, 1.42 mmol) was dissolved in anhydrous DCM under a nitrogen atmosphere and diphenylchlorophosphate (0.30 mL, 1.42 mmol) was added at room temperature. The mixture was cooled to -78 °C before adding triethylamine (0.40 mL, 2.85 mmol) drop wise. The reaction mixture was stirred at -78 °C for 1 hour then allowed to attain room temperature and stirred for additional 2 hours. The solvent was removed under reduced pressure. The crude residue was suspended in ethyl acetate and stirred for 15 minutes, then filtered under vacuum. The filtrate was evaporated under reduced pressure and the crude residue was purified by flash column chromatography on silica gel (hexane to hexane/acetone 70:30 v/v) to obtain compound (**25**) as a colourless oil (yield: 96%).

^{1}H-NMR (500 MHz, $CDCl_3$) δ 7.39-7.15 (m, 15H, $-C_6H_5$), 5.14 (s, 2H, $-CH_2$), 4.27-4.12 (m, 2H, -CH, -NH), 1.41 (d, J=7.0 Hz, 3H, $-CH_3$) ppm.

^{13}C-NMR (126 MHz, $CDCl_3$) δ 173.13 (d, $J_{C\text{-}C\text{-}N\text{-}P}$=7.5 Hz, C, C=O), 150.72 (C, C-aromatic), 135.29 (C, C-aromatic), 129.72 (d, $J_{C\text{-}C\text{-}O\text{-}P}$=4.4 Hz, CH, C-aromatic), 128.66 (C, C-aromatic), 128.50 (CH, C-aromatic), 128.23 (CH, C-aromatic), 125.07 (CH, C-aromatic), 120.30 (CH, C-aromatic), 67.23 (CH_2, C-aliphatic), 50.56 (d, $J_{C\text{-}N\text{-}P}$=1.0 Hz, CH, C- aliphatic), 20.95 (d, $J_{C\text{-}C\text{-}N\text{-}P}$=4.9 Hz, CH_3, C- aliphatic) ppm.

^{31}P-NMR (202 MHz, $CDCl_3$) δ – 2.79 ppm.

Isopropyl (diphenoxyphosphoryl)-L-alaninate (21)[3]

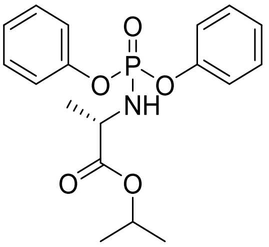

Procedure: L-alanine isopropyl ester hydrochloride (0.50g, 2.98 mmol) was dissolved in anhydrous DCM under a nitrogen atmosphere and diphenylchlorophosphate (0.62 mL, 2.98 mmol) was added at room temperature. The mixture was cooled to -78 °C before adding triethylamine (0.83 mL, 5.97 mmol) drop wise. The reaction mixture was stirred at -78 °C for 1 hour, then allowed to attain room temperature and stirred for additional 2 hours. The solvent was removed under reduced pressure. The crude residue was suspended in ethyl acetate ad stirred for 15 minutes, then filtered under vacuum. The filtrate was evaporated under reduced pressure and the crude residue was purified by flash column chromatography on silica gel (hexane to hexane/acetone 70:30 v/v) to obtain compound (**26**) as a colourless oil (yield: 83%).

^{1}H-NMR (500 MHz, $CDCl_3$) δ 7.38-7.32 (m, 4H, -C_6H_5), 7.29-7.24 (m, 4H, -C_6H_5), 7.21-7.17 (m, 2H, -C_6H_5), 5.07-4.98 (m, 1H, -CH isopropyl), 3.79 (m, 1H, -CH), 1.39 (d, *J*=7.0 Hz, 3H, -CH_3), 1.24 (d, *J*=4.2Hz, 6H, -CH_3 isopropyl) ppm.

^{13}C-NMR (126 MHz, $CDCl_3$) δ 173.72 (d, $J_{C\text{-}C\text{-}N\text{-}P}$=7.4 Hz, C, C=O), 150.69 (C, C-aromatic), 129.71 (d, $J_{C\text{-}C\text{-}O\text{-}P}$=2.0 Hz, CH, C-aromatic), 125.06 (CH, C-aromatic), 120.26 (CH, C-aromatic), 69.34 (CH, C-aliphatic), 50.56 (CH, C- aliphatic), 21.61 (CH_3, C- aliphatic), 21.10 (d, $J_{C\text{-}C\text{-}N\text{-}P}$=43.1 Hz, CH_3, C-aliphatic) ppm.

^{31}P-NMR (202 MHz, $CDCl_3$) δ -2.81ppm.

Adenosine-5'-O[phenyl-(benzyloxy-L-alaninyl)] phosphate (17)[3]

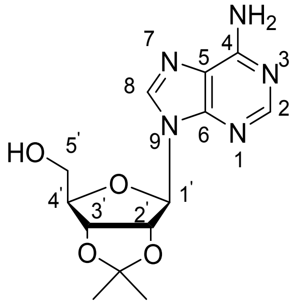

Procedure: a 70% aqueous solution of perchloric acid (0.56 mL, 6.55 mmol) was added drop wise to a stirred suspension of adenosine (1g, 3.74 mmol) in acetone at room temperature. The resulting solution was stirred for 30 minutes, then added of saturated aqueous $NaHCO_3$ solution and concentrated to dryness by co-evaporation with ethanol. The crude residue was purified by flash column chromatography on silica gel (DCM to DCM/MeOH 90:10 v/v) to give the title compound (**17**) as a white powder (yield: 95%).

^{1}H-NMR (500 MHz, $CDCl_3$) δ 8.35 (s, 1H, -H_2), 8.16 (s, 1H, -H_8), 7.35 (s, 2H, -NH_2), 6.13 (d, *J*=3.1 Hz, 1H, -$H_{1'}$), 5.35 (dd, *J*=6.1, 3.1 Hz, 1H, -$H_{2'}$), 5.24 (t, *J*=5.6Hz, 1H, -$H_{5'}$), 4.97 (dd, *J*=6.1, 2.5 Hz, 1H, -$H_{3'}$),4.22 (td, *J*=4.8, 2.5 Hz, 1H, -$H_{4'}$), 3.58-3.47 (m, 1H, -$H_{5'}$), 1.56 (s, 3H, -CH_3 acetonide), 1.34 (s, 3H, -CH_3 acetonide) ppm.

2',3'-O,O-isopropylidene-adenosine-5'-O[phenyl-(benzyloxy-L-alaninyl)] phosphate (22)

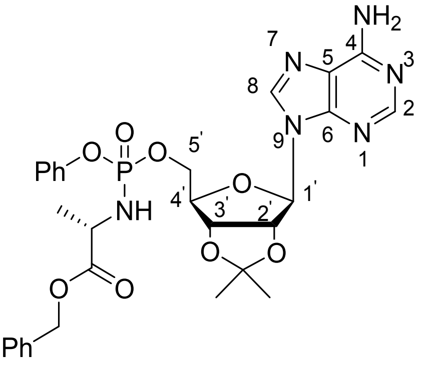

Procedure: standard procedures A and B

State: colourless wax

^{1}H-NMR (500 MHz, $CDCl_3$) δ 8.24-8.21 (m, 2H, -H_2, -H_8), 7.34-7.26 (m, 8H, -C_6H_5), 7.17-7.08 (m, 2H, -C_6H_5), 6.21-6.19 (d, *J*=2.5 Hz, 1H, -$H_{1'}$), 5.37-5.27 (m, *2*H, -CH_2), 4.49-4.41 (m, *1*H, -$H_{4'}$), 4.34-4.18 (m, *2*H, -$H_{5'}$), 3.99-3.91 (m, 1H, -NH), 3.63 (m, 1H, -CH), 3.41 (m, 1H, -$H_{3'}$), 3.34 (m, 1H, -$H_{2'}$), 1.60 (s, 3H, -CH_3 acetonide), 1.38 (s, 3H, -CH_3 acetonide), 1.31-1.26 (m, 3H, -CH_3 aliphatic) ppm.

^{13}C-NMR (126 MHz, $CDCl_3$) δ 173.42 (-C, C=O), 173.20 (-C, C=O), 155.96 (C, C-aromatic), 152.67 (CH, C-aromatic), 150.57 (C, C-aromatic), 148.88 (C, C-aromatic), 140.18 (CH, C-aromatic), 135.77 (C, C-aromatic), 129.35 (CH, C-aromatic), 128.18 (CH, C-aromatic), 127.96 (CH, C-aromatic), 127.90 (CH, C-aromatic), 127.86 (CH, C-aromatic), 124.75 (CH, C-aromatic), 119.98 (CH, C-aromatic), 119.97 (CH, C-aromatic), 119.94 (CH, C-aromatic), 119.93 (CH, C-aromatic), 119.18 (C, C-aromatic), 114.11 (C, C-aromatic), 90.36 (CH, C-aliphatic, -$C_{1'}$), 85.05 (CH, C-aliphatic, -$C_{4'}$), 84.01 (CH, C-aliphatic, -$C_{2'}$), 81.29 (CH, C-aliphatic, -$C_{3'}$), 66.56 (CH_2, C- aliphatic), 66.21 (CH, C-aliphatic, -$C_{5'}$), 50.13 (CH, C-aliphatic), 26.06 (CH_3, C- aliphatic), 24.20 (CH_3, C- aliphatic), 18.98 (CH_3, C- aliphatic) ppm.

^{31}P-NMR (202 MHz, $CDCl_3$) δ 3.72, 3.40 ppm.

MS(ES)$^+$ m/z 647.3 $[M+ Na]^+$, 625.3 $[M+ H]^+$

2',3'-O,O-isopropylidene-adenosine-5'-O[phenyl-(isopropoxy-L-alaninyl)] phosphate (23)[3]

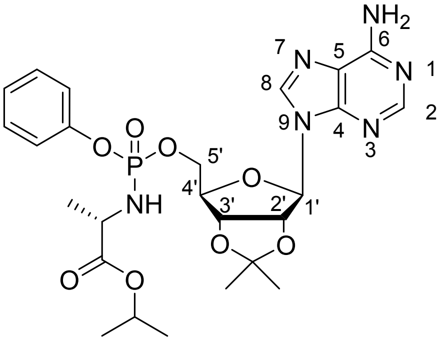

Procedure: standard procedures A and B

Procedure: standard procedure A and B

State: colourless wax

^{1}H-NMR (500 MHz, $CDCl_3$) δ 8.16 (m, 1H, -H_2), 8.12-8.09 (s, 2H, -H_8, -NH_2), 7.24-7.17 (m, 2H, -C_6H_5), 7.09-7.00 (m, 3H, -C_6H_5), 6.12-6.08 (d, J=2.5 Hz, 1H, -$H_{1'}$), 5.30-5.20 (d, J=6.3, 2.5 Hz, 1H, -$H_{2'}$), 5.05-5.00 (d, J=6.3, 3.1 Hz, 1H, -$H_{5'}$), 4.85-4.80 (m, 1H, -$H_{3'}$), 4.43-4.332 (m, 1H, -$H_{4'}$), 4.25-4.11 (m, 2H, -$H_{5'}$, -NH), 3.76-3.68 (m, 1H, -CH), 1.50 (s, 3H, -CH_3, acetonide), 1.29 (s, 3H. -CH_3, acetonide), 1.18 – 1.14 (m, 3H, -CH_3, aliphatic), 1.12 – 1.07(m, 6H, -CH_3, isopropyl) ppm.

^{31}P-NMR (202 MHz, $CDCl_3$) δ 3.72, 3.40 ppm.

MS(ES)$^+$ m/z 647.3 $[M+ Na]^+$, 625.3 $[M+ H]^+$

5. NMR data

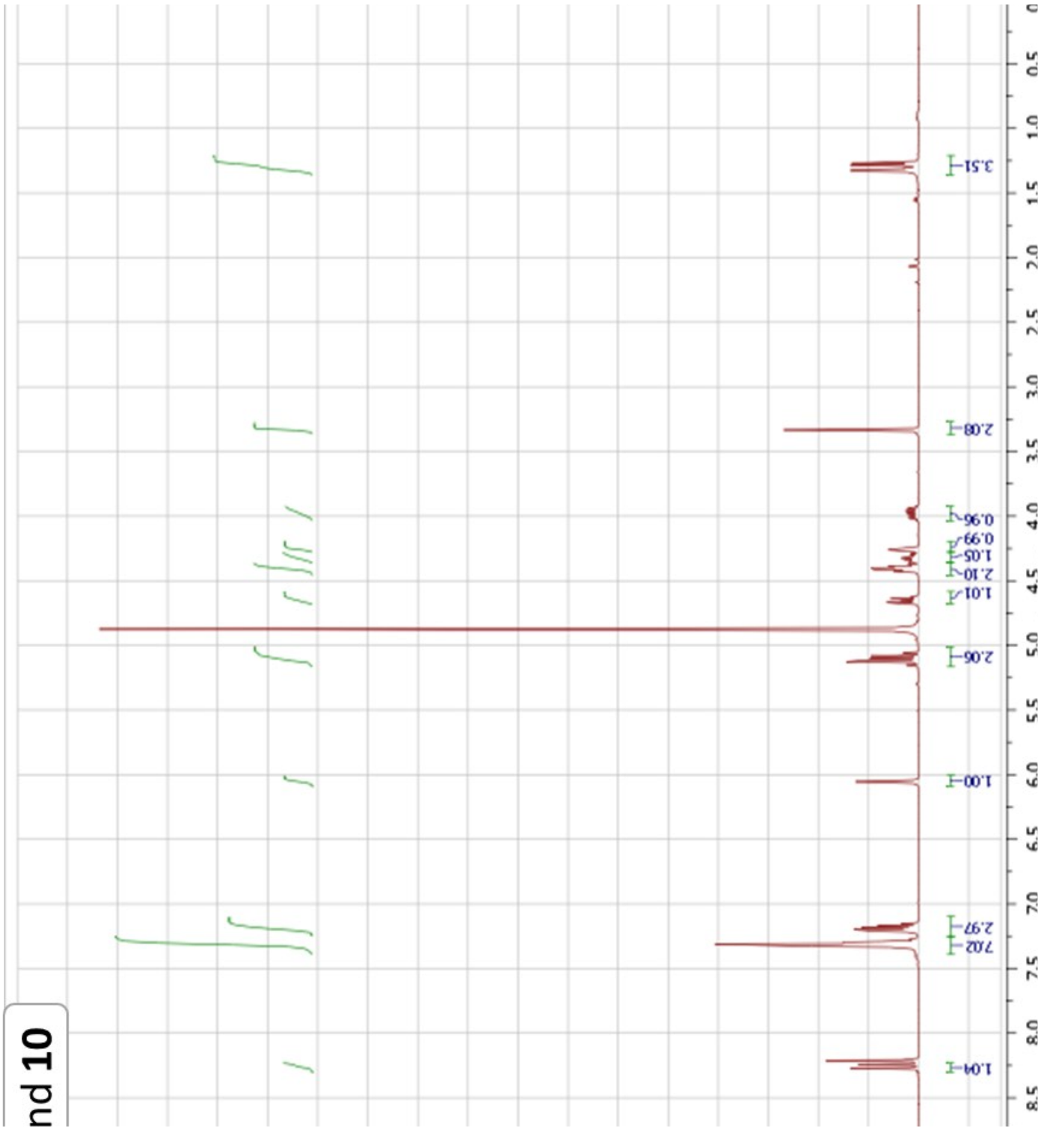

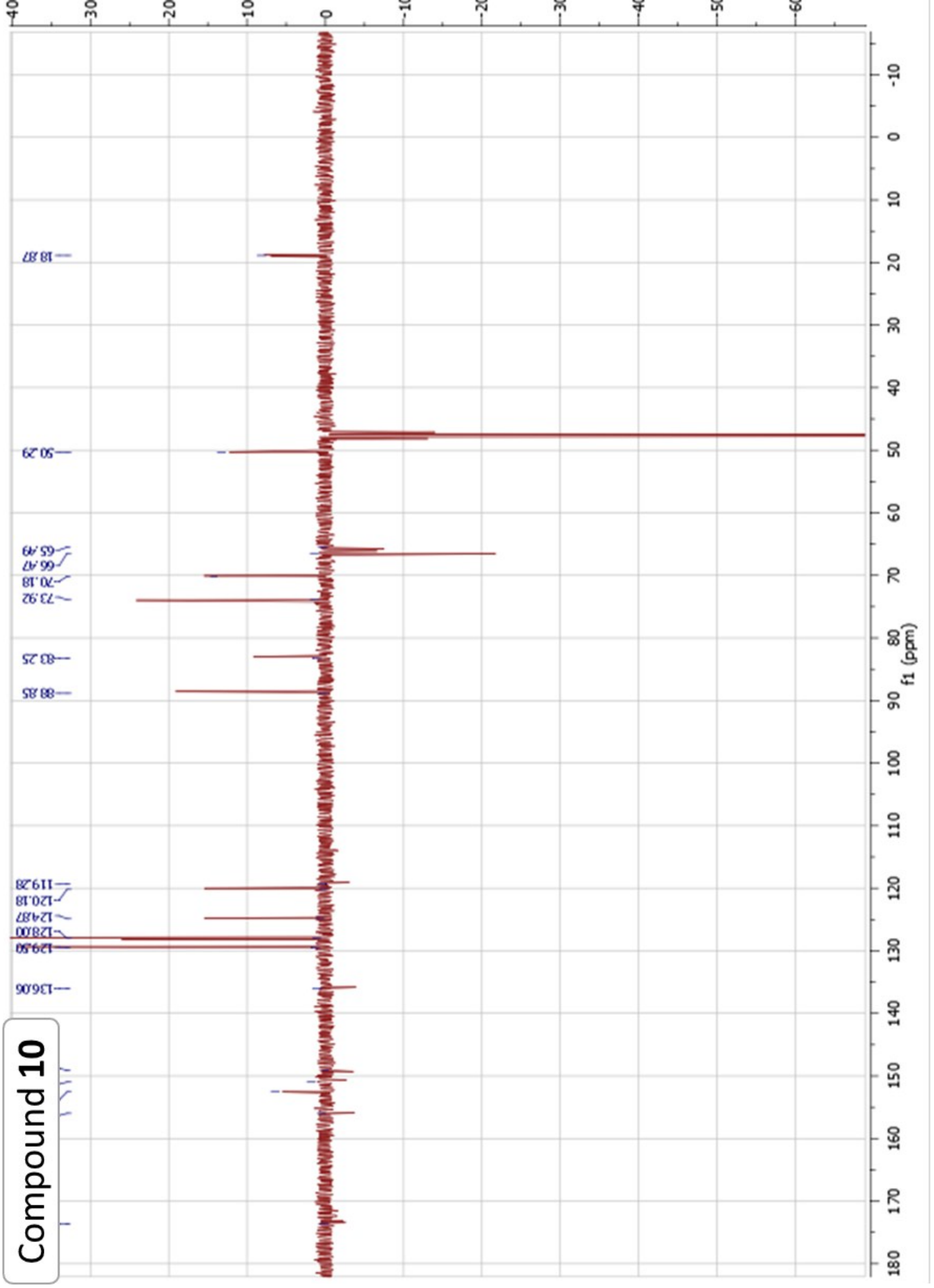
Compound 10
18.87
50.29
65.49
66.47
70.18
73.92
83.25
88.85
119.28
120.18
124.87
128.00
129.80
136.06
f1 (ppm)

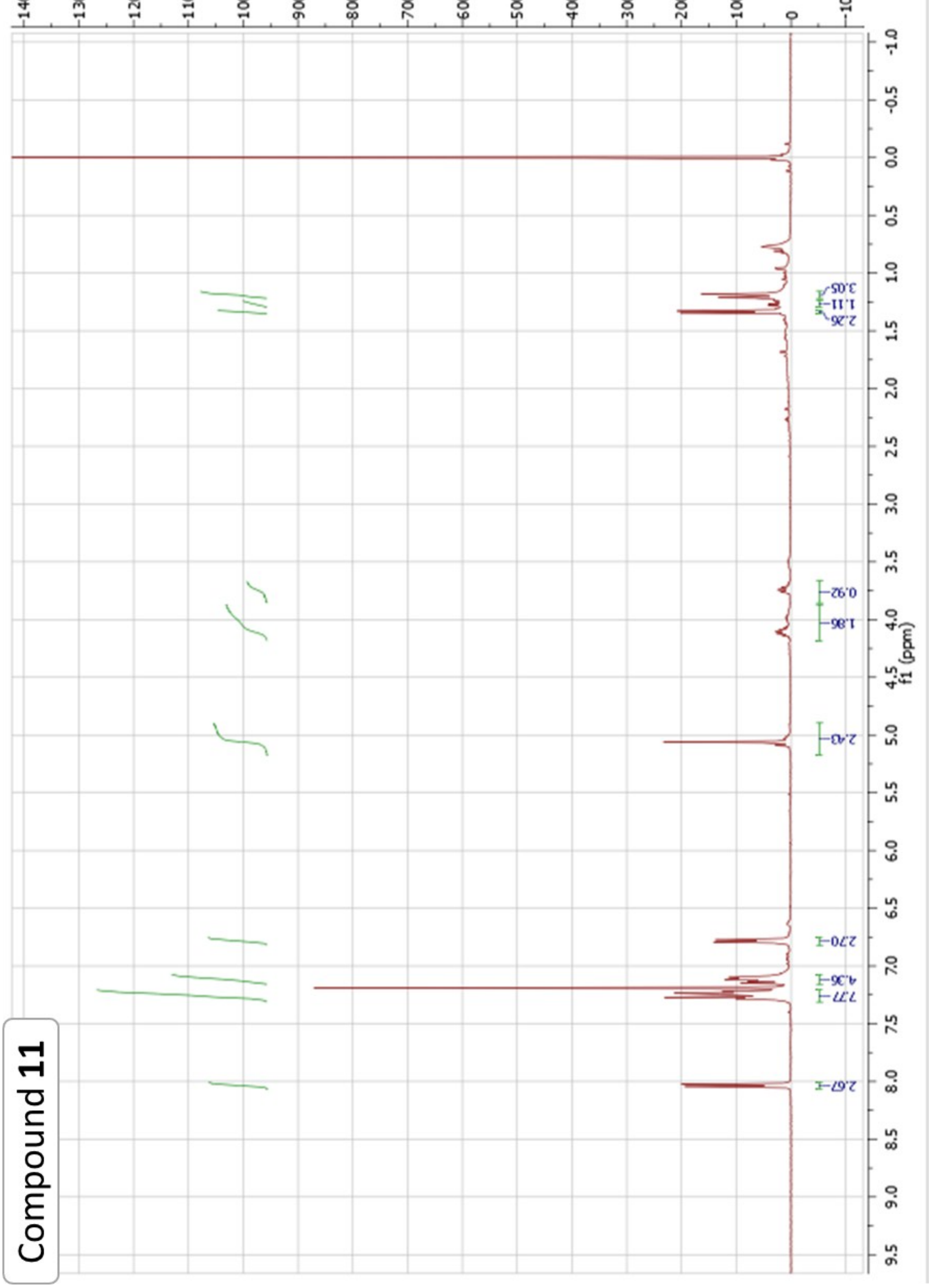
Compound 11
f1 (ppm)
3.05
1.11
2.26
0.92
1.86
2.43
2.70
4.36
7.77
2.67

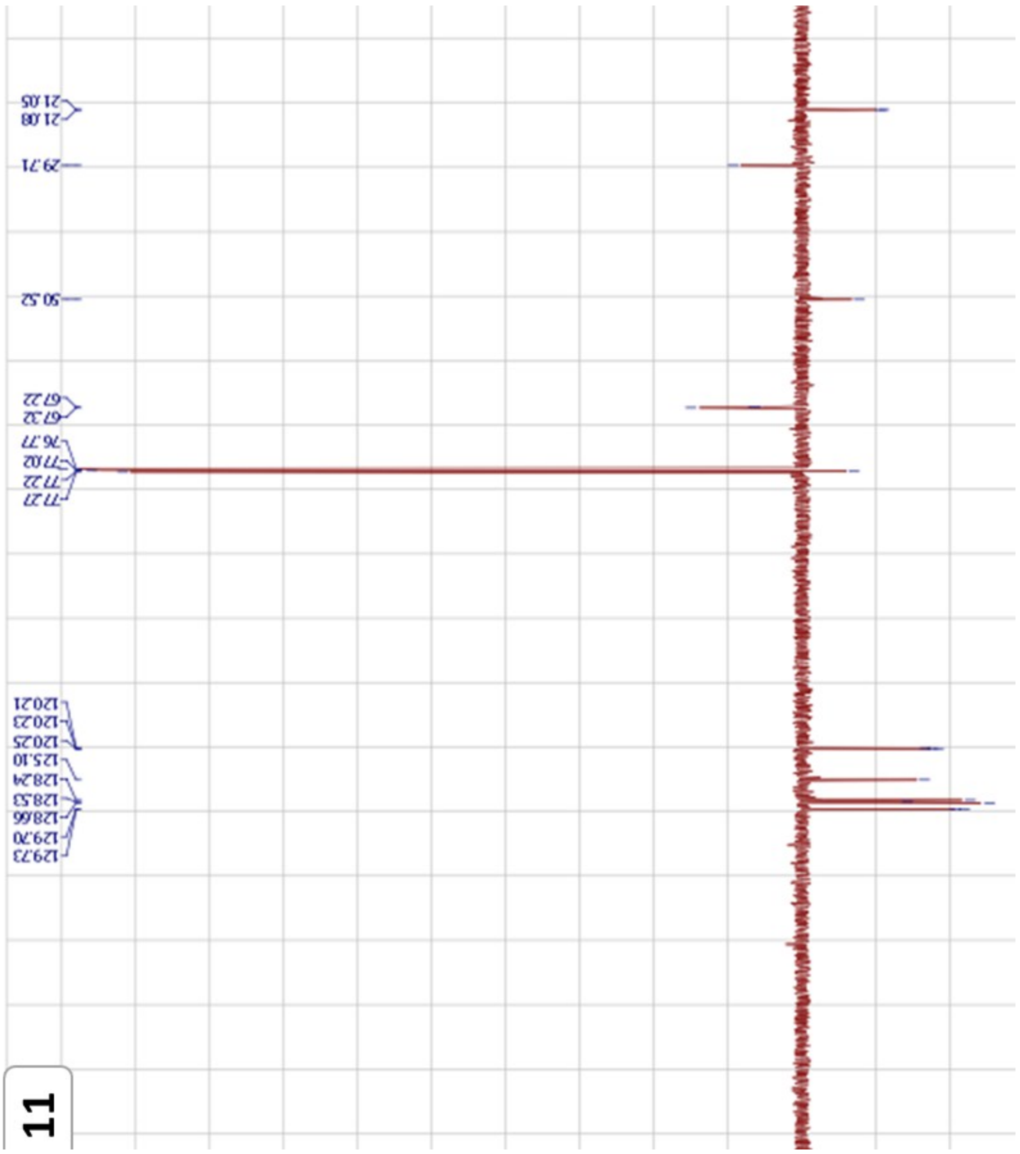
21.05
21.08
29.71
50.52
67.22
67.32
76.77
77.02
77.22
77.27
120.21
120.23
120.25
125.10
128.24
128.53
128.66
129.70
129.73
11

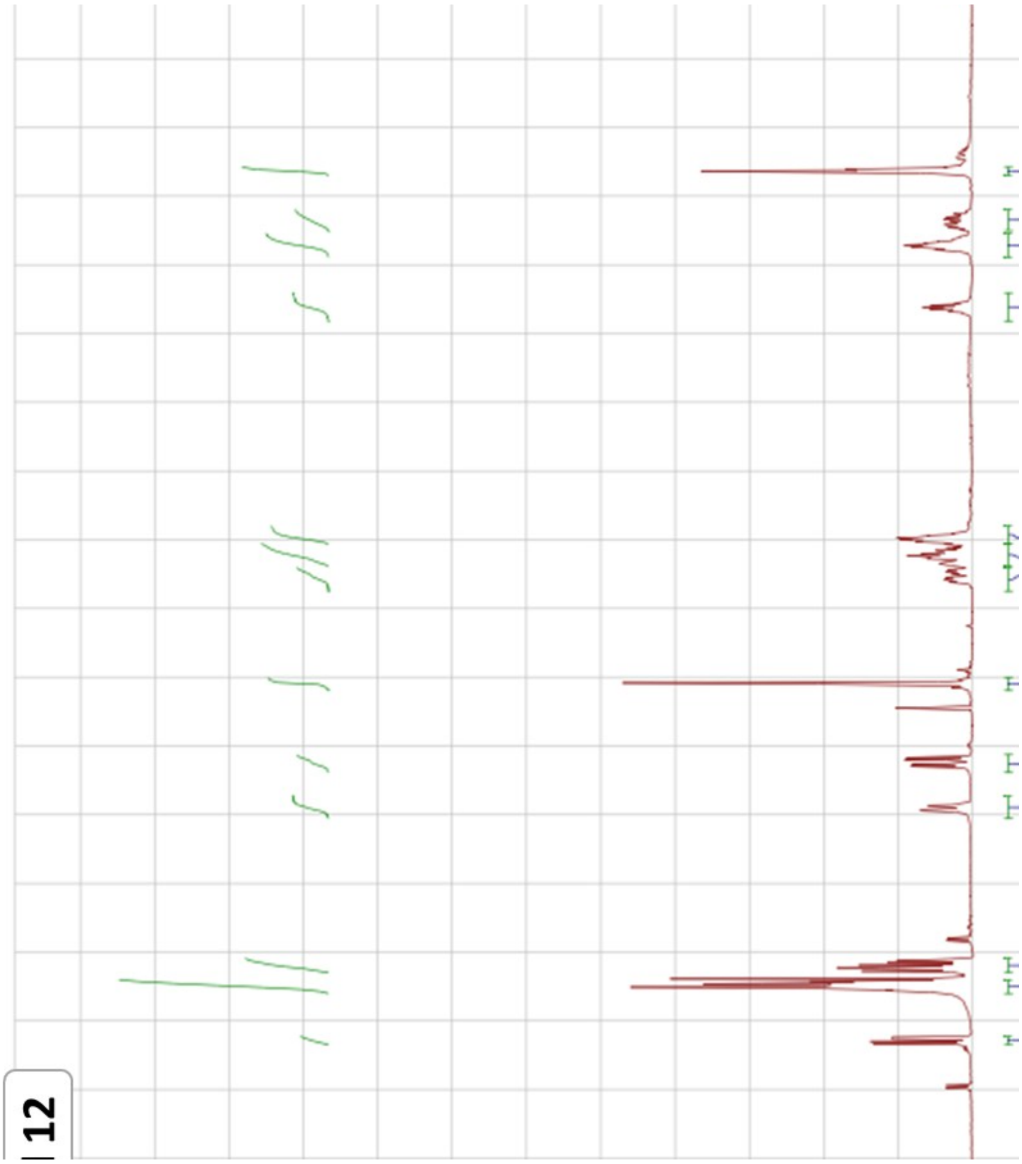
12

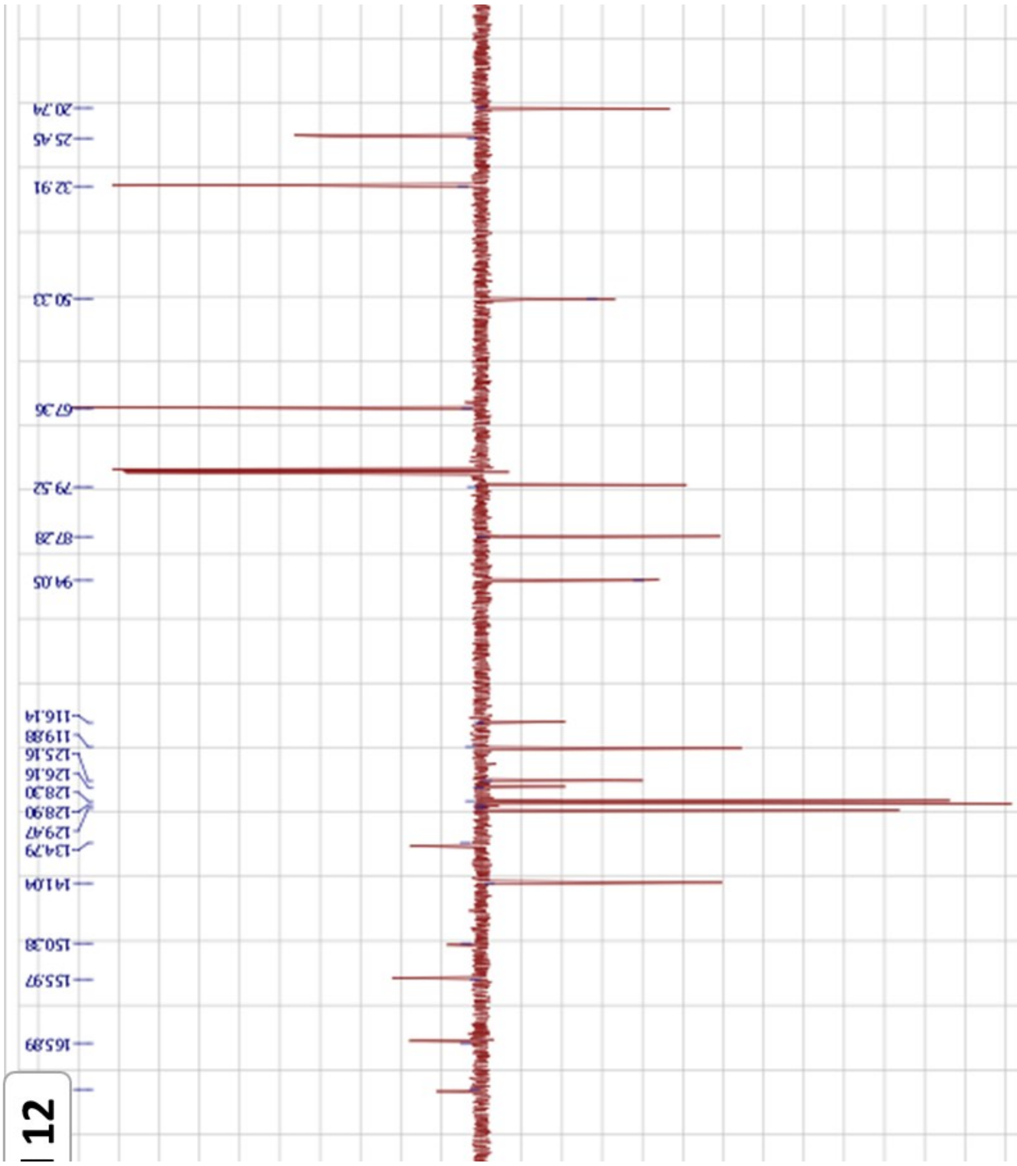
20.74
25.45
32.91
50.33
67.36
79.52
87.28
94.05
116.14
119.88
125.16
126.16
128.30
128.90
129.47
134.79
141.04
150.38
155.97
165.89
12

Compound **13**

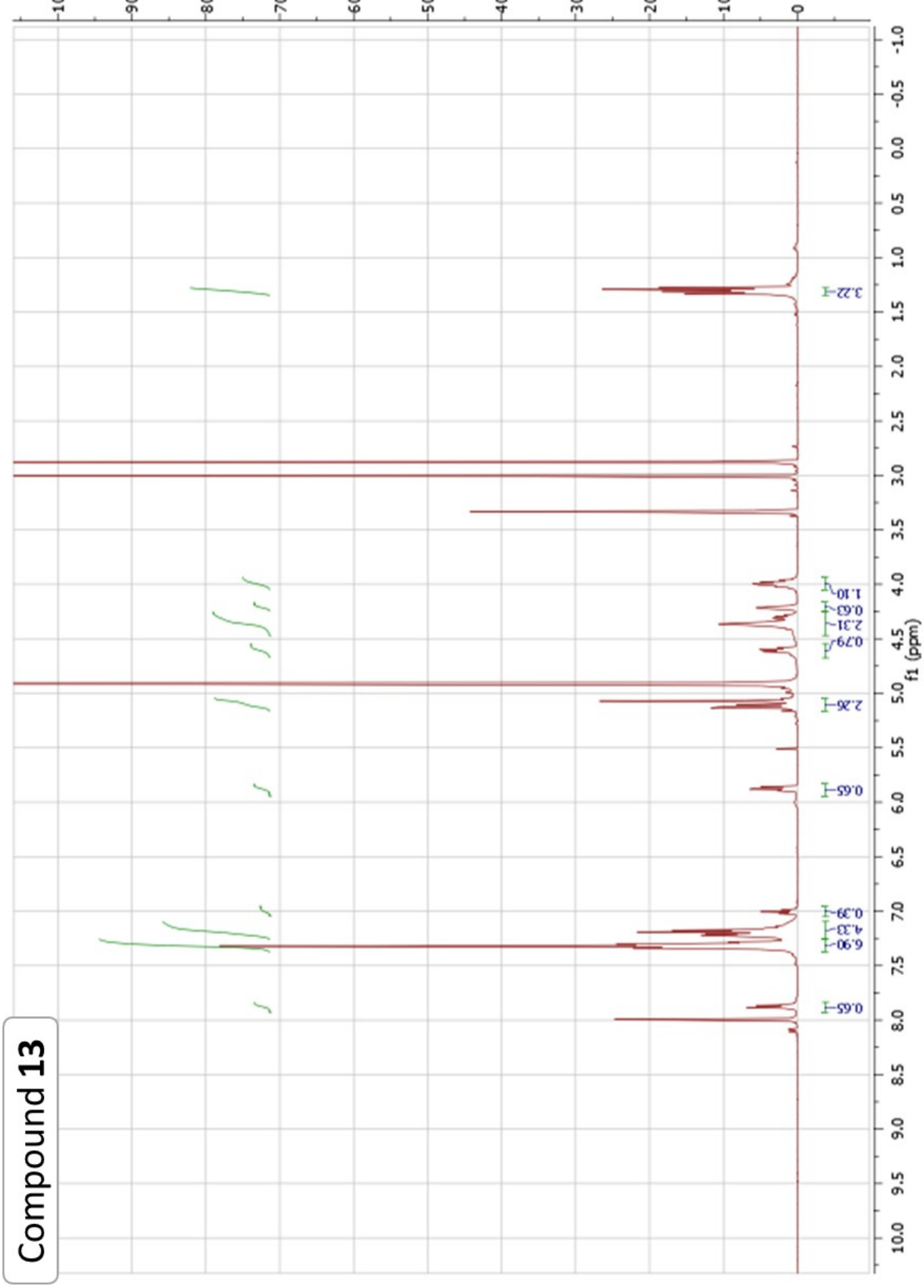

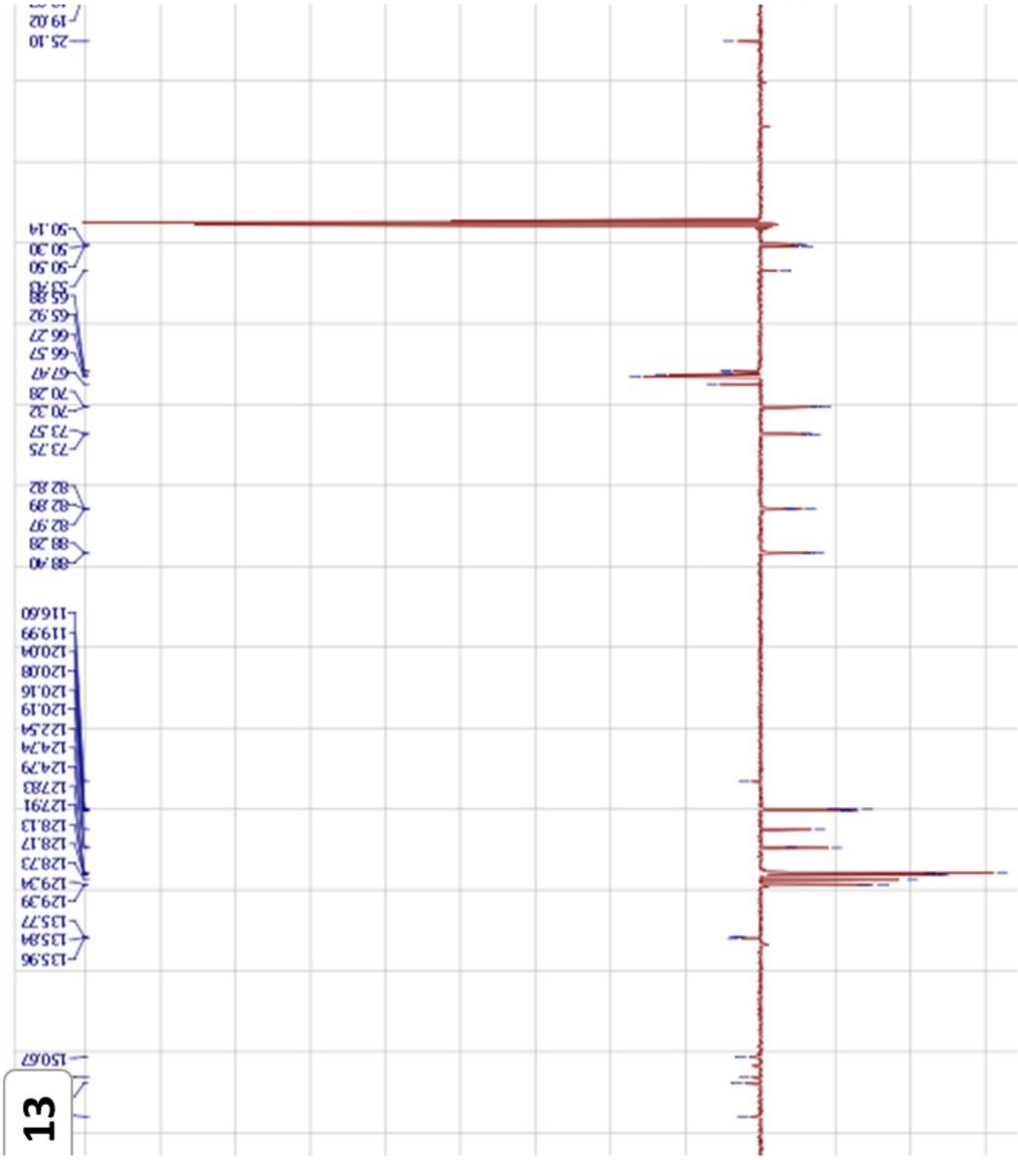

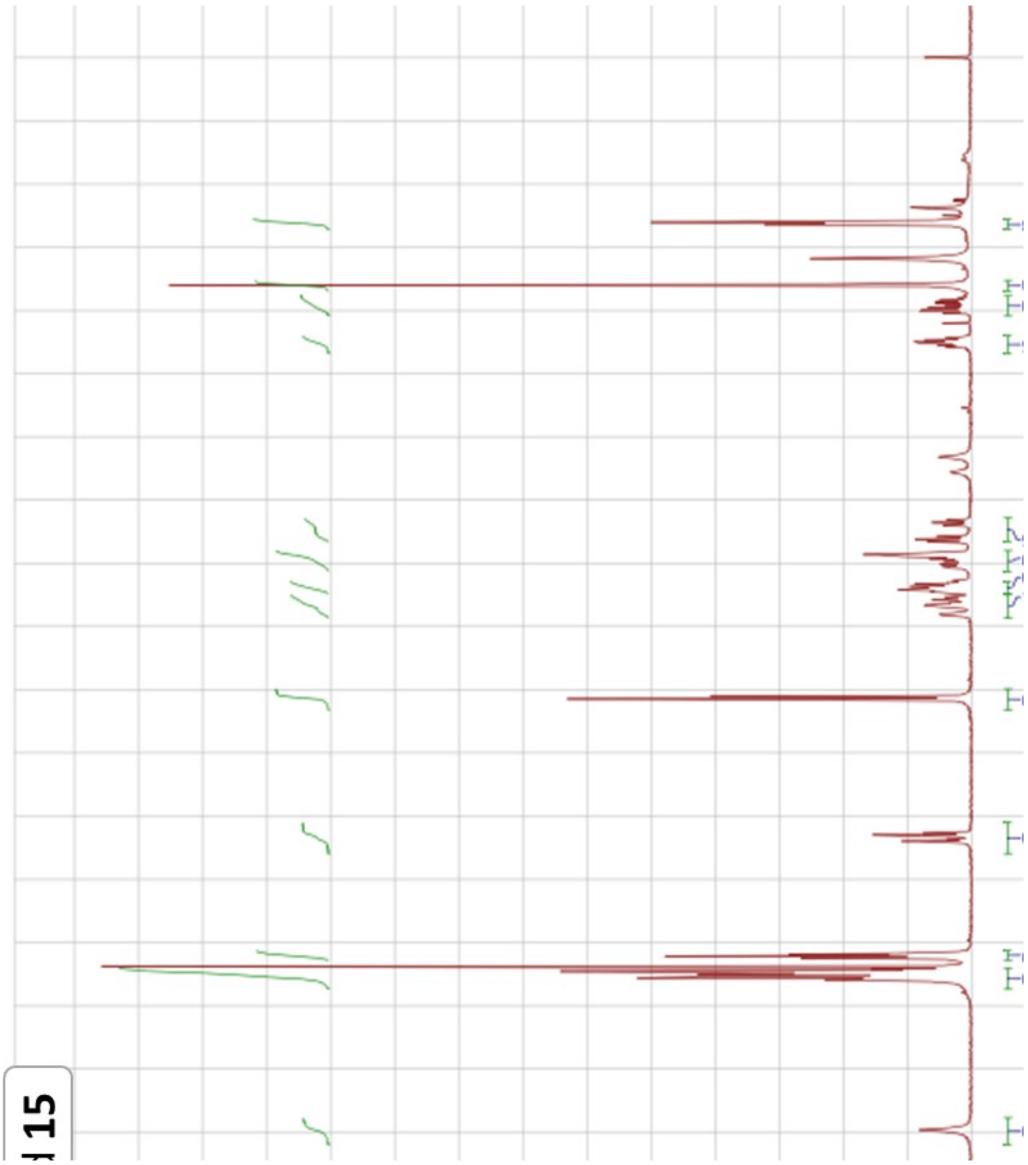
15

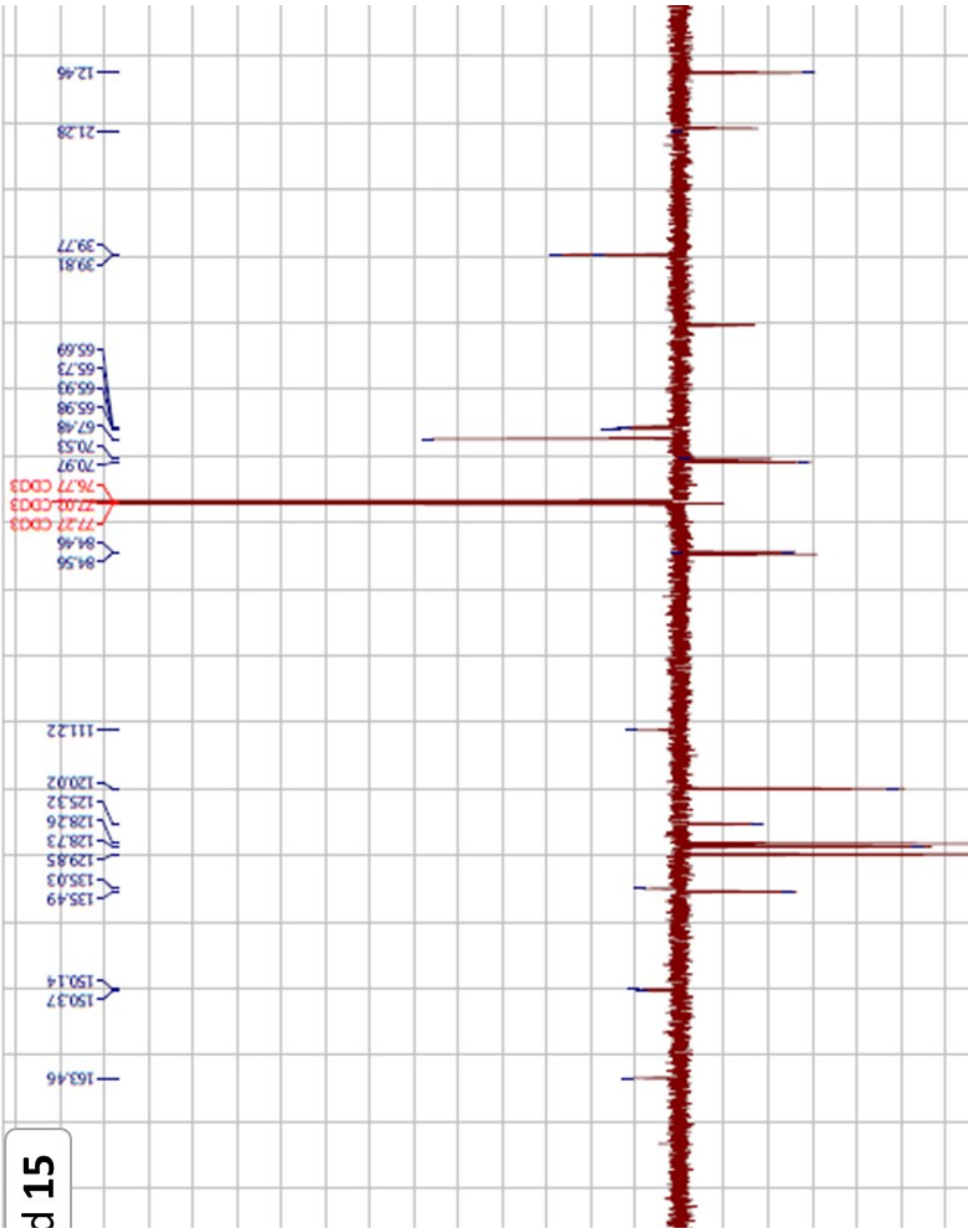
12.46
21.28
39.77
39.81
65.69
65.73
65.93
65.98
67.48
70.53
70.97
76.77 CDCl3
77.02 CDCl3
77.27 CDCl3
84.46
84.56
111.22
120.02
125.32
128.26
128.73
129.85
135.03
135.49
150.14
150.37
163.46
d 15

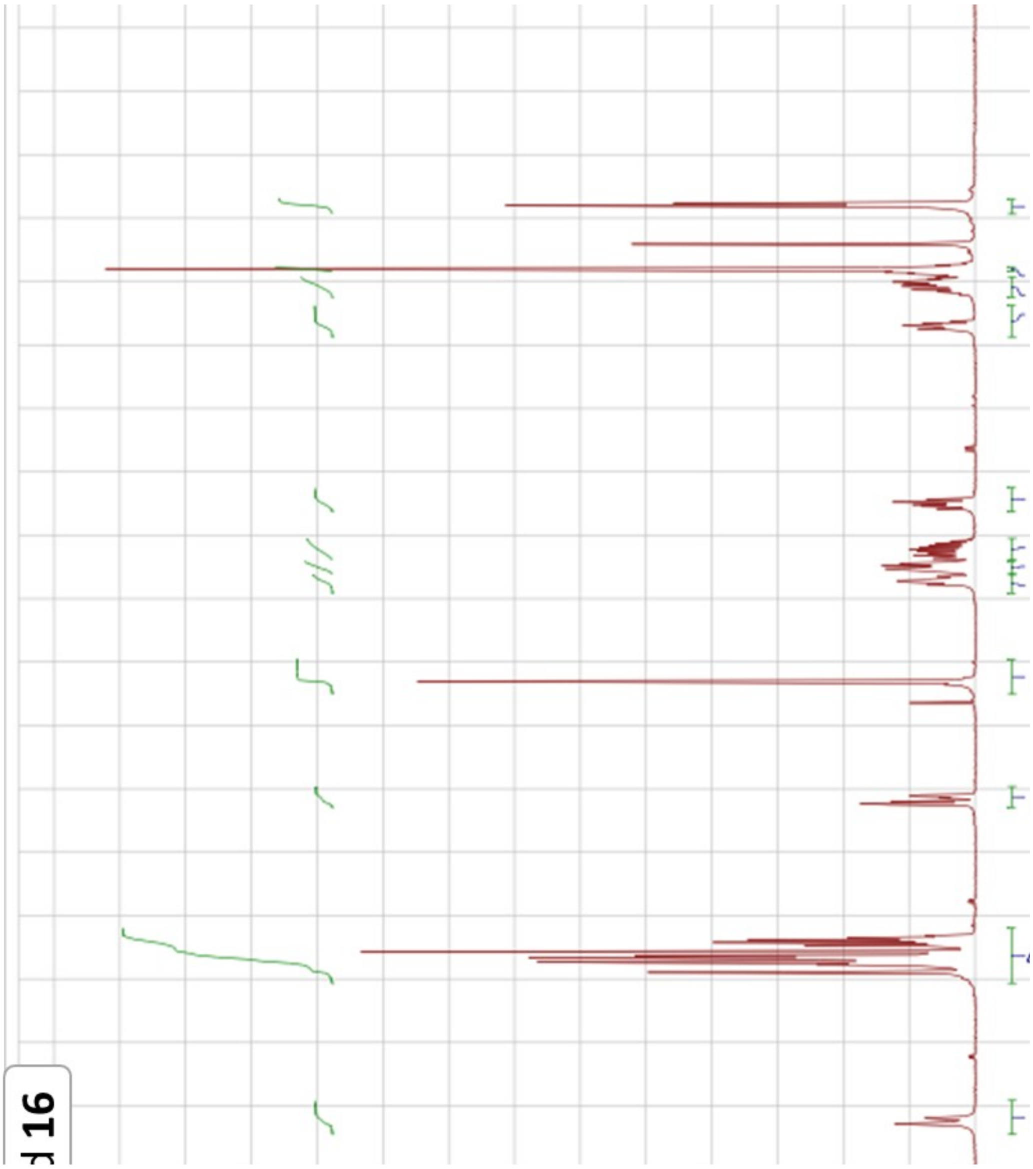
d 16

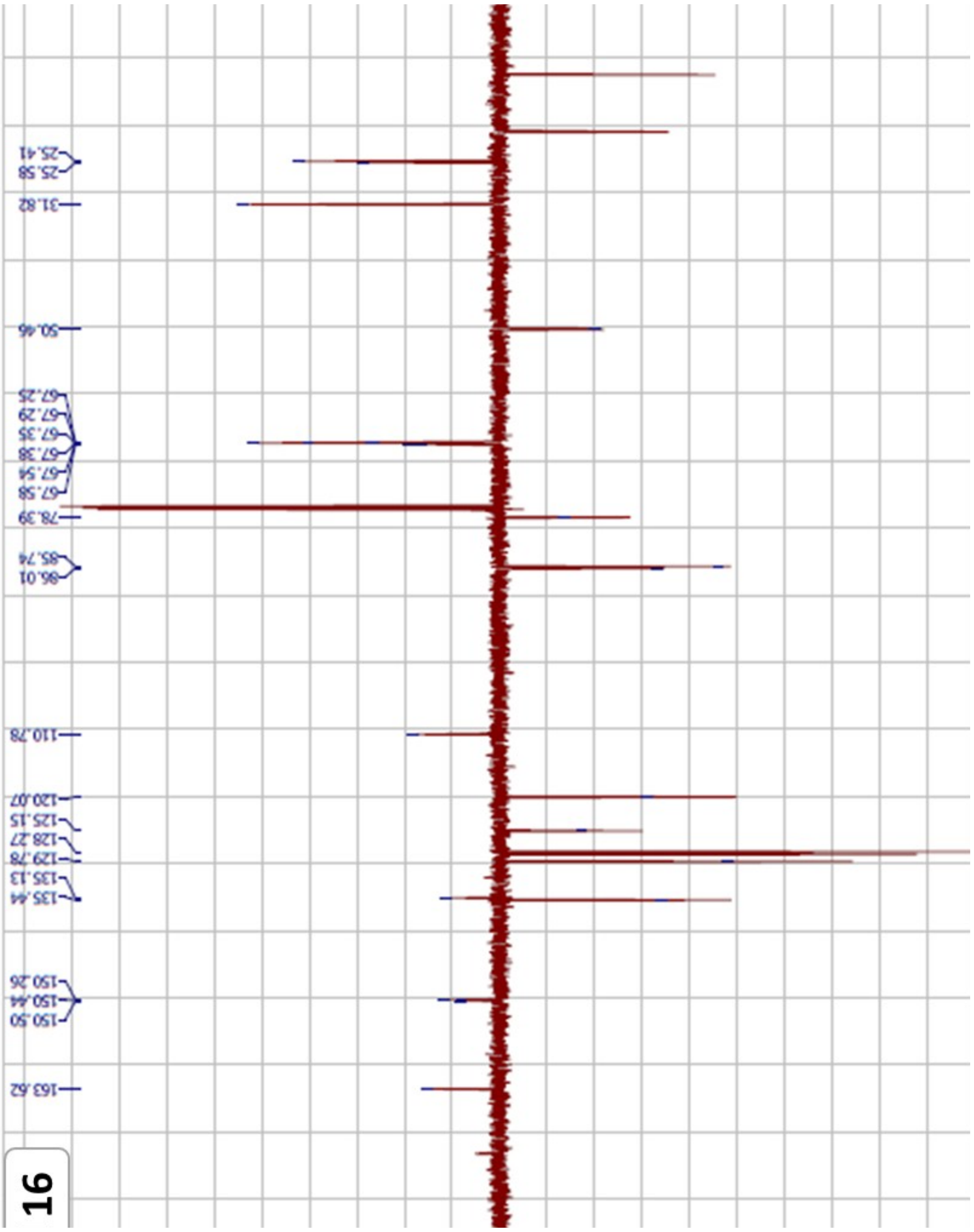
25.41
25.58
31.82
50.46
67.25
67.29
67.35
67.38
67.54
67.58
78.39
85.74
86.01
110.78
120.07
125.15
128.27
129.78
135.13
135.44
150.26
150.44
150.50
163.62
16

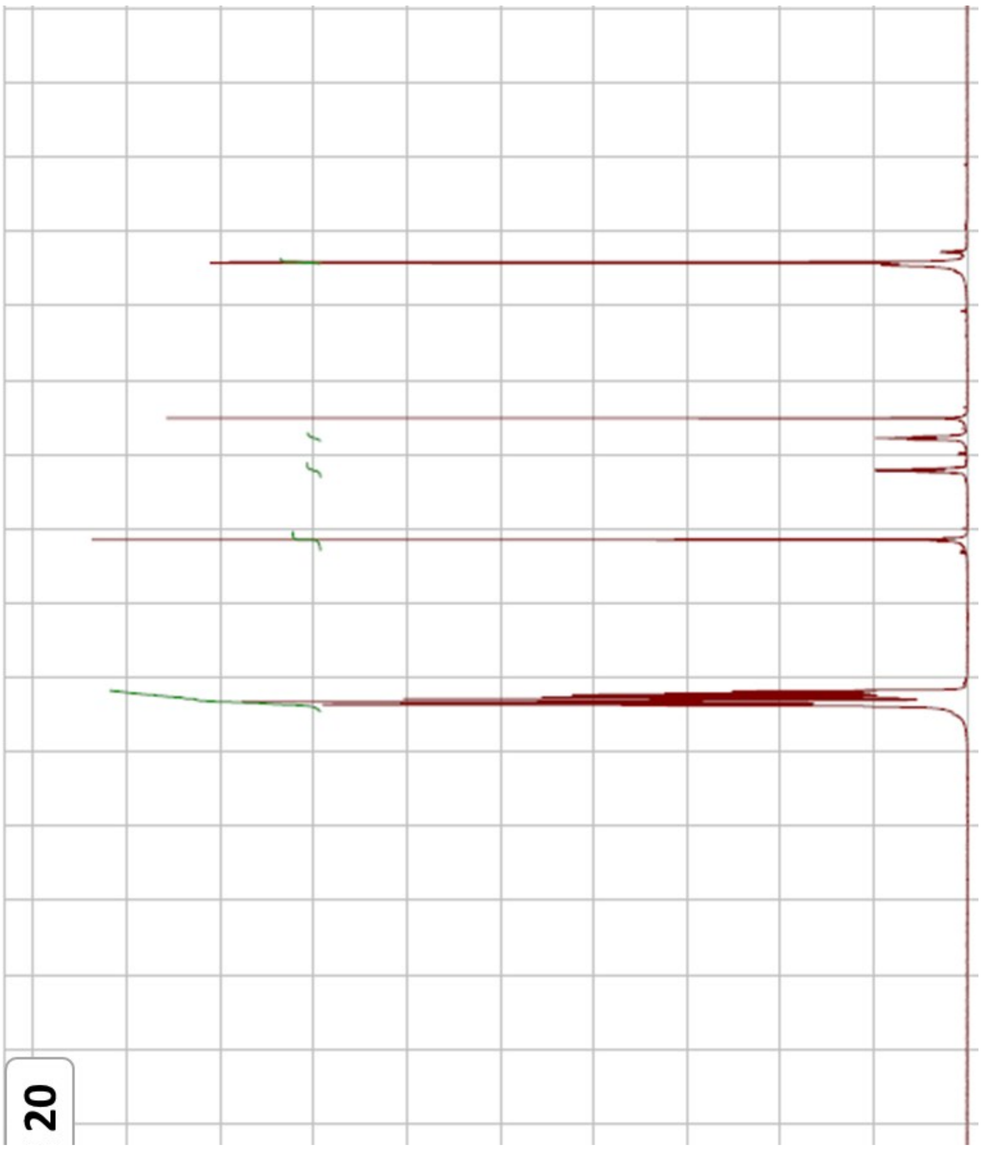
20

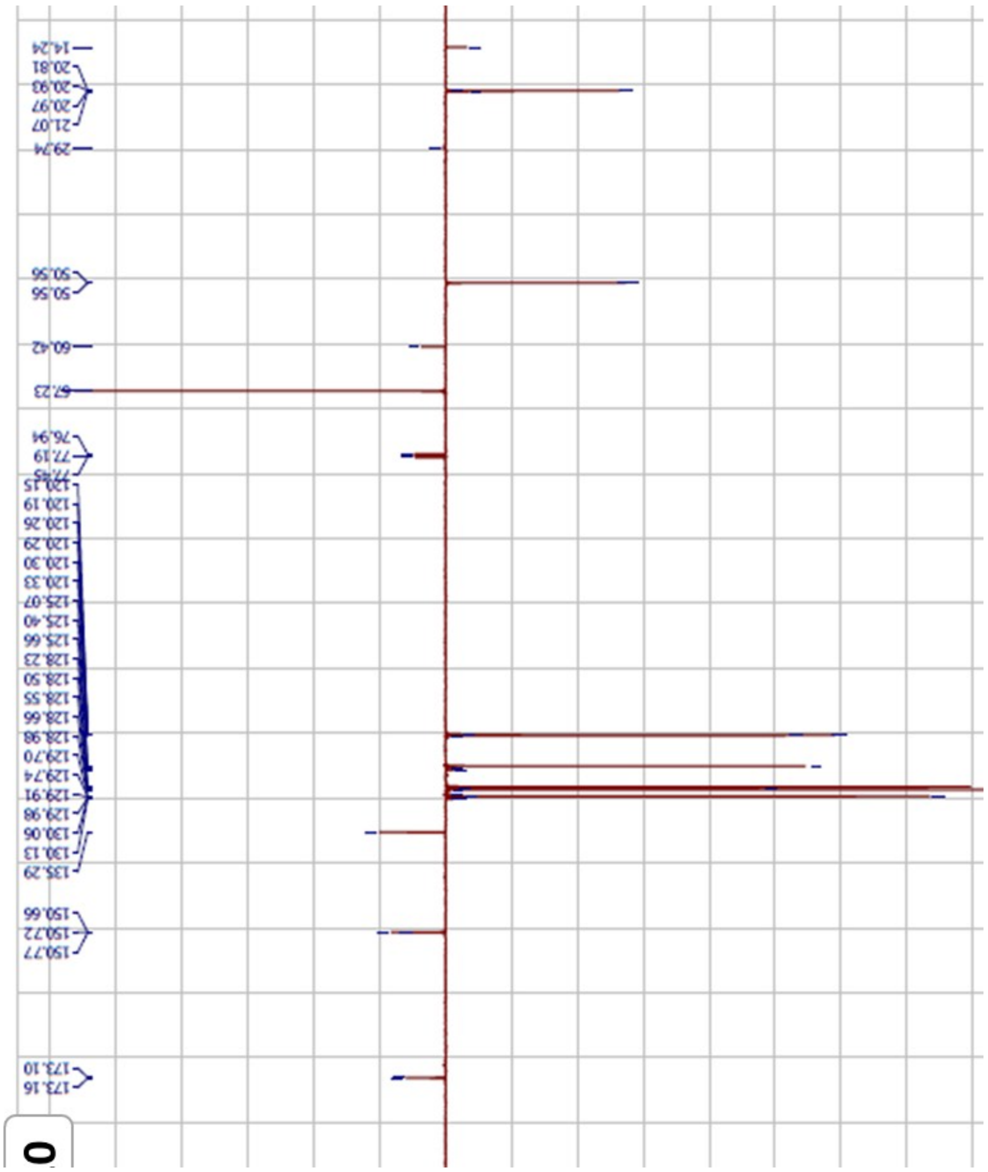
14.24
20.81
20.93
20.97
21.07
29.74
50.56
50.56
60.42
67.23
76.94
77.19
77.45
120.15
120.19
120.26
120.29
120.30
120.33
125.07
125.40
125.66
128.23
128.50
128.55
128.66
128.98
129.70
129.74
129.91
129.98
130.06
130.13
135.29
150.66
150.72
150.77
173.10
173.16
0

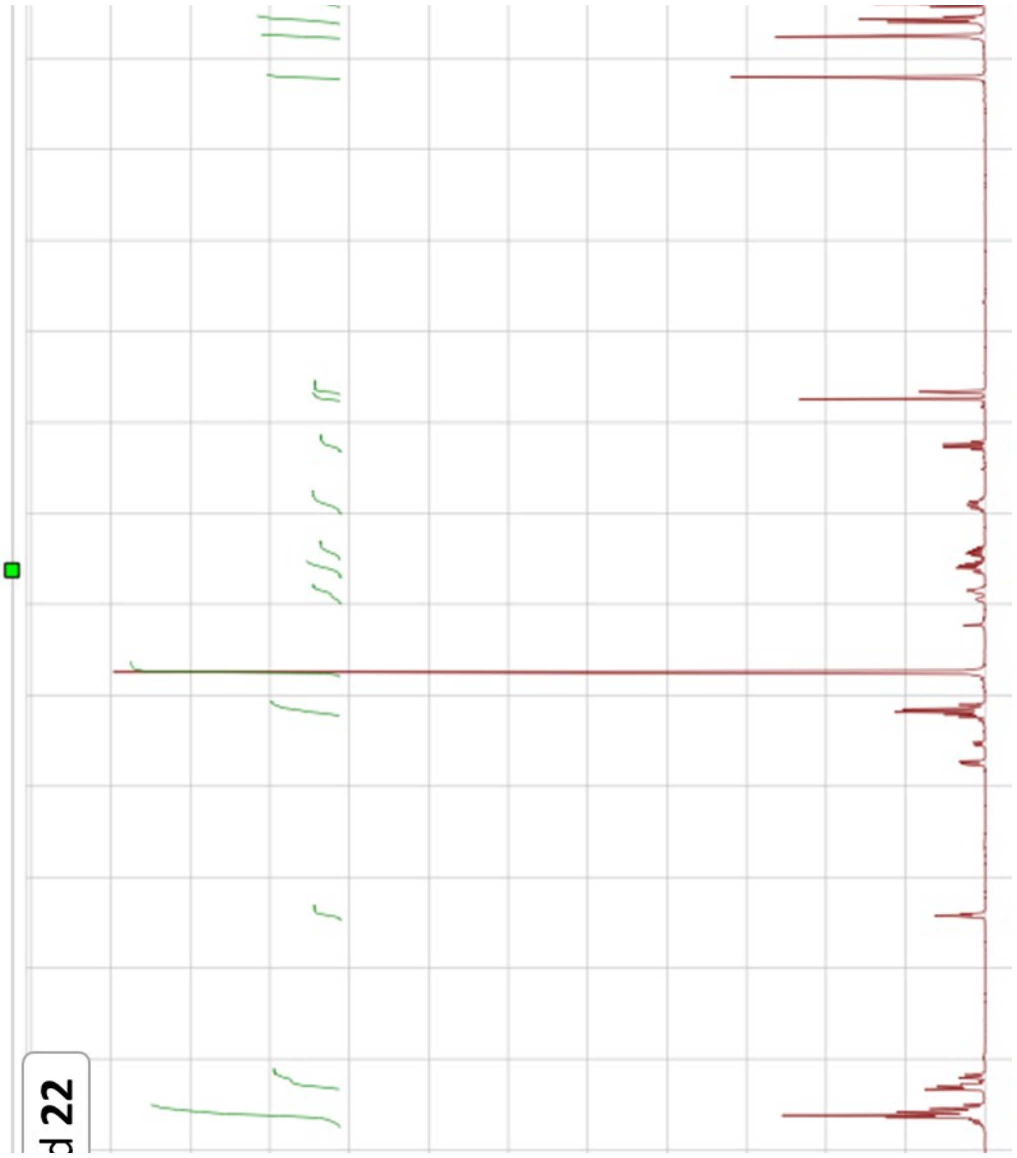
d 22

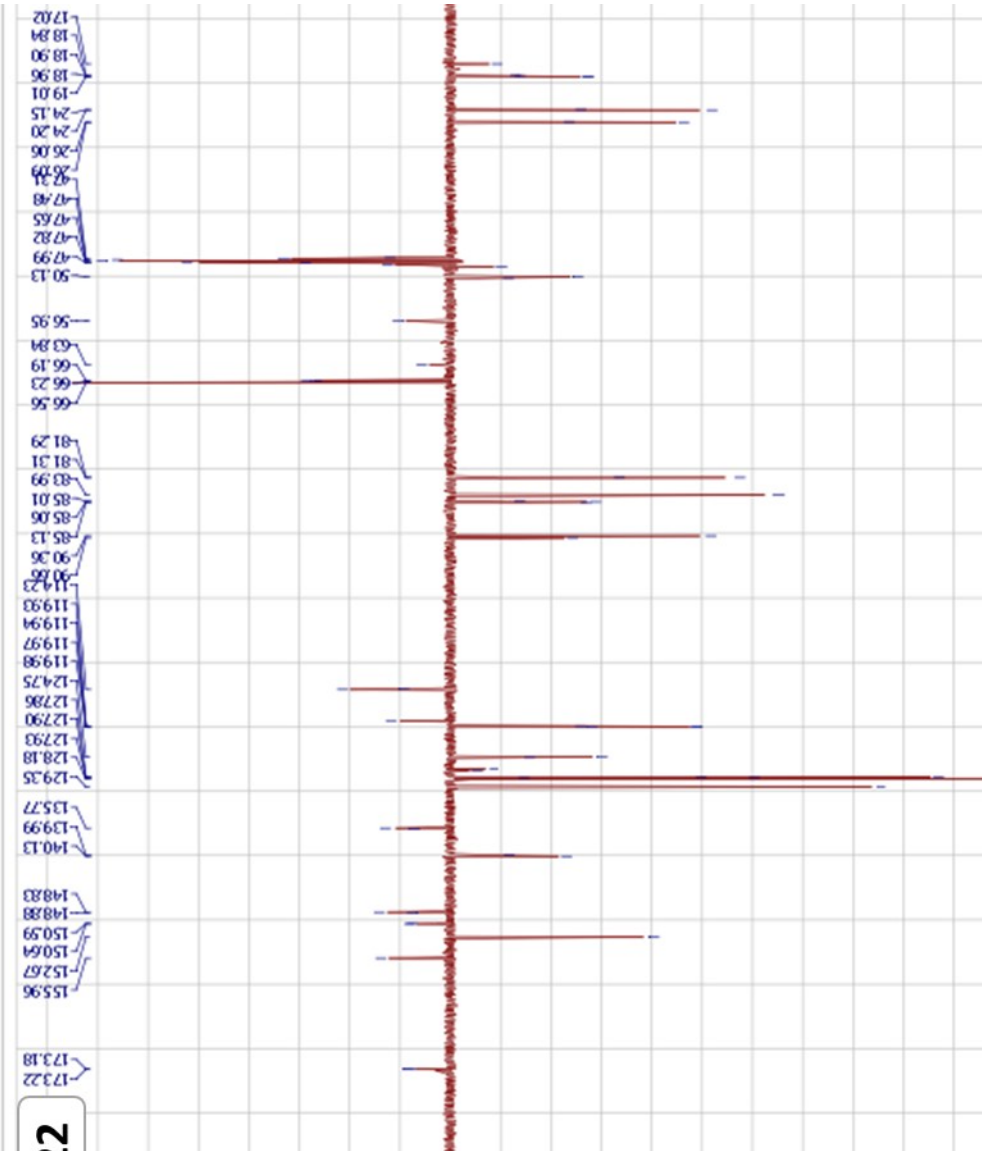
17.02
18.84
18.90
18.96
19.01
24.15
24.20
26.06
47.48
47.65
47.82
47.99
50.13
56.95
63.84
66.19
66.23
66.56
81.29
81.31
83.99
85.01
85.06
85.13
90.36
114.23
119.93
119.94
119.97
119.98
124.75
127.86
127.90
127.93
128.18
129.35
135.77
139.99
140.13
148.83
148.88
150.59
150.64
152.67
155.96
173.18
173.22

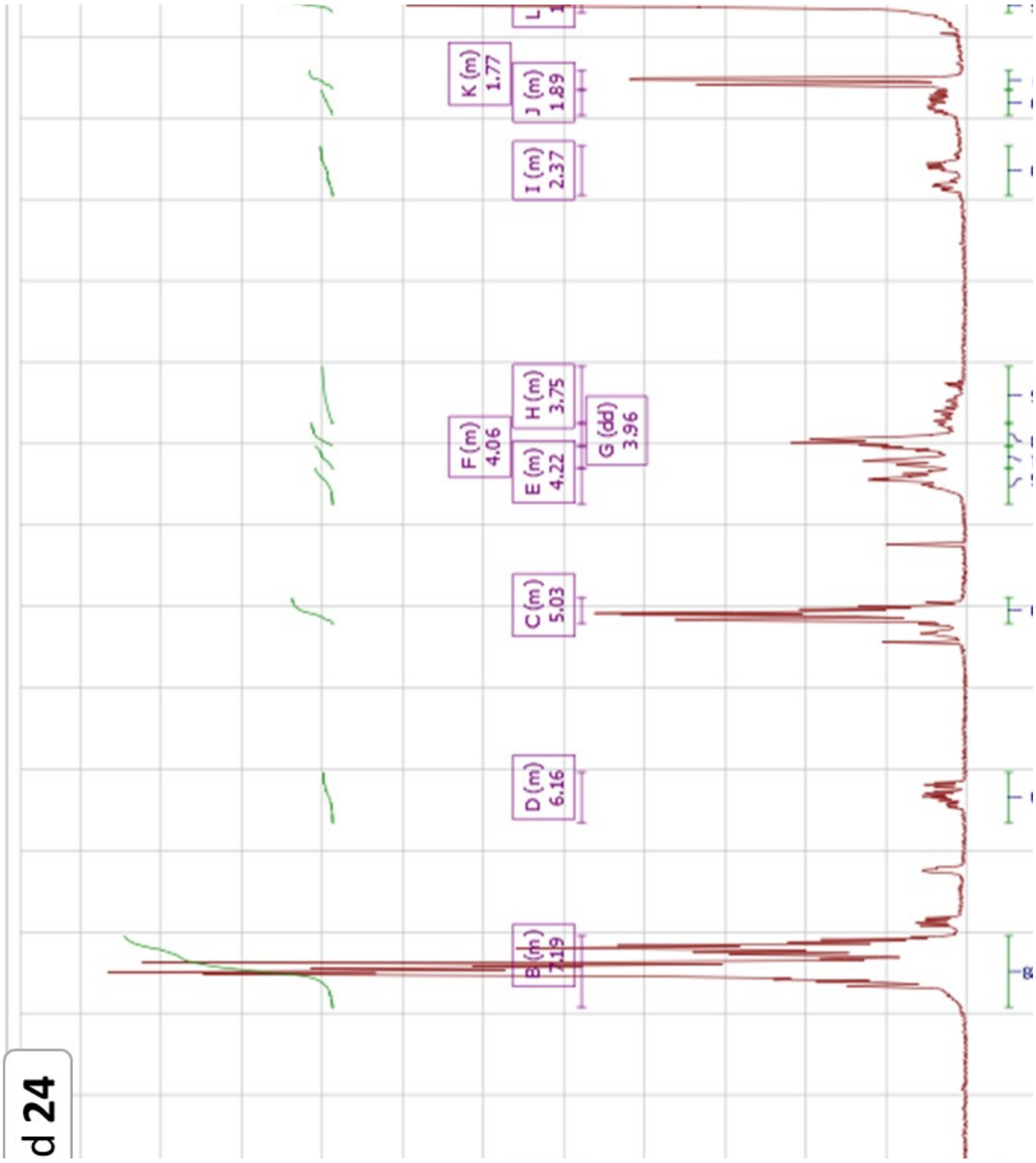
d 24
B (m) 7.19
D (m) 6.16
C (m) 5.03
E (m) 4.22
F (m) 4.06
G (dd) 3.96
H (m) 3.75
I (m) 2.37
J (m) 1.89
K (m) 1.77

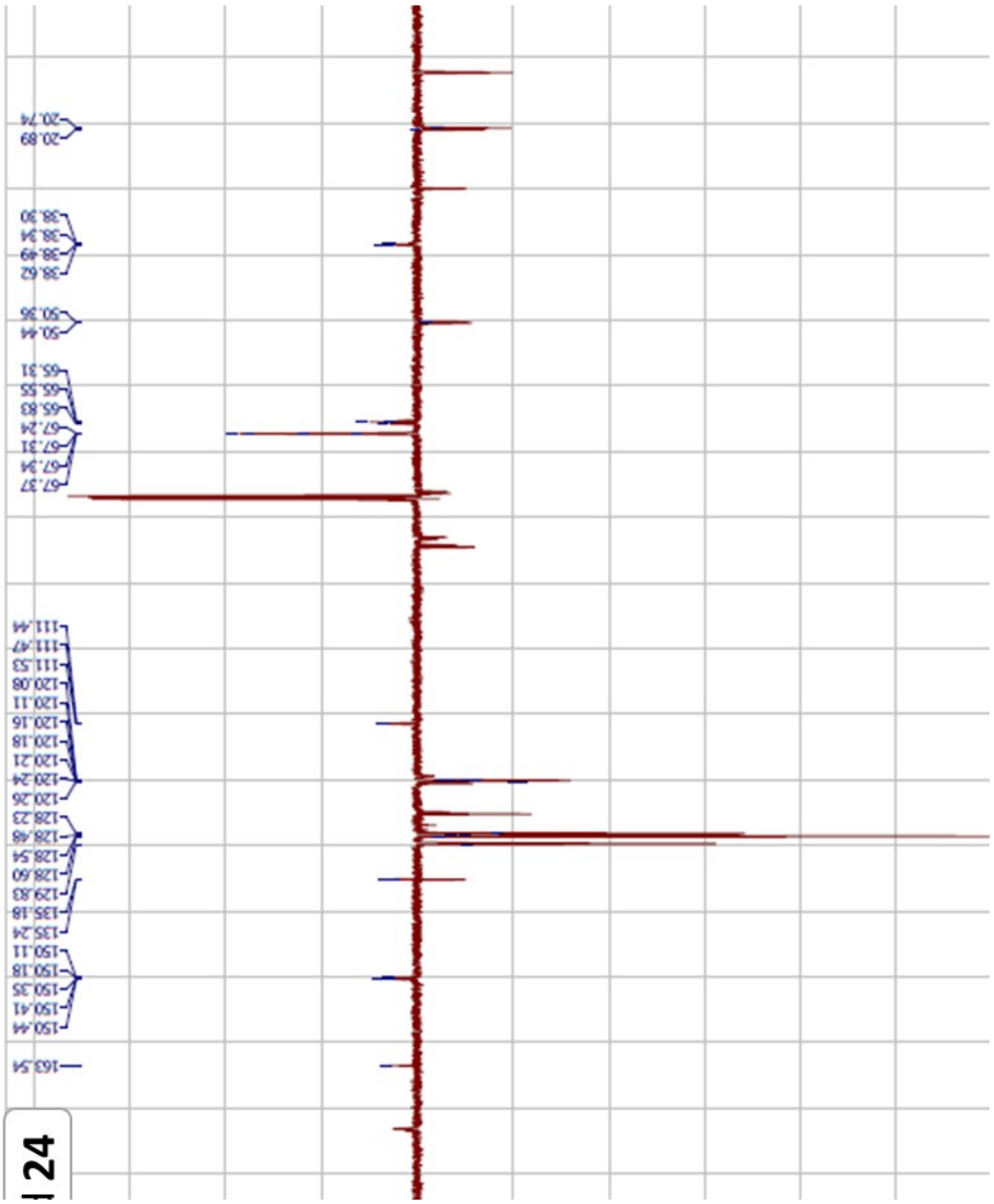

6. Bibliography

1. J. Yuan, Y. Huang, L. Miao, J. Gu, C. Liang, Z, Wang, Z. Sun. Preparation of nucleoside phosphoramidate prodrugs and intermediates. 2017. International Patent, WO/2017/045582.
2. M. Serpi, K. Madela, F. Pertusati, M. Slusarzyk, M. Synthesis of Phosphoramidate Prodrugs: ProTide Approach. Curr. *Prot. in Nucleic Acid Chemistry.* 2013, 15.5.1-15.5.15. DOI: 10.1002/0471142700.nc1505s53.
3. B. S. Ross, P.G. Reddy, H. R. Zhang, S. Rachakonda, M. J. Sofia. Synthesis of diastereomerically pure nucleotide phosphoramidate. *J. Org. Chem.* 2011, 76, 8311–8319. DOI: dx.doi.org/10.1021/jo201492m.
4. C. Mc Guigan, K. Mills, C. Congiatu. Phosphoramidate compounds of nucleoside for use in the treatment of cancer. 2006, International Patent, PCT/GB/2006/000932.
